# Supplementary material for: Exploring data quality and use of the routine health information system in Ethiopia: a mixed-methods study
Source: BMJ Open. 2021 Dec 23;11(12):e050356. doi: 10.1136/bmjopen-2021-050356 (PMC8710857; doi:10.1136/bmjopen-2021-050356)
Supplement: Supplementary data [file bmjopen-2021-050356supp001.pdf]

Immunization team: Data collection tool

I. Quantitative data quality assessment checklist to be used at Woreda health office, Hospital, Health Center and Health Post levels

Data collection Site/level; woreda/ hospital/ health centre/health post _____________ Date of data collection_____________________			
S.N	Description	Response (Yes/No). Note every 'yes' answer will score '1' and '0' for answer 'No'. If not applicable, NA	Comments/clarifications
**A. Demographic/planning**			
1.	Is there a target number of children that the woreda or health facility strives to vaccinate a calendar year or reporting period?	1. Yes<br>2. No<br>3. NA	Obtain the target value and previous review period's annual total. Even if the target is unrealistically high/low, as long as they have set a target they score 1
2.	***Ask this question at woreda health office only***<br>Is the denominator value (for infant immunization) found at the woreda level the same as the one found at the national level?	1. Yes<br>2. No<br>3. NA<br>Denominator set at national ________	Refer in the record in the log book.
3.	Is the proportion of infants for pentavalent3, measles and fully vaccination type known for the woreda and service delivery points	1. Yes<br>2. No<br>3. NA	Strategy; fixed, outreach, mobile. A proportion (%) by each antigen should be available and known
4.	Is there an up-to-date plan for:<br>• The woreda and<br>• The health delivery point (current review period)	1. Yes<br>2. No<br>3. NA	Plan should include the planning process to increase routine vaccinated coverage. It may be integrated with other health services<br>*Please review reference document*
5.	Is there a woreda and health facilities map of catchment areas (including outreach sites) showing health facility providing immunization strategy	1. Yes<br>2. No<br>3. NA	The map should include denominator, target, strategy type
**B. Monitoring and evaluation**<br>Dear participant, now we are going to see the monitoring and evaluation component of immunization data. Thank you for your time**.**			

6.	Is there an up-to-date chart/table of the current review period`s vaccinated coverage displayed any where	1. Yes<br>2. No<br>3. NA	The chart should be displayed in a visible site
7.	Is the completeness of the immunization reports monitored at each reporting level? (woreda and health facilities)	1. Yes<br>2. No<br>3. NA	Refer document for evidence
8.	Does the woreda and health facility monitor reporting timelines for health facility immunization reporting	1. Yes<br>2. No<br>3. NA	Refer document for evidence
9.	Is there an up to date data monitoring of the current review period`s immunization dropout rates?	1. Yes<br>2. No<br>3. NA	Refer document for evidence<br>Can be on the same chart or table as coverage. but score 1 if the health worker can tell you the dropout rate of his health facility
10.	**Ask this question for woreda and health centre only**<br>Is there a routine feedback format for the next lower level	1. Yes<br>2. No<br>3. NA	Refer document for evidence<br>Feedback mean written summary or analysis of immunization data (woreda to health centres, health centers to health posts).<br>Routine means regular feedbacks on monthly bases (not ad hoc). Format means written if distributed or if in from of a meeting is minutes of meeting
11.	Are there regular meetings with health unit workers to discuss immunization performance	1. Yes<br>2. No<br>3. NA	Refer document for evidence<br>Such meetings should occur outside supervisions and involve workers from several health units<br>If yes, how often is this meeting happening???
12.	Are there designated staffs responsible for reviewing the quality of data (i.e., accuracy, completeness and timeliness) received from sub-reporting levels (e.g., service points)?.	1. Yes<br>2. No<br>3. NA	Designated staffs can be PMT or HMIS focal assigned to do this task

13.	Does all designated staff have received training on the data management processes and tools?	1. Yes<br>2. No<br>3. NA	
14.	**Ask this question for woreda and health centre only**<br>Are supervision activities are conducted weekly, monthly or quarterly?	1. Yes<br>2. No<br>3. NA	Refer document for evidence.<br>A written schedule of supervision that includes visiting every health unit with as specified period of time. Supervision must include immunization, record should include list of health facilities, date visited and by whom. Check core indicator
15.	Is there a mechanism of monitoring vaccine stock outs at of health facility level?	1. Yes<br>2. No<br>3. NA	The manager should able to say (based on written information) weather one health unit has encountered a vaccine stock out. If no vaccine stock out reported, ensure that the monitoring is possible and done. Stock out means interruption in vaccine supply (for any vaccine)
**C. Recording practices**<br>Dear volunteer, now we are going to switch the discussions to immunization data recording practices.			
16.	Are vaccines receipt and issues recorded in vaccine ledger book	1. Yes<br>2. No<br>3. NA	If no Ledger book, or inappropriate record keeping of vaccine receipts and issues, score 'No'. If it is not sufficient to have bin card, but an appropriate carder system will suffice provided it is correctly maintained, stored and archived
17.	Is the current ledger book up to date for all vaccines reviewed (including dry supplies)	1. Yes<br>2. No<br>3. NA	Refer document for evidence
18.	Were immunization forms (tally sheets, reporting formats, vaccine requisition formats) sufficiently available in the visited health units during the review period	1. Yes<br>2. No<br>3. NA	Check the availability of formats. If one of the format is missing, score 'zero'

, et al. BMJ Open 2021; 11:e050356. doi: 10.1136/bmjopen-2021-050356

19.	***<u>Ask this question at service delivery level only</u>***<br>Are there tally sheets for infant vaccinations on the desk (or easily available)?	1. Yes<br>2. No<br>3. NA	Check the availability of the tally sheet. If tally sheets completed by month and not each immunization session, ensure that tally sheet has month/review period clearly marked. Check the numbers given during the last immunization session
20.	Do tally sheets have entries for the last immunization day?	1. Yes<br>2. No<br>3. NA	<u>Review the document</u>
21.	Did the individual reporting and recording form (tally sheets) from the respective health units use the same form/format for the current review period	1. Yes<br>2. No<br>3. NA	If there was an official change in the report format, a four month flexibility should be allowed (mix of old and new forms for a maximum of 4 months)
22.	***<u>Ask this question at service delivery level only</u>***<br>Are registers (or pre-printed forms) used for recording individual information about child immunization	1. Yes<br>2. No<br>3. NA	Check the child registers. These may be child health cards, if cards are kept in health facility
23.	***<u>Ask this question at service delivery level only</u>***<br>Can a child`s vaccination history be easily and rapidly retrieved in the registers	1. Yes<br>2. No<br>3. NA	Review the document. A new dose should not be entered as a complete new entry but entered in the location where previous doses have been entered. Score 0 if register is used as a new entry for any immunization
24.	***<u>Ask this question at service delivery level only</u>***<br>Was the correct individual record completed for every vaccination observed	1. Yes<br>2. No<br>3. NA	Observe immunization of five (among infants) and check the child card. If no observation score, NA. If you can observe five immunizations, then skip child health card exercise.
25.	***<u>Ask this question at service delivery level only</u>***<br>Was the correct date to return given for every vaccination observed	1. Yes<br>2. No<br>3. NA	Observe immunization of five (among infants) and check the child card. If no observation score, NA.

, *et al. BMJ Open* 2021; 11:e050356. doi: 10.1136/bmjopen-2021-050356

26.	Does the woreda office and health facility stamp or write the date of report from health facility is received at woreda level on the report	1. Yes<br>2. No<br>3. NA	Needs to be answered as such by the woreda medical officer or person and health facility manager in charge of immunization services (i.e., is the monitoring of timeliness systematic and organized). This must be the first date, the report was seen (received) at woreda or health facility level, not the date the report was processed
**D. Storing/Reporting Practices**<br>Dear respondent, we are now going to discuss on immunization data storing and reporting process			
27.	Have all available health facility reports from the period previous to the last one been processed	1. Yes<br>2. No<br>3. NA	Processing means, entering data in to whatever system they use (electronic or paper). Ideally there should be a written instructions about the process and procedures
28.	Are all the health facility reports available for the entire review period	1. Yes<br>2. No<br>3. NA	Monthly or quarterly depending on country. Record in the logbook all the dates that are missing.
29.	Is there a procedure/ system of dealing with late reporting	1. Yes<br>2. No<br>3. NA	Information coming late should be sent to the national level. The explanation provided should be in line with national guidelines. If no national guideline, the system should be functional.
30.	Does each health facility have its own file or sub file?	1. Yes<br>2. No<br>3. NA	Review the document<br>Storage should facilitate retrieval and monitoring (and be well organized). If the storage is filled by date only (not by health centre) score yes if any report search is easy

31.	Does each health facility papers files are filed by date?	1. Yes<br>2. No<br>3. NA	Review the document
32.	Is there a written back up procedure for every reporting to the next level?	1. Yes<br>2. No<br>3. NA	If not computerized score 'na'
33.	Can the official immunization tabulation for the review period be reproduced from an archive electronic file?	1. Yes<br>2. No<br>3. NA	If not computerized score 'na' official immunization tabulation means final summary of review period data. Archive electronic file means stored file from hard disk/diskettes
34.	Is the data of printing/production on every tabulation/chart or, if the data is archived, the date the archive file was created?	1. Yes<br>2. No<br>3. NA	If not computerized score 'na'

II. Data verifications at the woreda health office and service delivery points

A. Immunization service data comparison between reports and records

Date ____________________ Time _________________________ Health institution code ___________

Description	Immunization data reports and records in the review period								
	Pentavalent 3			Measles			Fully vaccinated		
Recount results from the periodic reports sent from service sites to the Woreda and compare to the value reported by the Woreda.	Month 1	Month 2	Month 3	Month 1	Month 2	Month 3	Month 1	Month 2	Month 3
Reported by the health centre/hospital (By EPI unit)									
Recounted health center/hospital (tally sheet)									
Recounted health center/hospital (registration book)									
Ratio of reported over recorded									

Aggregated PHCU reported to woreda (including health posts)									
Reported by health post to health centre									
Recounted health post (tally sheet)									
Recounted health post (registration book)									
Ratio reported over recorded									

B. Immunization logistics data reports and records

Date ________________________ Time _____________________ Health institution code ___________________

Antigen	Logistics data in the review period											
	Month 1				Month 2				Month 3			
	Beginning balance	Quantity received	Loss/adj	End-ing balance	Beginning balance	Quantity received	Loss/adj	Ending balance	Beginning balance	Quantity received	Loss/adj	End-ing balance
Pentavalent												
PCV												
Rota												
BCG												
IPV												
BOPV												
Measles												

C. Immunization data reports timeliness and completeness check list*

Date _______________________________ Time ________________________ Health institution code __________

Health facilities	Reporting period			Agreed reporting time line	Total completeness	Total timeliness
	Month 1/date of the report received	Month 2/date of the report received	Month 3/date of the report received			
Health centre 1						
Health centre 2						

, et al. BMJ Open 2021; 11:e050356. doi: 10.1136/bmjopen-2021-050356

Health centre 3						
Health centre 4						
Health post 1						
Health post 2						
Health post 3						
Health post 4						
Health post 5						
Hospital						
Total received this month (NO)						
Total received this month (%)						
Cumulative completeness (%)						
Total on time this month (NO)						
Cumulative timeliness (%)						

*Adapted from the immunization data quality self-assessment (DQS) tool (WHO, 2005)

Key

- Insert the date the health facility reports were received at the health institution. If a report is received after the deadline, enter the date in red.
- Total completeness or timeliness: refers to the reporting completeness of the health facility. Cumulative completeness: reports received up to that month divided by reports expected up to that month.

  Cumulative timeliness: reports received on time up to that month divided by reports expected up to that month.

, *et al. BMJ Open* 2021; 11:e050356. doi: 10.1136/bmjopen-2021-050356
