# Supplementary material for: Exploring data quality and use of the routine health information system in Ethiopia: a mixed-methods study
Source: BMJ Open. 2021 Dec 23;11(12):e050356. doi: 10.1136/bmjopen-2021-050356 (PMC8710857; doi:10.1136/bmjopen-2021-050356)
Supplement: Supplementary data [file bmjopen-2021-050356supp002.pdf]

Data collection tool for malaria indicators
Structured questionnaire for health facility

**CONFIRMED AND SUSPECTED MALARIA CASES**				
1	Does this facility diagnose and treat malaria?	1. Yes ☐	0. No ☐	
**SOURCE DOCUMENTS AND REPORTS**				
2	Does this facility report malaria data to a reporting system?	1. Yes ☐	0. No ☐	
3	To which of the following reporting systems does the facility report malaria data?	1. Health Management Information System (HMIS)<br>2. Public health Emergency Management (PHEM)<br>3. Malaria program<br>4. Nongovernmental organizations or institutions<br>5. Other reporting system (Specify)		
**4**	What is the source document used by this facility for monthly/weekly reporting of malaria?	1. Laboratory register<br>2. OPD register<br>3. Inpatient register<br>4. Emergency register<br>5. Other (specify)		
**REPORT TIMELINESS**				
**5**	Is there a deadline for submission of the malaria report by the health facilities?	1. Yes<br>2. No		
**6**	If yes, what is the deadline (date of month)?<br>(Write the end date of the deadline)	Reporting deadline:____________________		
**7**	Does the health facility record the dates of submission of monthly/weekly malaria reports to the Woreda/Zone/Region (see logbook/computer)?	1. Yes<br>2. No		
**IF AVAILABLE, REVIEW THE RECORDS AND CHECK THE DATES OF SUBMISSION FOR THE THREE REVIEW MONTHS**				
**8**	Month 1	Reported date ____________		
	Month 2	Reported date____________		
	Month 3	Reported date _____________		
**9**	If any discrepancy is observed between Reporting deadline and each reporting date, what are the possible reason?	1. Shortage of man power<br>2. Interruption of electricity/computer<br>3. Shortage of reporting formats<br>4. Competing priority (Campaign)<br>5. Reporting date aligned with holiday<br>6. Other (Specify)		If there is no discrepancy skip to Q10
**10**	What method of reporting system does the facility use	1. Paper based system<br>2. Electronic system		

		3. Both	
**DATA USE FOR DECISION MAKING**			
**11**	Does the health facility have analysed malaria data (e.g., summary tables, charts, maps)?	0. No<br>1. Yes, observed paper-based<br>2. Yes, observed electronic<br>3. Both	
**12**	Does the health facility uses analysed malaria data for decision making	1. Yes<br>2. No	If no Skip to Q14
**13**	If Yes, for what purpose	1. Performance management (Planning and reporting)<br>2. Supply requesting and reporting<br>3. Priority setting<br>4. Monitoring targets<br>5. Advocacy<br>6. Other (Specify)	Skip to Q16
**14**	If no, what are the possible bases for decision making in your health facility	1. Personal preference for decision making<br>2. Superior directives<br>3. What was done in last year<br>4. Funding directives from higher level.<br>5. Political considerations<br>Other (Specify)	
**15**	If No, what are the possible reasons for not using malaria data for decision making in your health facility	1. Poor data quality<br>2. Unavailability of data<br>3. Negative Perception<br>4. Other (Specify)	

**REVIEW THE SOURCE DOCUMENTS AND MONTHLY REPORT FOR SUSPECTED MALARIA CASE**				
**1**	Please confirm the availability of **source documents** for malaria for month 1 to month 3. If available, please **Recount** the number of **Suspected malaria cases** recorded in the **source document** month 1 to month 3.	**(A) Source documents available**	**(B) Recount the number of Suspected malaria cases in the source documents (if none, please enter 0)**	**(C)Record monthly Suspected malaria cases from monthly report**

	Months	Yes, available and complete*	Yes, available but partly** complete	Yes, available but no data recorded	No		
**01**	**Month 1**	1	2	3	0		
**02**	**Month 2**	1	2	3	0		
**03**	**Month 3**	1	2	3	0		
**REVIEW THE SOURCE DOCUMENTS AND MONTHLY REPORT FOR CONFIRMED MALARIA CASE**							
**2**	Please confirm the availability of **source documents** for malaria for month 1 to month 3. If available, please **Recount** the number of conf. malaria cases recorded in the **source document** month 1 to month 3.	**(A) Source documents available**				**(B) Recount the number of conf. malaria cases in the source documents (if none, please enter 0)**	**(C)Record monthly confirmed malaria cases from monthly report**
	**Months**	**Yes, available and complete***	**Yes, available but partly** complete**	**Yes, available but no data recorded**	**No**		
	**Month 1**	1	2	3	0		
	**Month 2**	1	2	3	0		
	**Month 3**	1			0		
**REVIEW THE SOURCE DOCUMENTS AND MONTHLY REPORT FOR TOTAL MALARIA CASE**							
**1**	Please confirm the availability of **source documents** for malaria for month 1 to month 3. If available, please **Recount** the number of **total malaria**	**(A) Source documents available**				**(B) Recount the number of total malaria cases in the source documents (if none, please enter 0)**	**(C)Record monthly total malaria cases from monthly report**

	**cases** recorded in the **source document** month 1 to month 3.						
	**Months**	Yes, available and complete*	Yes, available but partly** complete	Yes, available but no data recorded	No		
	**Month 1**	1	2	3	0		
	**Month 2**	1	2	3	0		
	**Month 3**	1	2		0		
**Take the last 15 entries recorded in the Lab register for each reporting period and check if all the data elements relevant to the selected indicator are filled in.**<br>*COMPLETE means that the source document contains the data relevant to the selected indicator.<br>**PARTLY: the register is available but some information is missing.							
2	If the source document (Lab register) is not available, what are the possible reasons?	1. Storage or archiving problems<br>2. Absence of designated staff<br>3. Stock out of source document<br>4. Other (specify): ___					
3	If the source documents (Lab register/) are partially complete or has no data, what are the possible reasons for the missing data?	1. Staffing issue(s)(shortage, absence)<br>2. Not understanding the data element<br>3. Presence of other vertical reporting requirement<br>4. Data burden (too much data elements to be recorded)<br>5. The recording tool is not designed as user friendly<br>6. Other (specify): ___					
**DATA COMPLETENESS**							

5	If the monthly report for malaria is not available, what are the possible reasons?	1. Storage or archiving problems<br>2. Absence of designated staff<br>3. Stock out of source document<br>4. Other (specify): ___________________________
6	If the monthly report of malaria is partially complete or has no data, what are the possible reasons for the missing data?	1. Staffing issue(s)(shortage, absence)<br>2. Not understanding the data element<br>3. Presence of other vertical reporting requirement<br>4. Data burden (too much data elements to be recorded)<br>5. The recording tool is not designed as user friendly<br>6. There is no client to be reported<br>7. Other (specify): ________________________________
**DISCREPANCIES**		
**7**	If there was a discrepancy observed between the **source document** and the **monthly report**, what are the reasons for the discrepancy?	1. Data entry errors<br>2. Arithmetic errors<br>3. Information from all source documents not compiled correctly<br>4. Data burden (too much data elements to be reported)<br>5. Illegible writing on the source document (not readable)<br>6. Lack of emphasis for data accuracy<br>7. Other (specify) ________________________
**8**	If there was a discrepancy observed between the **source document** and the **monthly report**, what are the reasons for the discrepancy?	1. Data entry errors<br>2. Arithmetic errors<br>3. Information from all source documents not compiled correctly<br>4. Data burden (too much data elements to be reported)<br>5. Illegible writing on the source document (not readable)<br>6. Lack of emphasis for data accuracy<br>7. Other (specify) ________________________
