# Supplementary material for: Exploring data quality and use of the routine health information system in Ethiopia: a mixed-methods study
Source: BMJ Open. 2021 Dec 23;11(12):e050356. doi: 10.1136/bmjopen-2021-050356 (PMC8710857; doi:10.1136/bmjopen-2021-050356)
Supplement: Supplementary data [file bmjopen-2021-050356supp003.pdf]

Data collection tool for Maternal Health Indicators

Checklists for collecting data on quality at woreda and health facility level, adapted from USAID RDQA Measure Evaluation

This questionnaire will be used to collect data from the health facilities and woreda health offices in order to assess the data quality and data management system.

Facility Identification

Interviewer Name: ____________________________________

Full reference of report (name, year, publisher etc.)			
Type of Document			
Study Design			
Study area/s			
Study period			
Study population			
Sample size			
Indicators	Definition (Numerator /Denominator)	Reported Coverage	
		National	Benishangul Gumz Region
Antenatal care visits at least one (ANC1)			
Antenatal care 4th visit (ANC4)			
Skilled delivery attendance			
Postnatal Care			
Comments			

Number	Question	Result	
SECTION 1: COVER PAGE			
Date	DAY______________<br>Day	MONTH____________<br>Month	YEAR_________<br>Year
FACILITY IDENTIFICATION			
Q001	Region Name		
Q002	Zone/sub-city name		
Q003	Facility ID		
Q004	Official name of facility		

Number	Question	Result	
Q005	Woreda Name		
Q006	Town Name		
Q007	Kebele Name		
Q008	Type of facility	HEALTH CENTRE<br>HEALTH POST	1<br>2
Q009	Urban/Rural	URBAN<br>RURAL	1<br>2

1. Service Delivery Sites (health facilities)

Demographic data			
	Information	Response Yes=1,No=2	Remark
1	Does the facility have the catchment population the calendar year?		If *'yes'* how much …
2	Does the health center know the total number of eligible mothers for maternal service to be provided in the calendar year?		
3	Is the denominator value (for ANC, delivery and PNC) found in the health center level the same as the one found in the national level		

A - Documentation Review:						
Review availability and completeness of all indicator source documents for the selected reporting period *(if all necessary documents/facility reports are available 1=Yes, if all the necessary documents/facility reports are not available, 2= No otherwise the answer will be 3=Yes partly*			ANC1	ANC4	SBA	EPNC
SDS_1A	Review available data sources for the reporting period being verified. Are all necessary data sources (registers tally and reports) available for review?					
	Tikmit 2010/Octo 2017	Tally				
		Register				
		Report from the lower level				
	Hidar 2010/Nov 2017	Tally				
		Register				
		Report from the lower level				
	Tahisas 2010/ Dec 2017	Tally				
		Register				
		Report from the lower level				
SDS_2A	Are all available data sources complete? (Register and/or tally sheet for 2nd quarter of 2010 E.C)					

	Tikmit 2010/Octo 2017	Tally				
		Register				
		Report from the lower level				
	Hidar 2010/Nov 2017	Tally				
		Register				
		Report from the lower level				
	Tahisas 2010/ Dec 2017	Tally				
		Register				
		Report from the lower level				
SDS_3A	Review the dates on the data sources. Do all dates fall within the 2nd quarter of 2010E.C? (*Yes=1, No=2*)					
	Tikmit 2010/October 2017					
	Hidar 2010/Nov 2017					
	Tahisas 2010/ Dec 2017					
SDS_4A	If the source document (service delivery register) is not available, what are the possible reasons?					
	1. Storage or archiving problems					
	2. Stock out of source document					
	3. Absence of designated staff					
	4. Other (specify)					
SDS_5A	If the source document (service delivery register) is not completely filled in, what are the possible reasons for the missing data?					
	1. Staffing issue(s) (shortage, absence)					
	2. Not understanding the data element					
	3. Presence of other vertical reporting requirement					
	4. Data burden (too much data elements to be recorded)					
	5. The recording tool is not designed as user friendly					
	6. other (specify):					

B. REPORT TIMELINESS				
SDS_1B	Does the health facility record the dates of submission of monthly HMIS reports to the Health center/Woreda (see logbook/computer)?	1. Yes ☐	0. No	
SDS_2B	If '*Yes*' for SDS_1`B, review the records and check the dates of submission for the three review months			

	Tikmit 2010/Octo	Hidar 2010/Nov	Tahisas 2010/ Dec 2017

	Were the HMIS monthly reports submitted on time? *(Current practices is submission from 21th to 26th of the month)*	1. Yes ☐<br>0. No ☐	1. Yes ☐<br>0. No ☐	1. Yes ☐<br>0. No ☐

C- Recounting reported Results (accuracy)					
Recount results from source documents, compare the verified numbers to the site reported numbers and explain discrepancies(if any)		ANC 1	ANC4	SBA	EPNC
SDS_1C	Recount the number of people, cases or events during the reporting period by reviewing the data source (register HC)/tally HP) (A).				
	Tikmit 2010/October 2017				
	Hidar 2010/Nov 2017				
	Tahisas 2010/ Dec 2017				
SDS_2C	Enter number of people, cases or events reported by the site during reporting period from site summary report(B)				
	Tikmit 2010/October 2017				
	Hidar 2010/Nov 2017				
	Tahisas 2010/ Dec 2017				
SDS_4C	Calculate the ratio of recounted to reported (A/B)				
	Tikmit 2010/October 2017				
	Hidar 2010/Nov 2017				
	Tahisas 2010/ Dec 2017				
SDS_5C	What are the reasons for discrepancy (if any) observed ((i.e. data entry errors, missing data sources, others)?				
	1. Storage or archiving problems				
	2. Absence of designated staff				
	3. Stock out of source document				
	4. other(specify)				

D. Systems Assessment		
I - M&E Structure, Functions and Capabilities (1.Yes completely 2. Partly 3. No 4.NA		
SDS_1D	Is there a designated staff responsible for reviewing aggregated numbers prior to submission to the next level? (e.g. to health center or wereda)	
SDS_2D	Have all relevant staff has received training on the data management processes and tools.	
The M&E unit has provided written guidelines to each sub-reporting level on (*Yes=1, No=2) NB: Ask and observe the available guidelines*		
SDS-3D	What they are supposed to report on?	
SDS_4D	How (e.g. in what specific format) reports are to be submitted?	
SDS_5D	To whom the reports should be submitted?	
SDS_6D	When the reports are due?	
III - Data-collection and Reporting Forms and Tools (Yes=1, No=2)		
SDS_7D	Have there been clear instruction/orientation given to relevant staffs on how to complete the data collection and reporting forms/tools.	
SDS_8D	Is the supply of standard reporting forms/tools consistent in the facility?	
SDS_9D	Do the M&E Unit monitor on the consistent utilization of standard reporting forms/tools at all reporting levels.	
SDS_10D	The standard forms/tools are consistently used by the Service Delivery Site.	
SDS_11D	Are all *source documents* and *reporting forms* relevant for measuring the indicator (s) are available (hard copy print outs) for auditing purposes. *(Including dated print-outs in case of computerized system)*	
IV- Data Management Processes *(Yes=1, No=2) This section is only for Health Centers*		
SDS_12D	If applicable, there are quality controls in place for when data from paper-based forms are entered into a computer (e.g. double entry, post-data entry verification, etc). *(if not computerized skip to SDS_15D)*	
SDS_13D	If applicable, there is a written back-up procedure for when data entry or data processing is computerized.	
SDS_14D	*….if 'yes' for SDS_12D* the latest date of back-up is appropriate given the frequency of update of the computerized system (e.g., back-ups are weekly or monthly).	
SDS_15D	The recording and reporting system avoids double counting people *within* and *across* Service Delivery Points (e.g., a person receiving the same service twice in a reporting period, a person registered as receiving the same service in two different locations, etc).	

V - Links with National Reporting System *(Yes=1, No=2)*		
SDS_16D	The relevant national forms/tools are used for data-collection and reporting.	
SDS_17D	Data are reported through a single channel of the national information systems.	
VI – Use of Data for decision making *(Yes=1, No=2)*		
SDS_18D	The service delivery site develops charts, graphs, maps, etc …(If yes, ask to see them)	
SDS_19D	Staff at the health facility has access to guideline/technical assistance on data use (e.g. peer review meetings or during supervisory visits)	
SDS_20D	The analyzed data/results are presented/ disseminated to stakeholders in a timely manner so that the information can be used for informed decisions. (observe examples)	
SDS_21D	There are programmatic decision taken by the facility based on the analyzed data (see/observe examples)	

2. Woreda level assessment Questions

Demographic data			
	Information	Response Yes=1,No=2	Remark
1	Does the woreda health office have the catchment population the calendar year?		If *'yes'* how much …
2	Does the woreda health office know the total number of eligible mothers for maternal service to be provided in the calendar year?		
3	Is the denominator value (for ANC, delivery and PNC) found in the woreda the same as the one found in the national level?<br>(*If not the same explain the difference)*		

A. Recounting reported results (accuracy)					
Recount results from the periodic reports sent from service sites to the Woreda and compare to the value reported by the District. Explain discrepancies (if any)		ANC1	ANC4	SBA	Early PNC
WLA_1A	Re-aggregate the numbers from the reports received from all Service Delivery Sites. What is the re-aggregated number? [A]				
WLA_1A_a	Tikmit 2010 E.C / Oct. 2017				

WLA_1A_b	Hidar 2010 E.C / Oct. 2017				
WLA_1A_c	Tahisas 2010 E.C / Oct. 2017				
WLA_2A	What aggregated result was contained in the summary report prepared by the Woreda (and submitted to the next reporting level)? [B]				
WLA_2A_a	Tikmit 2010 E.C / Oct. 2017				
WLA_2A_b	Hidar 2010 E.C / Oct. 2017				
WLA_2A_c	Tahisas 2010 E.C / Oct. 2017				
WLA_3A	Calculate the ratio of recounted to reported results. [A/B]				
WLA_3A_a	Tikmit 2010 E.C / Oct. 2017				
WLA_3A_b	Hidar 2010 E.C / Oct. 2017				
WLA_3A_c	Tahisas 2010 E.C / Oct. 2017				
WLA_4A	What are the reasons for the discrepancy (if any) observed<br>1. data entry errors,<br>2. arithmetic errors,<br>3. missing data source,<br>4. (Other please specify)?				

B. Report performance

	Report completeness	
WLA_5A	How many facilities were expected to report [A]	
	Tikmit 2010 E.C / Oct. 2017	
	Hidar 2010 E.C / Oct. 2017	
	Tahisas 2010 E.C / Oct. 2017	
WLA_6A	How many facilities actually reported [B]	
	Tikmit 2010 E.C / Oct. 2017	
	Hidar 2010 E.C / Oct. 2017	
	Tahisas 2010 E.C / Oct. 2017	
WLA_7A	Calculate % Available Reports [B/A] sum	
	Tikmit 2010 E.C / Oct. 2017	
	Hidar 2010 E.C / Oct. 2017	
	Tahisas 2010 E.C / Oct. 2017	
WLA_8A	Check the dates on the reports received. How many reports were received on time? (i.e., received by the due date). [C]	

WLA_9A	Calculate % On time Reports [C/B]				
WLA_10A	How many reports were complete? (i.e., complete means that the report contained all the required indicator data, the date of reception of the report by the District, and the authentication by the responsible staff). [D]				
WLA_11A	Calculate % Complete Reports [D/B]				
WLA_12A	If any monthly HMIS reports were not complete, what are the possible reasons for the missing data? *(Multiple responses is possible)* *1.* Staffing issue(s) (e.g. staff shortage, absence of designated staff, etc) 2. Not understanding the data element(s) 3.Presence of other vertical reporting requirements 4.Data burden (too much data elements to be recorded) 5.The design of the reporting form is not user friendly 6.Other (specify)				
	Report timeliness				
WLA_13A	Does the Woreda office record receipt dates of monthly HMIS reports (observe logbook/electronic system)?	1. Yes ☐	0. No ☐	If No, skip to WLA_15A	
WLA_14A	If WLA_13A is 'yes', check the receipt dates for the three review months. How many reports were received on or before the 26th of the month	Health facility type	Tikmit 2010/Oct.2 017	Hidar 2010/Nov. 2017	Tahisas 2010 / Dec.2017
		Health centre			
		Health post			
WLA_15A	Does the Woreda office keep a record of its submission of monthly aggregated HMIS reports to Zonal or regional offices (e.g. emails, stamps, receipts, log book, etc.)?	1. Yes ☐	0. No ☐		
WLA_16A	If WLA_16A is yes, check the submission dates (date from---) of the aggregate HMIS reports for the three review months.	Tikimt 2010/Oct 2017 1. Yes ☐0. No ☐	Hidar 2010/Nov.2 0171. Yes ☐0. No ☐	Tahisas 2010 / Dec.2017 1. Yes. 0. No	
	Part 2. Systems Assessment (Woreda)				
I - M&E Structure, Functions and Capabilities *(1. Yes completely 2. Yes partly 3. No 4. NA)*					
SAW_1A	Are there designated staffs responsible for reviewing the quality of data (i.e., accuracy, completeness and timeliness) received from sub-reporting levels (e.g., service points)?				

SAW_2A	Are there designated staff responsible for reviewing aggregated numbers prior to submission to the next level (e.g., to the central M&E Unit).	
SAW_3A	Does all relevant staff have received training on the data management processes and tools?	
II DATA QUALITY ASSESSMENT MECHANISMS		
SAW_4	Has the Woreda conducted data quality assessments at all health facilities in the review three months? (Please observe)	0. No RDQA conducted<br>1. Yes, RDQA has been conducted in all health posts, Health centers, and hospitals<br>2. RDQA has been conducted, but only in some facilities
SAW_5	Does the Woreda use data quality assessment tools (e.g., RDQA/data verification, in-built electronic data quality validation rules/system)? (Please observe)	0. No ☐<br>1. Yes, observed ☐<br>2. Yes, not observed
SAW_6	Does the Woreda maintain a record of health facility data quality assessments conducted in the past three months? (Please observe)	0. No ☐<br>1. Yes, observed ☐<br>2. Yes, not observed
SAW_7	Does the Woreda maintain records of feedback to health facilities on data quality assessment findings? (Please observe)	0. No ☐<br>1. Yes, observed ☐<br>2. Yes, not observed
III- Indicator Definitions and Reporting Guidelines		
	The M&E Unit has provided written guidelines to each sub-reporting level on:- *(Yes=1, N0=2)*	
WID_1	What they are supposed to report on.	
WID_2	How (e.g., in what specific format) reports are to be submitted.	
WID_3	To whom the reports should be submitted.	
WID_4	To When the reports are due.	
IV - Data-collection and Reporting Forms / Tools *(Yes=1, No=2)*		
WDC_1	Clear instructions have been provided by the M&E Unit on how to complete the data collection and reporting forms/tools.	
WDC_2	The M&E Unit has identified standard reporting forms/tools to be used by all reporting levels	
WDC_3	The standard forms/tools are consistently used by the Service Delivery Site.	
WDC_4	All source documents and reporting forms relevant for measuring the indicator(s) are available (printed out backups) for auditing purposes (including dated print-outs in case of computerized system).	
V- Data Management Processes *(Yes=1, No=2)*		

WDM_1	Feedback is systematically provided to all service points on the quality of their reporting (i.e., accuracy, completeness and timeliness).	
WDM_2	Are there quality controls in place for when data from paper-based forms are entered into a computer (e.g., double entry, post-data entry verification, etc)?	
WDM_3	Is there a written back-up procedure for when data entry or data processing is computerized?	
WDM_4	If WDM_3 'yes', the latest date of back-up is appropriate given the frequency of update of the computerized system (e.g., back-ups are weekly or monthly).	
WDM_5	There is a written procedure to address late, incomplete, inaccurate and missing reports; including following-up with health facility on data quality issues.	
VI - Links with National Reporting System *(Yes=1, No=2)*		
WDM_6	The data are reported through a single channel of the national reporting system.	
WDM_7	The relevant national forms/tools are used for data-collection and reporting.	
VII – Use of Data for decision making *(Yes=1, No=2)*		
WDU_1D	The wereda develops charts, graphs, maps, etc …*(If yes, ask to see them)*	
WDU_2D	Staff at the wereda has access to guideline/technical assistance on data use (e.g. peer review meetings or during supervisory visits)	
WDU_3D	The analyzed data/results are presented/ disseminated to stakeholders in a timely manner so that the information can be used for informed decisions. (observe examples)	
SDS_31D	There are programmatic decision taken by the wereda based on the analyzed data *(see/observe examples of document)*	
