# Supplementary material for: Exploring data quality and use of the routine health information system in Ethiopia: a mixed-methods study
Source: BMJ Open. 2021 Dec 23;11(12):e050356. doi: 10.1136/bmjopen-2021-050356 (PMC8710857; doi:10.1136/bmjopen-2021-050356)
Supplement: Supplementary data [file bmjopen-2021-050356supp004.pdf]

Data quality assessment tool for nuitrition indicators

Data quality assessment tools for *health center*

**1. Health center Checklist**		
**Person Interviewed (title)**		
**Health center Name**		
(Interviewer: Please verify if the following equipment is available in the Health center*)*		
**1. Equipment**		
1.1. Computer (give a number)	1. yes	0. no
1.2. Data Back-up Unit (e.g. CD, flash disc)	1. yes	0. no
1.3. Printers	1. yes	0. no
1.4. UPS	1. yes	0. no
1.5. Generators	1. yes	0. no
1.6. Regular telephone	1. yes	0. no
1.7. Access to the internet	1. yes	0. no
1.8. Calculator	1. yes	0. no
**2. Utilities**		
2.1. Is there an electricity supply?	1. yes	0. no
2.2. How often is the electricity supply interrupted?<br>0. Never/occasionally 1. Once a month 2. Twice a month 3. Weekly 4. Daily		
2.3. Is the room, where the computer hardware is kept, air-conditioned?	1. yes	0. no
**3. Availability of registers, forms**		
Type of record, report or register	Have you run out of this form in the past 6 month? If so, why?	
3.1 Integrated Management of Neonatal and Childhood Illnesses (IMNCI) register	1. yes<br>**Reasons:**	0. no
3.2. Comprehensive and Integrated Nutrition Service for <5 years children tally sheet	1. yes<br>**Reasons:**	0. no
3.3 Outpatient Therapeutic Program (OTP) card	1. yes<br>**Reasons:**	0. no
3.4 Stabilization Centre (SC) register	1. yes<br>**Reasons:**	0. no
3.5 Stabilization Centre (SC) tally sheet	1. yes<br>**Reasons:**	0. no
**4. Trained staff**		
Are there staff members who received any training in the recording, processing, or reporting of health information during the last two years? If yes;		
**4.1. HMIS**		
4.1.1. Health officer	1. yes	0. no
4.1.2. Nurse	1. yes	0. no
4.1.3. Health information technician (HIT)	1. yes	0. no
4.1.4. Other (specify)		
**4.2. CHIS**		
4.2.1. Health officer	1. yes	0. no
4.2.2. Nurse	1. yes	0. no
4.2.3. Health information technician (HIT)	1. yes	0. no
4.2.4. Other (specify)		
**4.3. DHIS2**		

4.3.1. Health officer	1. yes	0. no
4.3.2. Nurse	1. yes	0. no
4.3.3. Health information technician (HIT)	1. yes	0. no
4.3.4. Other (specify)		

Technical factors- Health center

18	Does a tally sheet exist?	1. Yes, Observed<br>0. No
19	Do data processing procedures exist?	1. Yes, Observed<br>0. No
20	Does the facility produce the following?	
20.1	Calculate nutrition indicators of the facility	1. Yes, Observed<br>0. No
20.2	Comparisons with Woreda targets	1. Yes, Observed<br>0. No
20.3	Comparisons with National targets	1. Yes, Observed<br>0. No
20.4	Comparisons among types of nutrition services coverage	1. Yes, Observed<br>0. No
20.5	Comparisons of nutrition services data over time (monitoring over time)	1. Yes, Observed<br>0. No
21	Does a procedure manual for nutrition services data collection (with definitions) exist?	1.Yes, Observed<br>0. No

**Data Completeness- at Health center level for the last six months**							
22	What is the number of Health posts in the catchment area that are supposed to report nutrition indicators?						
23	What is the number of Health posts in the catchment area that are actually reporting nutrition indicators						
24	How many nutrition data indicator is the health center supposed to receive on the HMIS monthly report?						
25	How many nutrition data indicator is the health center receive on the HMIS monthly report?						
26	How many nutrition indicators does the health center need to report on in the HMIS monthly report?						
27	Does the health center keep copies of HMIS monthly reports sent by health posts?	1. Yes<br>0. No					
28	Does the health center keep copies of HMIS monthly reports sent to the woreda health office?	1. Yes<br>0. No					
29	Count the number of monthly reports submitted by the health posts for the last 6 months	Month 1	Month 2	Month 3	Month 4	Month 5	Month 6
30		Month 1	Month 2	Month 3	Month 4	Month 5	Month 6

, et al. BMJ Open 2021; 11:e050356. doi: 10.1136/bmjopen-2021-050356

	Count the number of monthly reports submitted to the woreda health office by the health center for the last 6 months						
31	Does the health center fill the monthly report form completely	1.Yes, Observed<br>0. No					
32	Count the number of nutrition indicators that are supposed to be filled in by this facility but left blank without indicating "0" for the last 6 months reports						

**Data timeliness- at Health center level for the last six months**							
33	When are you expecting to receive the HMIS report from health posts?						
34	Does the health center record receipt dates of the HMIS monthly report?	1.Yes	0.No				
	If yes, check the dates of receipts for the last six months						
			HP 1	HP 2	HP 3	HP 4	HP 5
34.1	Month (specify)	1.Before deadline<br>0. After deadline					
34.2	Month (specify)	1.Before deadline<br>0. After deadline					
34.3	Month (specify)	1.Before deadline<br>0. After deadline					
34.4	Month (specify)	1.Before deadline<br>0. After deadline					
34.5	Month (specify)	1.Before deadline<br>0. After deadline					
34.6	Month (specify)	1.Before deadline<br>0. After deadline					
35	When are you expected to submit HMIS report to woreda health office?						
36	Does the health center have a record of submitting data on time to woreda level?	1.Yes<br>0.No					
	If yes, check the dates of submission for the last six months						
36.1	Month (specify)	1.Before deadline<br>0. After deadline					
36.2	Month (specify)	1.Before deadline<br>0. After deadline					
36.3	Month (specify)	1.Before deadline<br>0. After deadline					
36.4	Month (specify)	1.Before deadline<br>0. After deadline					
36.5	Month (specify)	1.Before deadline<br>0. After deadline					
36.6	Month (specify)	1.Before deadline<br>0. After deadline					

, *et al. BMJ Open* 2021; 11:e050356. doi: 10.1136/bmjopen-2021-050356

Data Accuracy Check- health center level for the last six months<br>Cluster report from all the health posts									
37	Manually count the number of following data items from the HMIS monthly reports for the last 6 months. Compare the figures with the reports from the computer or paper database.								
		Indicator							
	Denominator								
	Month (specify)	Number of <5 children received VAS		Number of <5 children With SAM		Total number of children received GMP (< 2 years)		Number of children with two doses of Deworming (2-5 years)	
		HMIS report received from health post aggregated	HMIS report send to woreda health office	HMIS report received from health post aggregated	HMIS report send to woreda health office	HMIS report received from health post aggregated	HMIS report send to woreda health office	HMIS report received from health post aggregated	HMIS report send to woreda health office
37.1									
37.2									
37.3									
37.4									
37.5									
37.6									

Data Accuracy Check- health center level for the last six months Name of health post …………………………………									
38	Manually count the number of following data items from the HMIS monthly reports for the last 6 months. Compare the figures with the reports from the computer or paper database.								
		Indicator							
	Denominator								
	Month (specify)	Number of <5 children received VAS		Number of <5 children With SAM		Total number of children received GMP (< 2 years)		Number of children with two doses of Deworming (2-5 years)	
		HMIS report received from health post	HMIS report send to woreda health office	HMIS report received from health post	HMIS report send to woreda health office	HMIS report received from health post	HMIS report send to woreda health office	HMIS report received from health post	HMIS report send to woreda health office
38.1									
38.2									

38.3									
38.4									
38.5									
38.6									
