# Supplementary material for: Exploring data quality and use of the routine health information system in Ethiopia: a mixed-methods study
Source: BMJ Open. 2021 Dec 23;11(12):e050356. doi: 10.1136/bmjopen-2021-050356 (PMC8710857; doi:10.1136/bmjopen-2021-050356)
Supplement: Supplementary data [file bmjopen-2021-050356supp005.pdf]

Table 1 Coding framework

PRISM themes	Themes from ORCA	Codes
**Technical factors**	Denominator	• Wrong denominator<br>• Outdated denominator
	Complexity of forms and procedures for data recording and reporting	• Lack of guideline for recording<br>• Complexity of Indicator definitions<br>• Multiple forms<br>• Parallel reporting
	Language issues;	• Language barrier
**Behavioral Factors**	Knowledge and skills to manage HMIS	• understanding of health data<br>• Experience on data collection and reporting<br>• Lack of program knowledge<br>• Poor capacity on recording<br>• Low training<br>• Lack of skill operating computers
	Indicator definitions	• Knowledge on Health data and indicator definition
	motivation	• motivation
	Perception towards data quality and the level of emphasis given	• Perception towards data quality and the level of emphasis given
	Available interventions to improve Data quality and use,	• Available interventions to improve Data quality and use,
	Data use	• Data use
**Organizational Factors**	Communication between levels;	• Communication platform between the hierarchy<br>• Communication between departments<br>• HMIS Communication and feedback<br>• communication among HMIS, logistic and nutrition focal
	Monitoring,	• Routine data quality assessment<br>• Performance monitoring team
	supervision and evaluation	• feedback and learning<br>• HMIS data quality assurance methods<br>• Report evaluation practice
	Accountability	• Incentive mechanism
	Training: inadequate training,	• Capacity buildings<br>• Trained man power<br>• training for a wrong person
	Staff turnover,	• staff Turnover
	Staff retention	• attrition<br>• Staff rotation<br>• lack of HR resource
	Workload	• Workload

**Availability of resources**	Supply and availability of equipment for HMIS activity	• Shortage of HMIS tools
	Availability of source documents;	• HMIS budget, logistics and infrastructure<br>• transportation logistics
	Availability of computers and electricity	• availability of printing materials<br>• availability of computer<br>• availability of electricity
	Human resource for HMIS (shortage in number),	• Shortage of human resource for HMIS
	finance	• Finance
**RHIS Processes**	Data flow	• Data flow/Difference
	Data recording and reporting practice	• Wrong reporting/over reporting/under reporting
	Triangulation of data within routine health information system	• Different Reporting period<br>• Separate Logistics and TB Service reporting
	Completeness,	• double reporting during referral
	timeliness,	• Delay in reporting
	accuracy of reporting	• Source of data<br>• Reporting format difference (HC and HP)
		• Under report for private sector
