# Supplementary material for: Exploring data quality and use of the routine health information system in Ethiopia: a mixed-methods study
Source: BMJ Open. 2021 Dec 23;11(12):e050356. doi: 10.1136/bmjopen-2021-050356 (PMC8710857; doi:10.1136/bmjopen-2021-050356)
Supplement: Author's manuscript [file bmjopen-2021-050356.draft_revisions.pdf]

BMJ Open

BMJ Open is committed to open peer review. As part of this commitment we make the peer review history of every article we publish publicly available.

When an article is published we post the peer reviewers' comments and the authors' responses online. We also post the versions of the paper that were used during peer review. These are the versions that the peer review comments apply to.

The versions of the paper that follow are the versions that were submitted during the peer review process. They are not the versions of record or the final published versions. They should not be cited or distributed as the published version of this manuscript.

BMJ Open is an open access journal and the full, final, typeset and author-corrected version of record of the manuscript is available on our site with no access controls, subscription charges or pay-per-view fees (http://bmjopen.bmj.com).

If you have any questions on BMJ Open's open peer review process please email info.bmjopen@bmj.com

BMJ Open

Exploring data quality and use of the routine health information system in Ethiopia: a mixed-methods study

Journal:	*BMJ Open*
Manuscript ID	bmjopen-2021-050356
Article Type:	Original research
Date Submitted by the Author:	17-Feb-2021

Complete List of Authors:	Adane, Abyot; Ethiopian Pharmaceutical Supply Agency<br>Adege, Tewabe M.; Ministry of Health<br>Ahmed, Mesoud M.; Ministry of Health<br>Anteneh, Habtamu A.; Ministry of Health<br>Ayalew, Emiamrew S.; Ministry of Health<br>Berhanu, Della; London School of Hygiene & Tropical Medicine<br>Berhanu, Netsanet; Ministry of Health<br>Getnet, Misrak; Ethiopian Public Health Institute<br>Bishaw, Tesfahun; Ministry of Health<br>Busza, Joanna; London School of Hygiene & Tropical Medicine<br>Cherinet, Eshetu; Ministry of Health<br>Dereje, Mamo; Ministry of Health<br>Desta, Tsega H.; Ministry of Health<br>Dibabe, Abera; Ministry of Health<br>Firew, Heven S.; Ethiopian Public Health Institute<br>Gebrehiwot, Freweini; Ethiopian Public Health Institute<br>Gebreyohannes, Etenesh; Ministry of Health<br>Gella, Zenebech; Ministry of Health<br>Girma, Addis; Ministry of Health<br>Halefom, Zuriash; Ministry of Health<br>Jama, Sorsa F.; Ministry of Health<br>Janson, Annika; London School of Hygiene & Tropical Medicine<br>Kemal, Binyam; Ministry of Health<br>Kiflom, Abiy; Ethiopian Pharmaceutical Supply Agency<br>Mazengiya, Yidnekachew D.; Ministry of Health<br>Mekete, Kalkidan; Ethiopian Public Health Institute<br>Mengesha, Magdelawit; Ministry of Health<br>Nega, Meresha W.; Ministry of Health<br>Otoro, Israel A.; Ministry of Health<br>Schellenberg, Joanna; London School of Hygiene & Tropical Medicine<br>Taddele, Tefera; Ethiopian Public Health Institute<br>Tefera, Gulilat; Ethiopian Pharmaceutical Supply Agency<br>Teketel, Admasu; Ethiopian Pharmaceutical Supply Agency<br>Tesfaye, Miraf; Ministry of Health<br>Tsegaye, Tsion; Ethiopian Pharmaceutical Supply Agency<br>Woldesenbet, Kidist; Ministry of Health<br>Wondarad, Yakob; Ministry of Health<br>Yusuf, Zemzem M.; Ministry of Health<br>Zealiyas , Kidist; Ethiopian Public Health Institute<br>Zeweli, Mebratom H.; Ministry of Health<br>Persson, Lars; London School of Hygiene & Tropical Medicine

	Lemma, Seblewengel ; London School of Hygiene & Tropical Medicine
Keywords:	PUBLIC HEALTH, HEALTH SERVICES ADMINISTRATION & MANAGEMENT, Health informatics < BIOTECHNOLOGY & BIOINFORMATICS

SCHOLARONE™ Manuscripts

I, the Submitting Author has the right to grant and does grant on behalf of all authors of the Work (as defined in the below author licence), an exclusive licence and/or a non-exclusive licence for contributions from authors who are: i) UK Crown employees; ii) where BMJ has agreed a CC-BY licence shall apply, and/or iii) in accordance with the terms applicable for US Federal Government officers or employees acting as part of their official duties; on a worldwide, perpetual, irrevocable, royalty-free basis to BMJ Publishing Group Ltd ("BMJ") its licensees and where the relevant Journal is co-owned by BMJ to the co-owners of the Journal, to publish the Work in this journal and any other BMJ products and to exploit all rights, as set out in our licence.

The Submitting Author accepts and understands that any supply made under these terms is made by BMJ to the Submitting Author unless you are acting as an employee on behalf of your employer or a postgraduate student of an affiliated institution which is paying any applicable article publishing charge ("APC") for Open Access articles. Where the Submitting Author wishes to make the Work available on an Open Access basis (and intends to pay the relevant APC), the terms of reuse of such Open Access shall be governed by a Creative Commons licence – details of these licences and which Creative Commons licence will apply to this Work are set out in our licence referred to above.

Other than as permitted in any relevant BMJ Author's Self Archiving Policies, I confirm this Work has not been accepted for publication elsewhere, is not being considered for publication elsewhere and does not duplicate material already published. I confirm all authors consent to publication of this Work and authorise the granting of this licence.

For peer review only

Exploring data quality and use of the routine health information system in Ethiopia: a mixed-methods study

The Operational Research and Coaching for Analysts (ORCA)-team

Abyot Adane,[1] Tewabe M. Adege,[2] Mesoud M. Ahmed,[2] Habtamu A. Anteneh,[2] Emiamrew S. Ayalew,[2] Della Berhanu,[3] Netsanet Berhanu,[2] Misrak G. Beyene,[4] Tesfahun Bishaw,[2] Joanna Busza,[3] Eshetu Cherinet,[2] Mamo Dereje,[2] Tsega H. Desta,[2] Abera Dibabe,[2] Heven S. Firew,[4] Freweini Gebrehiwot,[4] Etenesh Gebreyohannes,[2] Zenebech Gella,[2] Addis Girma,[2] Zuriash Halefom,[2] Sorsa F. Jama,[2] Annika Janson,[3,6] Binyam Kemal,[2] Abiy Kiflom,[1] Yidnekachew D. Mazengiya,[2] Kalkidan Mekete,[4] Magdelawit Mengesha,[2] Meresha W. Nega,[2], Israel A. Otoro,[2] Joanna Schellenberg,[3] Tefera Taddele,[4] Gulilat Tefera,[1] Admasu Teketel,[1] Miraf Tesfaye,[2] Tsion Tsegaye,[1] Kidist Woldesenbet,[2] Yakob Wondarad,[2] Zemzem M. Yusuf,[2] Kidist Zealiyas,[4] Mebratom H. Zeweli,[2] Lars Åke Persson,[3] Seblewengel Lemma[3]

[1] Ethiopian Pharmaceutical Supply Agency, Addis Ababa, Ethiopia

[2] Ministry of Health, Addis Ababa, Ethiopia

[3] London School of Hygiene & Tropical Medicine, London, United Kingdom

[4] Ethiopian Public Health Institute, Addis Ababa, Ethiopia

[5] Department of Women´s and Children's Health, Karolinska Institutet, Stockholm, Sweden

Correspondence to: Dr Seblewengel Lemma

London School of Hygiene & Tropical Medicine, Based at the Ethiopian Public Health Institute

Addis Ababa, Ethiopia

Seblewengel.Abreham@lshtm.ac.uk;

Keywords:

Completeness, Data quality, Health Management Information System, Interviews, Perceptions, Performance of Routine Information System Management, Routine Health Information System, WHO Data Quality Review toolkit

For peer review only

For peer review only - http://bmjopen.bmj.com/site/about/guidelines.xhtml

For peer review only

For peer review only - http://bmjopen.bmj.com/site/about/guidelines.xhtml

e-mail addresses:

Abyot Adane: abyot.andu@gmail.com

Tewabe Manaye Adege: tewabemanaye@gmail.com

Mesoud Mohammed Ahmed: Mesoud.mohammed@gmail.com

Habtamu Alemay Anteneh: alemayhabtamu@gmail.com

Emiamrew Sisay Ayalew: emiamrew.sisay@moh.gov.et

Della Berhanu: della.berhanu@lshtm.ac.uk

Netsanet Berhanu: netsanet.berhanu@moh.gov.et

Misrak Getnet Beyene: misrakg81@gmail.com

Tesfahun Bishaw: bishawtesfahun@gmail.com

Joanna Busza: joanna.busza@lshtm.ac.uk

Eshetu Cherinet: eshecher2005@gmail.com

Mamo Dereje: mamodereje355@gmail.com

Tsega Hailu Desta: tsehame@gmail.com

Abera Dibabe: aberad968@gmail.com

Heven Sime Firew: hevensime@yahoo.com

Freweini Gebrehiwot: fre.geb2012@gmail.com

Etenesh Gebreyohannes: mhexpert3.mh@gmail.com

Zenebech Gella: gellazeniti@gmail.com

Addis Girma: addisgirma2016@gmail.com

Zuriash Halefom: zuriash.halefom@gmail.com

Sorsa Faltamo Jama: sorsa1982@gmail.com

Annika Janson: annika.janson@sll.se

Binyam Kemal: binyam.kemal@moh.gov.et

For peer review only - http://bmjopen.bmj.com/site/about/guidelines.xhtml

Abyi Kiflom: abiy.kiflom@gmail.com

Seblewengel Lemma: Seblewengel.Abreham@lshtm.ac.uk

Yidnekachew Degefaw Mazengiya: ydegefaw@gmail.com

Kalkidan Mekete: kalnyou@yahoo.com

Magdelawit Mengesha: magdelawit.mengesha@moh.gov.et

Meresha W. Nega: workumersha98@gmail.com

Israel Ataro Otoro: israelotoro1@gmail.com

Lars Åke Persson: lars.persson@lshtm.ac.uk

Joanna Schellenberg: joanna.schellenberg@lshtm.ac.uk

Kidist Zealiyas shita: kzealiyas@gmail.com

Tefera Taddele: tefetes@gmail.com

Gulilat Tefera: gulelatmam@gmail.com

Admasu Teketel: admasuteketel@yahoo.com

Miraf Tesfaye: tesfayemiraf@gmail.com

Tsion Tsegaye: tsi.destiny@gmail.com

Kidist Woldesenbet: kidistwoldesenbet@gmail.com

Yakob Wondarad: Yakobwondaradd@gmail.com

Zemzem Mohammed Yusuf: zemzem.mohammed@moh.gov.et

Mebratom Haile Zeweli: mebrahtom2007@gmail.com

Word count: Abstract 292/300; main text 4047/4000, six figures and tables

For peer review only

ORCID-ID:

Abyot Adane; 0000-0001-9831-3248

Tewabe Manaye Adege; 0000-0001-6894-9966

Mesoud Mohammed Ahmed; 0000-0002-4740-2272

Habtamu Alemay Anteneh; 0000-0003-3755-3357

Emiamrew Sisay Ayalew; 0000-0002-4857-5802

Della Berhanu; 0000-0002-4984-893X

Netsanet Brehanu; 0000-0002-3435-0741

Misrak Getnet Beyene; 0000-0002-6629-756X

Tesfahun Bishaw; 0000-0002-3149-8177

Joanna Busza; 0000-0001-6334-0318

Eshetu Cherinet; 0000-0003-2882-5998

Mamo Dereje; 0000-0002-6581-0923

Tsega Hailu Desta; 0000-0002-5246-9295

Abera Dibabe; 0000-0002-6269-1869

Zuriash Halefom; 0000-0002-6135-3489

Sorsa Faltamo Jama; 0000-0002-7637-3255

Heven Sime Firew; 0000-0002-2732-1635

Freweini Gebrehiwot; 0000-0002-6337-1576

Etenesh Gebreyohannes; 0000-0002-3956-0561

Zenebech Gella; 0000-0001-8881-0152

Addis Girma; 0000-0003-2800-2916

Annika Janson; 0000-0001-5106-5670

Binyam Kemal; 0000-0002-1307-7643

Abiy Kiflom; 0000-0001-9593-2799

Seblewengel Lemma; 0000-0001-5910-3723

Yidnekachew Degefaw Mazengiya; 0000-0002-0224-1890

Kalkidan Mekete; 0000-0002-1786-5161

Magdelawit Mengesha; 0000-0003-0523-1396

For peer review only

Meresha W. Nega; 0000-0001-8222-7572

Israel Ataro Otoro; 0000-0001-6533-080X

Lars Åke Persson; 0000-0003-0710-7954

Joanna Schellenberg; 0000-0002-0708-3676

Kidist Zealiyas shita; 0000-0001-5248-5036

Tefera Taddele; 0000-0002-2327-136x

Gulilat Tefera; 0000-0001-9927-1780

Admasu Teketel; 0000-0002-3337-838X

Tsion Tsegaye; 000-0003-4737-6059

Miraf Tesfaye; 0000-0003-2726-050X

Kidist Woldesenbet; 0000-0003-1016-6656

Yakob Wondarad; 0000-0001-7415-5767

Zemzem Mohammed Yusuf; 0000-0002-3827-9225

Mebratom Haile Zeweli; 0000-0002-7845-2790

For peer review only

For peer review only - http://bmjopen.bmj.com/site/about/guidelines.xhtml

Abstract (292/300)

Objective A routine health information system (RHIS) enables decision-making in the health care system. We aimed to analyse data quality at the district and regional level and explore factors and perceptions affecting the quality and use of routine data.

Design This was a mixed-methods study. We used the World Health Organization toolkit for analysing data quality and interviewed staff at the point of data generation and along with the flow of data. Data were analysed using the Performance of Routine Information System Management framework.

Setting This study was performed in eight districts in four regions of Ethiopia. The study was nested within a two-year programme of the Operational Research and Coaching for government Analysts.

Participants We visited 45 health posts, 1 district hospital, 16 health centres, and eight district offices for analysis of routine RHIS data and interviewed 117 staff members for the qualitative assessment.

Outcome measures We assessed availability of source documents, completeness, timeliness, and accuracy of reporting of routine data, and explored data quality and use perceptions.

Results There was variable quality of both indicator and data element. Data on maternal health and immunization were of higher quality than data on child nutrition. Issues ranged from simple organizational factors, such as availability of register books, to intricate technical issues, like complexity of indicators and choice of denominators based on population estimates. Respondents showed knowledge of the reporting procedures, but

For peer review only - http://bmjopen.bmj.com/site/about/guidelines.xhtml

also demonstrated limited skills, lack of supportive supervision, and reporting to please the next level. We saw limited examples of the use of data by the staff who were responsible for data reporting.

Conclusion We identified important organizational, technical, behavioural, and process factors that need further attention to improve the quality and use of routine health information system data in Ethiopia.

Strengths and limitations of this study

- We assessed data quality and explored perceptions around data quality and use across a range of health indicators
- Over 100 staff from different levels of Ethiopia's health system were interviewed and we attained thematic saturation.
- The qualitative findings suggested similar data quality problems as the quantitative results
- We conducted a member check test, confirming that our results were credible
- Our results from the quantitative data have limited generalizability, because we took a small sample size which was purposive rather than representative.

Background (377)

High-quality, real-time data on the burden of disease and performance of the health sector are critical for decision-making and resource allocation [1]. A routine Health Information System (RHIS) aggregates information across the health system [2–4]. Despite improvements, efforts to increase coverage, quality, equity, and accountability of health services are often hampered by the lack of reliable data [5–7].

For peer review only - http://bmjopen.bmj.com/site/about/guidelines.xhtml

For peer review only

The Ethiopian Ministry of Health named the *Information Revolution* as one of four agendas in its first Health Sector Transformation Plan [8], aiming to advance information collection, analysis, presentation, and dissemination. RHIS data are generated at the point of service delivery at primary level (health posts, health centres, primary hospitals), secondary level (general hospitals) and tertiary-level health care (specialised hospitals). The web-based open-source computer software District Health Information System was introduced in 2015 [9,10]. Data are forwarded and aggregated at district, regional and national administrative levels. However, the quality and use of RHIS data continues to be a challenge in Ethiopia [11–14] and elsewhere [15–17].

Factors affecting data quality can be classified as technical, behavioural and organizational according to the Performance of Routine Information System Management (PRISM) framework. Technical factors relate to the ease of data collection, collation, analysis and reporting while behavioural factors include individuals' knowledge, attitude and skills related to RHIS processes. Organizational factors focus on availing human capital, infrastructure and a functional control system [18]. These factors directly affect RHIS performance but also interact with each other, requiring an integrated approach to produce favourable outcomes [19]. Understanding how these factors function at national level using the PRISM conceptual framework is an appropriate way to identify and implement appropriate interventions.

The overall aim of this study was to analyse RHIS data quality and use at district and regional levels, and explore perceptions of factors affecting data quality through a mixed-methods approach. This paper brings together findings from the Operational Research and Coaching for Analysts (ORCA) work at district and regional level to contribute to understanding and

For peer review only - http://bmjopen.bmj.com/site/about/guidelines.xhtml

strengthening the RHIS across the whole health system. The specific objectives were to analyse the timeliness, completeness, and accuracy of reporting of RHIS data generated at primary health care level, and to explore reasons for problems in data quality and use along the flow of data.

Methods (671)

Study setting and design

The Ethiopian Ministry of Health (MOH) initiated the ORCA project in collaboration with the Ethiopian Public Health Institute (EPHI), the Ethiopian Pharmaceutical Supply Agency (EPSA) and London School of Hygiene & Tropical Medicine (LSHTM). ORCA was designed to guide participants through a research cycle that diagnosed and investigated the current state of data quality and use within the Ethiopian health system, taking into consideration key strategic health metrics. A group of 36 analysts from the MOH, EPHI, and EPSA participated alongside their normal work duties from June 2018 to June 2020. The ORCA participants chose to work in six thematic groups: Maternal Health, Neonatal Survival, Immunization, Child Nutrition, Malaria, and Tuberculosis

This was a mixed-methods study performed by the ORCA participants including quantitative analysis of district-level data, complemented by qualitative interviews with key informants at different levels. Fieldwork was conducted by each ORCA thematic group. Data were collected in eight districts in four regions in Ethiopia (Afar, Oromia, Southern Nations, Nationalities and People's region, and Tigray), selected in consultation with the regional health offices, from August to December 2019.

Sampling and recruitment

For peer review only - http://bmjopen.bmj.com/site/about/guidelines.xhtml

Health centres and health posts providing services for more than one year were included in the quantitative data collection. In each district, aggregated data were also assessed at district health offices. For the qualitative assessment, key informants were recruited purposively along the flow of data from health posts, health centres, district health offices, zonal health offices, regional health bureaus and the MOH. Informants had served for at least one year in their respective post, and could provide in-depth information about RHIS data. The informants' professional designations were health extension worker, head of health facility, RHIS focal person, head of district health office, and program expert at district, zonal, regional or federal level.

Data collection and processing

Each ORCA thematic group prepared a desk review checklist for relevant indicators, drawing on standard data quality assessment tools [20]. The checklists were pre-tested in similar settings. Data were collected at health facilities from primary source documents and entered into Microsoft Excel for analysis.

A qualitative topic guide was prepared in English by each thematic group and translated into local languages (Amharic, Oromiffaa, Tigrigna or Afar). Interview guides were pre-tested and refined, and further adapted during fieldwork to improve comprehensibility. Data collectors were ORCA team members trained in qualitative and mixed-methods research. Interviews lasted 30 to 60 minutes, recorded, and field notes were taken by group members. After data collection, group members reflected on their work and identified points for exploration during subsequent interviews. Recordings were transcribed verbatim. Ten percent of the transcripts were cross-checked with the audio for completeness and accuracy.

Quantitative information

For peer review only - http://bmjopen.bmj.com/site/about/guidelines.xhtml

All definitions were based on the WHO toolkit [20]; *Availability of source document and report* was presented as a percentage, i.e. facilities with records, divided by the total facility months investigated; *Completeness of reporting* indicated the percentage of monthly reports received by the next level; *Timeliness of reporting* covered the proportion sent on time; and *Accuracy of reporting* indicated the ratio of numbers recounted and classified as exact match, within the data quality range (0.9-1.1), over-reporting (<0.9), or under-reporting (>1.1). Results were categorized by type of indicators and presented as percentage of health facility months.

Qualitative data analysis

Each group conducted thematic content analysis. After reading the verbatim transcripts, all group members coded the same interview and agreed on a coding framework. The group members divided interviews among themselves for coding, and met regularly to add codes to capture emerging ideas. Groups categorized codes into broader thematic areas. Each group prepared a report on qualitative results that were shared across groups. The joint results from all six thematic groups were synthesised using the PRISM framework [18]. Regular discussions were held to reflect on similarities and differences across the data sets, check for outliers and contradictory findings, and agree on distribution of key themes within the simplified structure of the framework. Finally, the result was shared with seven respondents at MOH to check for credibility.

For peer review only - http://bmjopen.bmj.com/site/about/guidelines.xhtml

Results (w2174)

In total, 62 facilities and eight district health offices were visited for analysis of RHIS data and 117 key informants were interviewed (Table 1). Of all interviews, 35/117 (30%) were with health extension workers at health posts.

Table 1: Desk reviews and qualitative interviews conducted by ORCA thematic groups and other background information, Ethiopia, 2019/20

Characteristics	Desk review (n=70)	Qualitative interviews (n=117)
**Health facilities/ offices visited**		
Health Post	45 (64%)	35 (30%)
Health Centre	16 (23%)	33 (28%)
District Hospital	1 (1%)	2 (2%)
District Health Offices	8 (11%)	21 (18%)
Zonal health office	0 (0%)	1 (1%)
Regional health office	0 (0%)	17 (15%)
Federal ministry of Health	0 (0%)	8 (7%)
**Region**		
Tigray	5 (7%)	15 (13%)
Afar	19 (27%)	37 (32%)
Oromia	17 (24%)	25 (21%)
SNNPR	29 (41%)	32 (27%)
National	0 (0%)	8 (7%)
**Thematic group**		
Maternal health [1]	12 (17%)	18 (15%)
Neonatal Survival[2]	17 (24%)	14 (12%)
Immunization[3]	9 (13%)	12 (10%)
Child nutrition[4]	9 (13%)	25 (21%)
Malaria[5]	6 (9%)	17 (15%)
Tuberculosis[6]	17 (24%)	31 (27%)

[1] 1st antenatal care, 4th antenatal care, postnatal care & skilled delivery
[2] early institutional death (0-6 days), early community death (0-6 days), live birth in Kebele
[3] Pentavalent vaccine third dose, Measles, fully vaccinated
[4] Vitamin A supplementation, Deworming, Severe acute malnutrition, Growth monitoring promotion
[5] Suspected malaria, Positive malaria, All malaria
[6] New and relapse tuberculosis, & Treated tuberculosis

For peer review only - http://bmjopen.bmj.com/site/about/guidelines.xhtml

For peer review only

Availability of source documents, completeness, timeliness and accuracy of reporting

The availability of source document ranged from 55% to 100%. Only documents for skilled birth attendance reached 100% in observed health facilities (fig 1).

The majority of indicators had gaps in reporting. Maternal health and postnatal indicators had the most gaps in reporting. Completeness of reporting for nutrition was also low, at slightly over 50% for the facility-months reviewed. Completeness was much higher for immunization. Timeliness was over 90% for maternal health indicators, whereas just over half of reports for nutrition indicators were submitted on time (fig 2).

Maternal and immunization indicators had lower proportions of reports within the range for acceptable quality, whereas nutrition indicators were mainly reported within the quality range. Varying levels of over-reporting were observed in all service coverage indicators, but not for severe acute malnutrition. (fig 2 and 3).

Respondents' views on data processes and quality

Interview respondents reported that data generation and flow mostly occurred as intended. At health facilities, data were usually recorded by hand using standard on paper forms, while district health offices were more likely to use computers.

> *"There is already an established database up to Ministry of Health. Here in the District, it is totally electronic and we do not send data to the next level with a hard copy. Hard copy is only sent from lower level up-to to District level." (Focal person)*

Data were compiled mainly for reporting to the next level, with the exception of health centres, where performance monitoring teams used data to monitor health service delivery.

For peer review only - http://bmjopen.bmj.com/site/about/guidelines.xhtml

Little was done to triangulate different sources of data in the system. For instance, logistics data on drug consumption were merely used to validate the service delivery report.

> *"EPSA only knows consumption data and doesn't have patient data. It only compares what is supplied and what is consumed. Therefore, it is difficult to compare the discrepancy" (Administrative staff)*

Data quality check

Respondents described a formal approach to data quality checks, i.e., standard tools and procedures used to check RHIS data. This process addresses data quality attributes such as reporting timeliness and accuracy.

> *"Recorded data, report, register, and tally are crosschecked. If the three are equal, we said the data are quality…. Based on this the quality of data will be ranked. … The report and register will be checked for the specified period for each month." (Administrative staff)*

Data and reports were verified before being sent to the next level either through phone call or in person review. This approach was reported to be more common than use of standard tools for checking data quality. Respondents said challenges come from lack of transportation, or competing demands on time.

> *"As soon as the report is finalized, the health centre immediately reports to the district without any verification by the performance management team and the district health office then immediately send it to zone health office without a review. This is due to other competing priorities." (Focal person)*

Sometimes reports were amended without consulting the source:

For peer review only - http://bmjopen.bmj.com/site/about/guidelines.xhtml

"We will call and ask them to clarify. Most of the time, their phone will not work. Now for instance if they reported PCV 1 as zero or left it blank, I will take the figure of penta 1 because it is the same. I will take all antigens reported as first dose and third dose and fill the missing part.'' (Focal person)

Perceived quality of RHIS data

Most respondents agreed that the RHIS data lacked consistency and were reported late. Lack of consistency was attributed to incorrect recording, modification or manipulation of data to compensate for the lack of data or resulting from poor understanding of the RHIS process. (Table 2).

For peer review only - http://bmjopen.bmj.com/site/about/guidelines.xhtml

Table 2: Perceived data quality as reported by the respondents, Ethiopia, 2019/20

Perceived data quality issue	Illustrative quote
Data not recorded on time	*"Staff fill the registration over night when they have information that the supervisors from district health office will come." (Focal person)*
Wrong recording	*"The patients are taking drugs but are not reported. This creates under reporting. On the other hand, sometimes there is a practice of reporting patients of other diseases" (Health care provider)*
	"The health extension workers may include and report to us information which is not found in their tally sheet or register. That is what we evaluated." (Focal person)
Double counting	*"Yes! There is double reporting in ANC. They are confusing. I mean...ehh... if they did not understand well each other, who didn't go, who comes there (health centre & Hospital), who is referred, they might report twice. A mother who just got a first ANC service there (health post) and comes for second service (health centre/Hospital) is also reported as first ANC again..." (Administrative staff).*
Data manipulation	*"For instance, nutrition indicators are mostly reported as zero from the health post but sometimes we (the health centre) just put numbers that we think is appropriate by evaluating the health posts previous performance. And sometimes we get reports that are left blank and we just assume that as being zero and we fill the space with "0"." (Data manager)*
	"So far, we did not come across [any] neonatal death report. However, I could not say there is no neonatal death at all...The weakness here is the death is not correctly reported" (Data Manager).
Delayed reporting	*"The report doesn't come on time, for example the report is closed on 20th and from health posts it will be sent to us from 20th to 22th, we, in turn, we aggregate the health posts report including our health facility and we send the report to district until 26th of every month". (data manager)*
	"I would say the data has quality although there is a gap in timeliness. For example, one health post in our catchment area is relatively difficult for transportation. Due to that their report gets delayed for three or four days," (Focal person)

For peer review only - http://bmjopen.bmj.com/site/about/guidelines.xhtml

Respondents mentioned several reasons for inadequate data quality, presented below as technical, organizational or behavioural factors.

Technical factors

Respondents expressed concern about the number and complexity of forms. Parallel reporting posed additional burdens on the system and contributed to poor data quality, occurring because some indicators that are relevant to several programs were not captured centrally in the RHIS.

> *"Many partners need reports from us. Their data needs are different… The parallel report is still a problem and ignorance is there, in the higher level" (Administrative staff).*

Understanding indicators varied between respondents. Maternal health indicators such as first and fourth antenatal care visits were considered challenging, with additional complexity due to wrongly including information on gestational age:

> *"ANC1 is a visit by a woman for the first time. A pregnant woman within 16-24 week of gestational age is ANC1." (Health care provider)*

> *"….starting from the first visit, if a pregnant mother comes 28 week for the second, and 32 week for third, eeh….. 36 for third time consecutively and comes again from 36 to 40th week, I take her last visit as ANC4." (Health care provider)*

Understanding RHIS indicators was also limited by language issues as not all forms and job aids were translated into local languages. This posed a challenge especially at the health post.

> *"The problem is [the] integrated card and even [the family] folder is difficult to understand since it is in English" (Health care provider)*

For peer review only - http://bmjopen.bmj.com/site/about/guidelines.xhtml

"The Amharic version [of EPI card] was printed and distributed. How could the people do the work? Those down there [at health posts] do not understand Amharic. (Administrative staff).

Another cross-cutting technical issue was inappropriate denominators used for calculating health service coverage. Targets for different services were based on population estimates using the last available census from 2007. Thus, set targets can differ from actual numbers of individuals requiring the service (e.g., pregnant women or children eligible for vaccines) in a district or catchment.

"We are mostly being challenged by this [denominator issue]. For example, there is one kebele which was given a target of 46 for ANC service based on the population conversion factor, but there are only 18 pregnant mothers found in the kebele." (Administrative staff)

Not all health facilities had access to computers, but where health centres had computers and internet access and in most districts, reports were sent online. This was considered progress despite significant variations in use of technology.

"Out of the three health centres, one of them submits its report online. It is 22 km away from here, they have electricity but there was no connection, now the zonal health department provided them 3G CDMA [Code Division Multiple Access] and they are using that. The other two submits offline using a flash disk." (Focal person)

Organizational factors

The RHIS utilizes nationally developed standard forms and registers. Selected service registration books come from the ministry, while remaining forms are sent from regional,

For peer review only - http://bmjopen.bmj.com/site/about/guidelines.xhtml

zonal and district health offices. Shortage of supplies such as registration books, tally sheet and other forms were repeatedly mentioned.

> *"For example, now there is no tally sheet for postnatal, and even a registration book…it is not available in the district either. We are using attaching papers as register; we can show you …." (Health care provider)*

The district office diverted resources allocated to other activities or duplicated forms to address supply gaps. It was not uncommon for health care workers to use their own money.

> *"….budget is not allocated separately for activities related to health information, this is a problem in our district and it is also a problem in our zone, there is no direct budget allocated for this, we use from other funds that we get from aid." (Administrative staff)*

Limited electricity, computers and transportation often affected health posts. At health centres, frequent interruption of power coupled with lack of backup affected timely reporting, and availability of forms for registration and reporting. Table 3 lists resource and infrastructure challenges reported by respondents.

For peer review only

For peer review only - http://bmjopen.bmj.com/site/about/guidelines.xhtml

Table 3: Resource and infrastructure related challenges as reported by respondents, in Ethiopia, 2019/20

Resource constraint	Illustrative quote
Lack of transportation	*''Transportation is our biggest challenge. In the summer season, sometimes we can't send the report. It is difficult to cross the rivers. We try to cross by walking. Once when I was crossing the river, I lost my report papers by the flood'' (Health care provider).*
Lack or interruption of electricity	*"Especially [when] a report gets delayed; there is no backup, this power is not how you see it, sometimes when it interrupts it's not fixed soon; because of this, when power is off, everything disrupts, even we can't print; we can't send the report." (Administrative staff)*
No computer	*"It was not possible to send report using CDs (compact discs) as there were no computers in some places." (Focal person).*
	"There are a lot of Health centres that have no computer, and even those who have computers, some of them have no electricity." (Focal person).
Printer	*"Having printer is a problem, we [HEWs] can't get printed reporting forms when we need them, and it is not always available "(Health care provider).*
Poor access to internet	*"…Even in the areas where the online system is launched there is an internet problem. So generally, theoretically we are shifted to digitalization, [but] practically there is no enabling condition to digitalization." (Focal person)*
	"Since there is no regular telecommunication cable line we use offline; unfortunately, we have taken the computer to the district for installing the offline application and…, we believe its electronic based on the District health information system 2(DHIS2)." (Administrative staff)

Except in a few health facilities, health workers were responsible for RHIS activities in addition to their clinical work. Human resource shortages were more prominent at health post level where one or two health extension workers provide more than 16 health service packages and produce reports for each. This workload was said to contribute to poor data quality.

> *"….. because what comes from the districts puts pressure on us [health extension workers]. What comes from the (kebele) cabinet brings pressure on us [health extension workers]. There are times we even do agricultural activities, which doesn't*

For peer review only - http://bmjopen.bmj.com/site/about/guidelines.xhtml

concern us so it is very difficult. And when it is time to work on report, there are a lot of forms to fill and it is difficult for us." (Health care provider)

There was a clear demand for training although a few respondents mentioned that training hadn't posed problems. Where training was lacking, staff turnover was mentioned as the main cause. Moreover, recent changes to RHIS tools called for more training.

"Even we have no a clear understanding on the data element in the DHIS-2, the data elements are so many, it is not user-friendly. There is confusion among us which data element to use and the District level supervisor seems clueless on this issue as we have witnessed during the recent supervision" (Focal person).

District health offices supervise and support health centres in the district, and each health centre does the same for health posts in its catchment area. There is also a performance monitoring team at the health centre that should provide regular feedback to health centres and health posts. However, supervision was said to be infrequent and not always supportive.

"They came once or twice per year. In the last three months, no one came to our health post from health centre or district [district] or zone." (Health care provider)

Supervision was said to rarely focus on data quality. Furthermore, supervisory staff were considered inexperienced in providing technical support on data quality to lower level staff.

"The support focuses on technical coaching on the [health] service, but not on the data quality" (Health care provider)

The local performance monitoring team serves as a check-and balance system; it monitors the service delivery output and provides the necessary support to improve performance as

For peer review only - http://bmjopen.bmj.com/site/about/guidelines.xhtml

well as data quality. However, several respondents reported that the team met infrequently and was sub-optimal.

Respondents, including administrative staff, believed there ought to ways to holding people accountable when data quality is compromised. It was felt over-reporting of health service coverage rewards health facilities, which are seen to achieve targets without anyone confirmation of reporting accuracy.

> *"If there is any reward planned from the higher level, it will go directly to those who reported higher coverage. When additional budget is assigned, the district with higher coverage is given priority. Other districts see this and inflate their coverage to get the same advantage and never report the actual figures." (Focal person)*

There was also fear of reporting low service coverage or unwanted results such as neonatal death, leading to data manipulation to please higher-level administrative staff.

> *"I want to report the actual figures, by the way I am happy when you told me to interview me without the presence of my boss, because it is hard to explain in his presence. For instance, there is an intention to over report delivery service and decrease or report zero for still births and the like." (Focal person)*

Behavioural factors

Gaps in knowledge and skill related to the RHIS process was expressed by administrative staff and some health care providers, including difficulties understanding the registration and other forms, performance management, and basics of data entry and analysis. In addition, lack of knowledge and skill on checking data quality was reported.

For peer review only - http://bmjopen.bmj.com/site/about/guidelines.xhtml

For peer review only

"We do not have information and skill on how to work on the quality of data and we have limited knowledge on how to work on performance management, comparison and so on." (Focal person).

Health workers repeatedly mentioned lack of interest in RHIS resulting from low personal motivation and work overload.

"Sometimes we get fed up, because the format asks for too many things and we don't understand, we say: -What? We don't fill it and we submit without filling the information" (Health care provider)

Perceived use of data

A culture of data use was not well developed and the utility of generating data routinely not well understood.

"The purpose of the analysed health data is for decision making, this is the fact, but still there is a gap in using the data. It should be good if the stakeholders of the health facility use the analysed data". (Administrative staff)

Data use for programming was appreciated more at higher levels of the health system. It was reported that data were used for monitoring performance and identifying gaps during annual planning or to manage drug supply. There were also initiatives as reported by administrative staff to improve data use.

"I believe that conducting data verification regularly at lower level and provide close support to the Health centre and Health post staff will help to improve data quality and use problem" (focal person)

For peer review only - http://bmjopen.bmj.com/site/about/guidelines.xhtml

Discussion (W835)

We assessed quality of RHIS data in Ethiopia across multiple health indicators and explored reasons affecting quality, from data generation through to reporting and use. We observed variations in quality between indicators. Whereas there was timely reporting of some indicators but with less accuracy, others were reported accurately, but not on time or completely, adding to concerns about RHIS data quality and utility. Determinants of data quality ranged from simple logistical issues, such as supply of registry books, to complex technical issues, such as the size of a target population used as the denominator to calculate coverage. Organizational factors related to training and supervision stretched into more complex behavioural issues of motivation and fear of reporting unfavourable events.

One strength of this study is that we interviewed over 100 informants representing a mix of staff in the health system and achieved thematic saturation, suggesting our findings have relevance throughout the Ethiopian health system. We also tested the credibility of our result using a member check approach and confirmed the results. A potential limitation of this study was the small quantitative assessment sample; however, this part of the study was designed to prepare the background for the in-depth qualitative assessment rather than to yield statistically representative results. Our qualitative findings reflected similar data quality problems.

Both quantitative and qualitative results confirmed limited availability of source documents. Availability varied by indicator, and only one indicator had source documents for the whole observation period. Respondents described registration book and tally sheet shortages.

Completeness, timeliness, and accuracy of reporting were found to be inadequate for selected key indicators. Endriyas et al. showed a similar pattern of variability of accuracy

For peer review only - http://bmjopen.bmj.com/site/about/guidelines.xhtml

among indictors in Ethiopia, with maternal indicators exhibiting better quality [7]. This may result from a national focus on maternal and child health services. Endriyas et al. and other studies have also described over-reporting of service coverage and under-reporting of disease similar to our findings [21–23].

Complexity of registration forms and language barriers detrimentally affect accurate data recording [23,24]. While inadequate knowledge of RHIS is a cross-cutting issue, it proved more problematic at lower levels of the health system, where data are generated. Other studies report that not understanding indicators [25] and poor competency in recording [26] affect data quality.

Human resource shortages appeared to affect all levels of the RHIS process, most prominently at health facilities, where health workers are responsible for data collection on top of their clinical service. This creates workload and reduces motivation for RHIS. Similar human resource challenges have been found elsewhere [7,12,23,26]. Furthermore, access to technology that might ease this workload remains low. Disruption and shortages of data collection forms and registration books also contributed to delayed or inaccurate recording. Others have found that simplified data collection forms or digital tools can reduce the RHIS burden [27] and improve data quality [28,29].

The delay in data transmission emerged as a common problem at health facility level. As mentioned above, access to technology such as computers and internet would improve timely data transmission, although this would not address the problem of parallel reporting requirements that also add to workload and reporting delays, as cited by Gebreslassie et al. [30].

For peer review only - http://bmjopen.bmj.com/site/about/guidelines.xhtml

Data processing and analysis occurred primarily at higher levels. Gaps in knowledge and skill were reported to challenge these processes in other settings [26,31]. Use of outdated population data for denominators has already been raised as a concern in previous analyses of Ethiopian RHIS [11]. Similarly, inconsistency of denominators used to estimate coverage was reported by Bosch-Capblanch et al [21].

Although data quality checking and feedback systems using standard tools exist, these are rarely implemented. Other studies have noted this determinant of poor data quality [7,23,30,32], and have shown that regular data quality assurance with appropriate feedback can motivate positive changes in data quality and use [16,33]. What was unique here was the establishment of performance monitoring teams to oversee activities in the health system including data quality, but lack of budget and gap in skills negatively affected the functionality of this mechanism.

Although staff fear reporting unfavourable data, we nonetheless found demand for a system that holds health workers and health facilities accountable for generating inaccurate data.

In terms of data use, this was uncommon at sites of data generation although administrative staff did employ local data for planning and monitoring local performance. Similar findings were reported elsewhere [14,22]. Many studies have recognized the effect data use and data quality have on one another [4,7,16,34].

In summary, many factors negatively affecting data quality persist within Ethiopia's RHIS. Some of these factors could be tackled with existing resources, such as ensuring availability of registration forms and tally sheets in local languages. On-the-job training for health care workers at the lower level can boost their knowledge and skills, but also their motivation.

For peer review only - http://bmjopen.bmj.com/site/about/guidelines.xhtml

For peer review only

Strengthening the existing data quality and feedback system is essential. Human resources for RHIS, infrastructure and budget are cross-cutting factors that affect the whole RHIS process and require longer-term planning and multi-sectoral engagement.

Figure 1 Availability of source documents and reports for the facility-months observed

Figure 2 Completeness, timeliness and accuracy of reporting for selected indicators in the routine health information system

Figure 3 Accuracy of reporting for selected indicators in the routine health information system

Ethics approval

The ORCA thematic groups' proposals were reviewed and approved by the EPHI Institutional Review Board (EPHI-IRB-188-2019, EPHI-IRB-196-2019, EPHI-IRB-190-2019, EPHI-IRB-202-2019, EPHI-IRB-189-2019, and EPHI-IRB-2014-2019). Permission was also obtained to conduct the fieldwork from each regional health office, district health office and health facility visited prior to data collection. Written informed consent was obtained from all participants and measures taken to ensure anonymity. Translators were not chosen among supervisory staff or others on whom the respondent could be dependent. Staff categories were expressed in general terms, such as "administrative" to ensure anonymity.

Abbreviations

EPHI Ethiopia Public Health Institute

EPSA Ethiopian Pharmaceutical Supply Agency

LSHTM London School of Hygiene and Tropical Medicine

MOH Ministry of Health

ORCA Operational Research and Coaching for Analysts

PRISM Performance of Routine Information System Management

For peer review only - http://bmjopen.bmj.com/site/about/guidelines.xhtml

RHIS Routine Health Information System

VF Verification Factor

Patient and public involvement

Patients or the public were not involved in the design, or conduct, or reporting, or dissemination plans of our research.

Acknowledgment

The authors thank the study participants for taking part in this research process and shared their experience. The authors are grateful for the logistic support received from LSHTM Ethiopia team, and Ethiopian public health institute.

Funding statement

The ORCA project was funded by the Bill and Melinda Gates Foundation with a grant to the London School of Hygiene & Tropical Medicine (INV-010320).

Competing interest statement

No potential conflict of interest reported by the authors.

Data availability statement

The quantitative data are available upon request to the corresponding author.

Author contributions

All members of the ORCA team contributed to designing and conducting the study. the following authors drafted the manuscript collaboratively: Abyot Adane, Tewabe M. Adege, Habtamu A. Anteneh, Emiamrew S. Ayalew, Della Berhanu, Netsanet Berhanu, Misrak G. Beyene, Tesfahun Bishaw, Joanna Busza, Eshetu Cherinet, Mamo Dereje, Tsega H. Desta,

For peer review only - http://bmjopen.bmj.com/site/about/guidelines.xhtml

Abera Dibabe, Heven S. Firew, Freweini Gebrehiwot, Etenesh Gebreyohannes, Zenebech Gella, Addis Girma, Zuriash Halefom, Sorsa F. Jama, Annika Janson, Binyam Kemal, Yidnekachew D. Mazengiya, Meresha W. Nega, Israel A. Otoro, Tefera Taddele, Gulilat Tefera, Admasu Teketel, Miraf Tesfaye, Tsion Tsegaye, Kidist Woldesenbet, Yakob Wondarad, Zemzem M. Yusuf, Kidist Zealiyas, Mebratom H. Zeweli, Lars Åke Persson, and Seblewengel Lemma. All authors contributed in the later steps of the writing process and approved of the final manuscript and agreed to be accountable for all aspects of the work.

For peer review only

For peer review only - http://bmjopen.bmj.com/site/about/guidelines.xhtml

References

1 World Health Organization, editor. *Monitoring the building blocks of health systems: a handbook of indicators and their measurement strategies*. Geneva: : World Health Organization 2010.

2 Kumar M, Gotz D, Nutley T, *et al.* Research gaps in routine health information system design barriers to data quality and use in low- and middle-income countries: a literature review. *Int J Health Plann Manage* 2018;**33**.

3 Wagenaar BH, Sherr K, Fernandes Q, *et al.* Using routine health information systems for well-designed health evaluations in low- and middle-income countries. *Health Policy Plan* 2016;**31**:129–35. doi:10.1093/heapol/czv029

4 Wagenaar BH, Hirschhorn LR, Henley C, *et al.* Data-driven quality improvement in low- and middle-income country health systems: lessons from seven years of implementation experience across Mozambique, Rwanda, and Zambia. *BMC Health Serv Res* 2017;**17**:830. doi:10.1186/s12913-017-2661-x

5 Hagaman AK, Singh K, Abate M, *et al.* The impacts of quality improvement on maternal and newborn health: preliminary findings from a health system integrated intervention in four Ethiopian regions. *BMC Health Serv Res* 2020;**20**:522. doi:10.1186/s12913-020-05391-3

6 Ouedraogo M, Kurji J, Abebe L, *et al.* A quality assessment of Health Management Information System (HMIS) data for maternal and child health in Jimma Zone, Ethiopia. *PLoS One* 2019;**14**:e0213600. doi:10.1371/journal.pone.0213600

7 Endriyas M, Alano A, Mekonnen E, *et al.* Understanding performance data: health management information system data accuracy in Southern Nations Nationalities and People's Region, Ethiopia. *BMC Health Serv Res* 2019;**19**:175. doi:10.1186/s12913-019-3991-7

8 Ministry of Health, Ethiopia. Health Sector Transformation Plan (HSTP) 2015/16 - 2019/20. 2015.https://www.globalfinancingfacility.org/sites/gff_new/files/Ethiopia-health-system-transformation-plan.pdf (accessed 9 Oct 2019).

9 Dehnavieh R, Haghdoost A, Khosravi A, *et al.* The District Health Information System (DHIS2): A literature review and meta-synthesis of its strengths and operational challenges based on the experiences of 11 countries. *Health Inf Manag* 2019;**48**:62–7. doi:10.1177/1833358318777713

10 Thangasamy P, Gebremichael M, Kebede M, *et al.* A pilot study on District Health Information Software 2: challenges and lessons learned in a developing country: an experience from Ethiopia. *International Research Journal of Engineering and Technology* 2016;**03**:7.

11 Adane A, Adege TM, Ahmed MM, *et al.* Routine health management information system data in Ethiopia: consistency, trends, and challenges. *Global Health Action* 2021;**14**:1868961. doi:10.1080/16549716.2020.1868961

For peer review only - http://bmjopen.bmj.com/site/about/guidelines.xhtml

12 Teklegiorgis K, Tadesse K, Mirutse G, *et al.* Level of data quality from Health Management Information Systems in a resources limited setting and its associated factors, eastern Ethiopia. *S Afr J Inf Manag* 2016;**18**:8. doi:10.4102/sajim.v18i1.612

13 Dagnew E, Woreta SA, Shiferaw AM. Routine health information utilization and associated factors among health care professionals working at public health institution in North Gondar, Northwest Ethiopia. *BMC Health Serv Res* 2018;**18**:685. doi:10.1186/s12913-018-3498-7

14 Shiferaw AM, Zegeye DT, Assefa S, *et al.* Routine health information system utilization and factors associated thereof among health workers at government health institutions in East Gojjam Zone, Northwest Ethiopia. *BMC Med Inform Decis Mak* 2017;**17**:116. doi:10.1186/s12911-017-0509-2

15 Bhattacharya AA, Umar N, Audu A, *et al.* Quality of routine facility data for monitoring priority maternal and newborn indicators in DHIS2: A case study from Gombe State, Nigeria. *PLoS One* 2019;**14**:e0211265. doi:10.1371/journal.pone.0211265

16 Gimbel S, Mwanza M, Nisingizwe MP, *et al.* Improving data quality across 3 sub-Saharan African countries using the Consolidated Framework for Implementation Research (CFIR): results from the African Health Initiative. (Implementation science as an essential driver for sustainable health systems strengthening interventions: Lessons learned across the five-country African Health Initiative.). *BMC Health Serv Res* Published Online First: 2017. doi:10.1186/s12913-017-2660-y

17 Maokola W, Willey BA, Shirima K, *et al.* Enhancing the routine health information system in rural southern Tanzania: successes, challenges and lessons learned. *Trop Med Int Health* 2011;**16**:721–30. doi:10.1111/j.1365-3156.2011.02751.x

18 Aqil A, Lippeveld T, Hozumi D. PRISM framework: a paradigm shift for designing, strengthening and evaluating routine health information systems. *Health Policy Plan* 2009;**24**:217–28. doi:10.1093/heapol/czp010

19 Lemma S, Janson A, Persson L-Å, *et al.* Improving quality and use of routine health information system data in low- and middle-income countries: A scoping review. *PLoS One* 2020;**15**:e0239683. doi:10.1371/journal.pone.0239683

20 World Health Organization. Data Quality Review (DQR) Toolkit. 2017.www.who.int

21 Bosch-Capblanch X, Ronveaux O, Doyle V, *et al.* Accuracy and quality of immunization information systems in forty-one low income countries. *Trop Med Int Health* 2009;**14**:2–10. doi:10.1111/j.1365-3156.2008.02181.x

22 Ohiri K, Ukoha NK, Nwangwu CW, *et al.* An Assessment of Data Availability, Quality, and Use in Malaria Program Decision Making in Nigeria. *Health Syst Reform* 2016;**2**:319–30. doi:10.1080/23288604.2016.1234864

23 Roomaney A, Pillay-Van Wyk V, Awotiwon OF, *et al.* Availability and quality of routine morbidity data: Review of studies in South Africa. *J Am Med Inform Assoc* Published Online First: April 2017. doi:10.1093/jamia/ocw075

For peer review only

For peer review only - http://bmjopen.bmj.com/site/about/guidelines.xhtml

24 Regeru RN, Taegtmeyer M, Kumar M, *et al.* Exploring data quality in community health information systems in Kenya: A mixed method study. *BMC Proceedings* Published Online First: September 2017. doi:10.1186/s12919-017-0074-9

25 Assefa Ayalew G, Molla Teferi B, Mengistu Mitiku A, *et al.* Enhancing health facility-based data quality and use for decision making at primary health care units to improve health service delivery of maternal newborn child and adolescent health, Tigray Ethiopia 2018. *Archives of Community Medicine and Public Health* 2020;**6**:031–5. doi:10.17352/2455-5479.000068

26 Nicol E, Dudley L, Bradshaw D. Assessing the quality of routine data for the prevention of mother-to-child transmission of HIV: An analytical observational study in two health districts with high HIV prevalence in South Africa. *Int J Med Inform* 2016;**95**:60–70. doi:10.1016/j.ijmedinf.2016.09.006

27 Mutale W, Chintu N, Amoroso C, *et al.* Improving health information systems for decision making across five sub-Saharan African countries: Implementation strategies from the African Health Initiative. *BMC Health Serv Res* 2013;**1**:S9. doi:10.1186/1472-6963-13-S2-S9

28 Lazzerini M, Senanayake H, Mohamed R, *et al.* Implementation of an individual patient prospective database of hospital births in Sri Lanka and its use for improving quality of care. *BMJ OPEN* 2019;**9**. doi:10.1136/bmjopen-2018-023706

29 Ndira SP, Rosenberger KD, Wetter T. Assessment of Data Quality of and Staff Satisfaction with an Electronic Health Record System in a Developing Country (Uganda): A Qualitative and Quantitative Comparative Study. *Methods Inf Med* 2008;**47**:489–98. doi:10.3414/ME0511

30 Gebreslassie AA, Below MT, Ashebir MM, *et al.* Enhancing health facility-based data quality and use for decision making at primary health care units to improve health service delivery of maternal newborn child and adolescent health, Tigray Ethiopia 2018. *Arch Community Med Public Health* 2020;**6**:031–5.

31 Nicol E, Hanmer LA. Routine Health Information Systems in South Africa--Opportunities for Improvement. *Stud Health Technol Inform* 2015;**1**:993.

32 Gumede-Moyo S, Todd J, Bond V, *et al.* A qualitative inquiry into implementing an electronic health record system (SmartCare) for prevention of mother-to-child transmission data in Zambia: a retrospective study. *BMJ Open* 2019;**9**:e030428. doi:10.1136/bmjopen-2019-030428

33 Yourkavitch J, Zalisk K, Prosnitz D, *et al.* How do we know? An assessment of integrated community case management data quality in four districts of Malawi. *Health Policy Plan* 2016;**31**:1162–71. doi:10.1093/heapol/czw047

34 Braa J, Heywood A, Sahay S. Improving quality and use of data through data-use workshops: Zanzibar, United Republic of Tanzania. (Special issue: e-health.). *Bull World Health Organ* 2012;**90**:379–84. doi:10.2471/BLT.11.99580

For peer review only - http://bmjopen.bmj.com/site/about/guidelines.xhtml

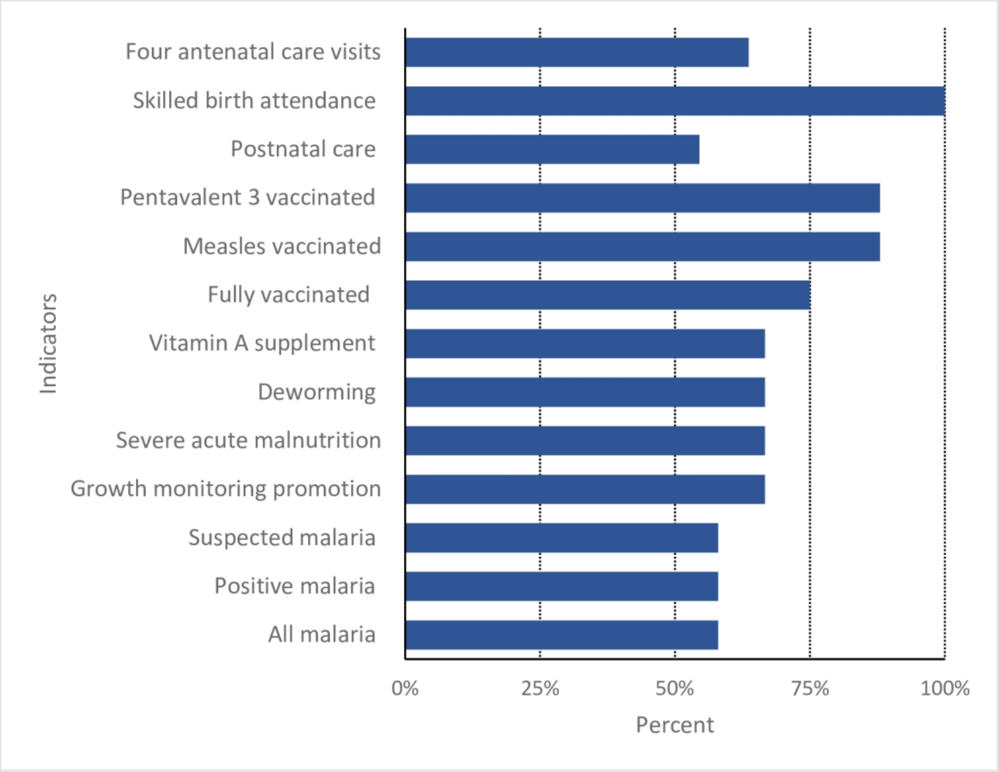

Figure 1 Availability of source documents and reports for the facility-months observed

140x108mm (300 x 300 DPI)

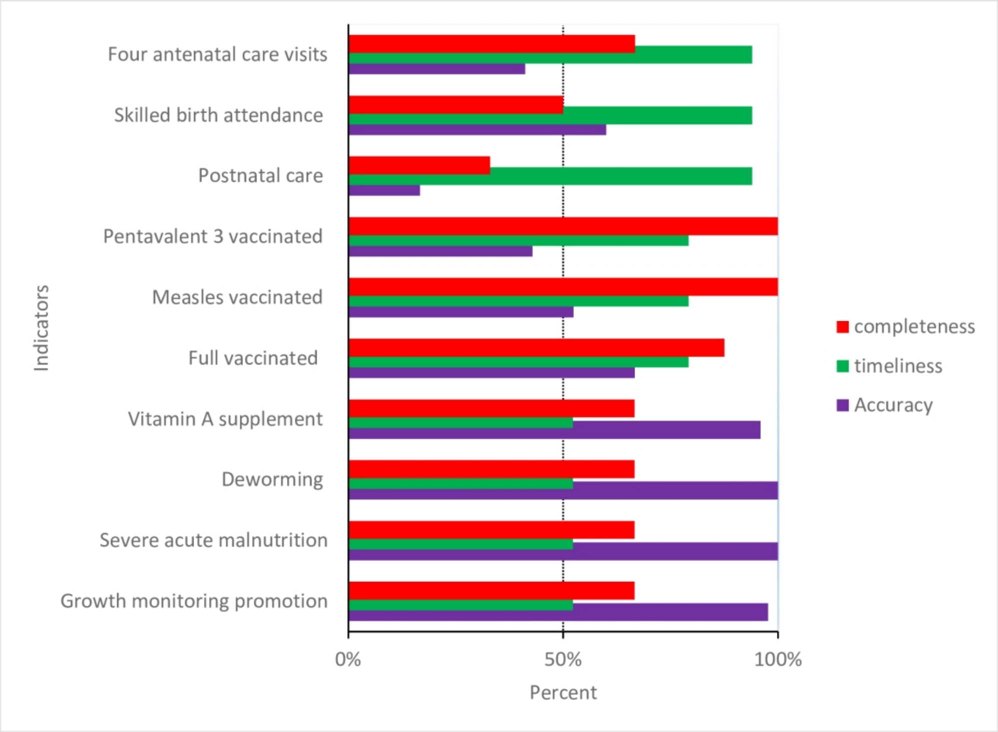

Figure 2 Completeness, timeliness and accuracy of reporting for selected indicators in the routine health information system

162x119mm (300 x 300 DPI)

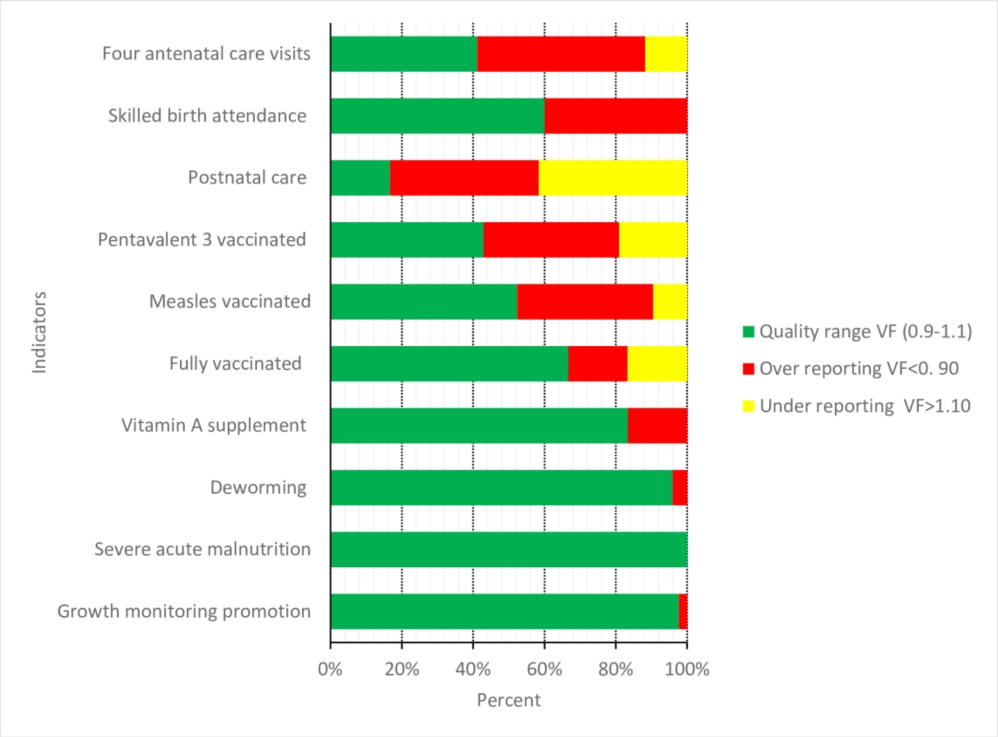

Figure 3 Accuracy of reporting for selected indicators in the routine health information system

171x126mm (300 x 300 DPI)

BMJ Open

Exploring data quality and use of the routine health information system in Ethiopia: a mixed-methods study

Journal:	*BMJ Open*
Manuscript ID	bmjopen-2021-050356.R1
Article Type:	Original research
Date Submitted by the Author:	22-Sep-2021

Complete List of Authors:	Adane, Abyot; Ethiopian Pharmaceutical Supply Agency<br>Adege, Tewabe M.; Ethiopia Ministry of Health<br>Ahmed, Mesoud M.; Ethiopia Ministry of Health<br>Anteneh, Habtamu A.; Ethiopia Ministry of Health<br>Ayalew, Emiamrew S.; Ethiopia Ministry of Health<br>Berhanu, Della; London School of Hygiene & Tropical Medicine<br>Berhanu, Netsanet; Ethiopia Ministry of Health<br>Getnet, Misrak; Ethiopian Public Health Institute<br>Bishaw, Tesfahun; Ethiopia Ministry of Health<br>Busza, Joanna; London School of Hygiene & Tropical Medicine<br>Cherinet, Eshetu; Ethiopia Ministry of Health<br>Dereje, Mamo; Ethiopia Ministry of Health<br>Desta, Tsega H; Ethiopia Ministry of Health<br>Dibabe, Abera; Ethiopia Ministry of Health<br>Firew, Heven S.; Ethiopian Public Health Institute<br>Gebrehiwot, Freweini; Ethiopian Public Health Institute<br>Gebreyohannes, Etenesh; Ethiopia Ministry of Health<br>Gella, Zenebech; Ethiopia Ministry of Health<br>Girma, Addis; Ethiopia Ministry of Health<br>Halefom, Zuriash; Ethiopia Ministry of Health<br>Jama, Sorsa F.; Ethiopia Ministry of Health<br>Janson, Annika; London School of Hygiene & Tropical Medicine<br>Kemal, Binyam; Ethiopia Ministry of Health<br>Kiflom, Abiy; Ethiopian Pharmaceutical Supply Agency<br>Mazengiya, Yidnekachew D.; Ethiopia Ministry of Health<br>Mekete, Kalkidan; Ethiopian Public Health Institute<br>Mengesha, Magdelawit; Ethiopia Ministry of Health<br>Nega, Meresha W.; Ethiopia Ministry of Health<br>Otoro, Israel A.; Ethiopia Ministry of Health<br>Schellenberg, Joanna; London School of Hygiene & Tropical Medicine<br>Taddele, Tefera; Ethiopian Public Health Institute<br>Tefera, Gulilat; Ethiopian Pharmaceutical Supply Agency<br>Teketel, Admasu; Ethiopian Pharmaceutical Supply Agency<br>Tesfaye, Miraf; Ethiopia Ministry of Health<br>Tsegaye, Tsion; Ethiopian Pharmaceutical Supply Agency<br>Woldesenbet, Kidist; Ethiopia Ministry of Health<br>Wondarad, Yakob; Ethiopia Ministry of Health<br>Yusuf, Zemzem M.; Ethiopia Ministry of Health<br>Zealiyas , Kidist; Ethiopian Public Health Institute<br>Zeweli, Mebratom H.; Ethiopia Ministry of Health<br>Persson, Lars; London School of Hygiene & Tropical Medicine

	Lemma, Seblewengel ; London School of Hygiene & Tropical Medicine
**Primary Subject Heading**:	Health informatics
Secondary Subject Heading:	Health services research
Keywords:	PUBLIC HEALTH, HEALTH SERVICES ADMINISTRATION & MANAGEMENT, Health informatics < BIOTECHNOLOGY & BIOINFORMATICS

SCHOLARONE™
Manuscripts

I, the Submitting Author has the right to grant and does grant on behalf of all authors of the Work (as defined in the below author licence), an exclusive licence and/or a non-exclusive licence for contributions from authors who are: i) UK Crown employees; ii) where BMJ has agreed a CC-BY licence shall apply, and/or iii) in accordance with the terms applicable for US Federal Government officers or employees acting as part of their official duties; on a worldwide, perpetual, irrevocable, royalty-free basis to BMJ Publishing Group Ltd ("BMJ") its licensees and where the relevant Journal is co-owned by BMJ to the co-owners of the Journal, to publish the Work in this journal and any other BMJ products and to exploit all rights, as set out in our licence.

The Submitting Author accepts and understands that any supply made under these terms is made by BMJ to the Submitting Author unless you are acting as an employee on behalf of your employer or a postgraduate student of an affiliated institution which is paying any applicable article publishing charge ("APC") for Open Access articles. Where the Submitting Author wishes to make the Work available on an Open Access basis (and intends to pay the relevant APC), the terms of reuse of such Open Access shall be governed by a Creative Commons licence – details of these licences and which Creative Commons licence will apply to this Work are set out in our licence referred to above.

Other than as permitted in any relevant BMJ Author's Self Archiving Policies, I confirm this Work has not been accepted for publication elsewhere, is not being considered for publication elsewhere and does not duplicate material already published. I confirm all authors consent to publication of this Work and authorise the granting of this licence.

For peer review only

Exploring data quality and use of the routine health information system in Ethiopia: a mixed-methods study

The Operational Research and Coaching for Analysts (ORCA)-team

Abyot Adane,[1] Tewabe M. Adege,[2] Mesoud M. Ahmed,[2] Habtamu A. Anteneh,[2] Emiamrew S. Ayalew,[2] Della Berhanu,[3] Netsanet Berhanu,[2] Misrak Getnet,[4] Tesfahun Bishaw,[2] Joanna Busza,[3] Eshetu Cherinet,[2] Mamo Dereje,[2] Tsega H. Desta,[2] Abera Dibabe,[2] Heven S. Firew,[4] Freweini Gebrehiwot,[4] Etenesh Gebreyohannes,[2] Zenebech Gella,[2] Addis Girma,[2] Zuriash Halefom,[2] Sorsa F. Jama,[2] Annika Janson,[3,6] Binyam Kemal,[2] Abiy Kiflom,[1] Yidnekachew D. Mazengiya,[2] Kalkidan Mekete,[4] Magdelawit Mengesha,[2] Meresha W. Nega,[2], Israel A. Otoro,[2] Joanna Schellenberg,[3] Tefera Taddele,[4] Gulilat Tefera,[1] Admasu Teketel,[1] Miraf Tesfaye,[2] Tsion Tsegaye,[1] Kidist Woldesenbet,[2] Yakob Wondarad,[2] Zemzem M. Yusuf,[2] Kidist Zealiyas,[4] Mebratom H. Zeweli,[2] Lars Åke Persson,[3] Seblewengel Lemma[3]

[1] Ethiopian Pharmaceutical Supply Agency, Addis Ababa, Ethiopia

[2] Ministry of Health, Addis Ababa, Ethiopia

[3] London School of Hygiene & Tropical Medicine, London, United Kingdom

[4] Ethiopian Public Health Institute, Addis Ababa, Ethiopia

[5] Department of Women´s and Children's Health, Karolinska Institutet, Stockholm, Sweden

Correspondence to: Dr Seblewengel Lemma

London School of Hygiene & Tropical Medicine, Based at the Ethiopian Public Health Institute

Addis Ababa, Ethiopia

Seblewengel.Abreham@lshtm.ac.uk;

Keywords:

Completeness, Data quality, Health Management Information System, Interviews, Perceptions, Performance of Routine Information System Management, Routine Health Information System, WHO Data Quality Review toolkit

For peer review only

For peer review only

For peer review only - http://bmjopen.bmj.com/site/about/guidelines.xhtml

e-mail addresses:

Abyot Adane: abyot.andu@gmail.com

Tewabe Manaye Adege: tewabemanaye@gmail.com

Mesoud Mohammed Ahmed: Mesoud.mohammed@gmail.com

Habtamu Alemay Anteneh: alemayhabtamu@gmail.com

Emiamrew Sisay Ayalew: emiamrew.sisay@moh.gov.et

Della Berhanu: della.berhanu@lshtm.ac.uk

Netsanet Berhanu: netsanet.berhanu@moh.gov.et

Misrak Getnet Beyene: misrakg81@gmail.com

Tesfahun Bishaw: bishawtesfahun@gmail.com

Joanna Busza: joanna.busza@lshtm.ac.uk

Eshetu Cherinet: eshecher2005@gmail.com

Mamo Dereje: mamodereje355@gmail.com

Tsega Hailu Desta: tsehame@gmail.com

Abera Dibabe: aberad968@gmail.com

Heven Sime Firew: hevensime@yahoo.com

Freweini Gebrehiwot: fre.geb2012@gmail.com

Etenesh Gebreyohannes: mhexpert3.mh@gmail.com

Zenebech Gella: gellazeniti@gmail.com

Addis Girma: addisgirma2016@gmail.com

Zuriash Halefom: zuriash.halefom@gmail.com

Sorsa Faltamo Jama: sorsa1982@gmail.com

Annika Janson: annika.janson@sll.se

Binyam Kemal: binyam.kemal@moh.gov.et

For peer review only

For peer review only - http://bmjopen.bmj.com/site/about/guidelines.xhtml

Abyi Kiflom: abiy.kiflom@gmail.com

Seblewengel Lemma: Seblewengel.Abreham@lshtm.ac.uk

Yidnekachew Degefaw Mazengiya: ydegefaw@gmail.com

Kalkidan Mekete: kalnyou@yahoo.com

Magdelawit Mengesha: magdelawit.mengesha@moh.gov.et

Meresha W. Nega: workumersha98@gmail.com

Israel Ataro Otoro: israelotoro1@gmail.com

Lars Åke Persson: lars.persson@lshtm.ac.uk

Joanna Schellenberg: joanna.schellenberg@lshtm.ac.uk

Kidist Zealiyas shita: kzealiyas@gmail.com

Tefera Taddele: tefetes@gmail.com

Gulilat Tefera: gulelatmam@gmail.com

Admasu Teketel: admasuteketel@yahoo.com

Miraf Tesfaye: tesfayemiraf@gmail.com

Tsion Tsegaye: tsi.destiny@gmail.com

Kidist Woldesenbet: kidistwoldesenbet@gmail.com

Yakob Wondarad: Yakobwondaradd@gmail.com

Zemzem Mohammed Yusuf: zemzem.mohammed@moh.gov.et

Mebratom Haile Zeweli: mebrahtom2007@gmail.com

Word count: Abstract 292/300; main text 4047/4000, six figures and tables

For peer review only

ORCID-ID:

Abyot Adane; 0000-0001-9831-3248

Tewabe Manaye Adege; 0000-0001-6894-9966

Mesoud Mohammed Ahmed; 0000-0002-4740-2272

Habtamu Alemay Anteneh; 0000-0003-3755-3357

Emiamrew Sisay Ayalew; 0000-0002-4857-5802

Della Berhanu; 0000-0002-4984-893X

Netsanet Brehanu; 0000-0002-3435-0741

Misrak Getnet Beyene; 0000-0002-6629-756X

Tesfahun Bishaw; 0000-0002-3149-8177

Joanna Busza; 0000-0001-6334-0318

Eshetu Cherinet; 0000-0003-2882-5998

Mamo Dereje; 0000-0002-6581-0923

Tsega Hailu Desta; 0000-0002-5246-9295

Abera Dibabe; 0000-0002-6269-1869

Zuriash Halefom; 0000-0002-6135-3489

Sorsa Faltamo Jama; 0000-0002-7637-3255

Heven Sime Firew; 0000-0002-2732-1635

Freweini Gebrehiwot; 0000-0002-6337-1576

Etenesh Gebreyohannes; 0000-0002-3956-0561

Zenebech Gella; 0000-0001-8881-0152

Addis Girma; 0000-0003-2800-2916

Annika Janson; 0000-0001-5106-5670

Binyam Kemal; 0000-0002-1307-7643

Abiy Kiflom; 0000-0001-9593-2799

Seblewengel Lemma; 0000-0001-5910-3723

Yidnekachew Degefaw Mazengiya; 0000-0002-0224-1890

Kalkidan Mekete; 0000-0002-1786-5161

Magdelawit Mengesha; 0000-0003-0523-1396

For peer review only

For peer review only - http://bmjopen.bmj.com/site/about/guidelines.xhtml

Meresha W. Nega; 0000-0001-8222-7572
Israel Ataro Otoro; 0000-0001-6533-080X
Lars Åke Persson; 0000-0003-0710-7954
Joanna Schellenberg; 0000-0002-0708-3676
Kidist Zealiyas Shita; 0000-0001-5248-5036
Tefera Taddele; 0000-0002-2327-136x
Gulilat Tefera; 0000-0001-9927-1780
Admasu Teketel; 0000-0002-3337-838X
Tsion Tsegaye; 000-0003-4737-6059
Miraf Tesfaye; 0000-0003-2726-050X
Kidist Woldesenbet; 0000-0003-1016-6656
Yakob Wondarad; 0000-0001-7415-5767
Zemzem Mohammed Yusuf; 0000-0002-3827-9225
Mebratom Haile Zeweli; 0000-0002-7845-2790

For peer review only

For peer review only - http://bmjopen.bmj.com/site/about/guidelines.xhtml

Abstract (292/300)

Objective A routine health information system (RHIS) enables decision-making in the health care system. We aimed to analyse data quality at the district and regional level and explore factors and perceptions affecting the quality and use of routine data.

Design This was a mixed-methods study. We used the World Health Organization toolkit for analysing data quality and interviewed staff at the point of data generation and along with the flow of data. Data were analysed using the Performance of Routine Information System Management framework.

Setting This study was performed in eight districts in four regions of Ethiopia. The study was nested within a two-year programme of the Operational Research and Coaching for government Analysts.

Participants We visited 45 health posts, 1 district hospital, 16 health centres, and eight district offices for analysis of routine RHIS data and interviewed 117 staff members for the qualitative assessment.

Outcome measures We assessed availability of source documents, completeness, timeliness, and accuracy of reporting of routine data, and explored data quality and use perceptions.

Results There was variable quality of both indicator and data element. Data on maternal health and immunization were of higher quality than data on child nutrition. Issues ranged from simple organizational factors, such as availability of register books, to intricate technical issues, like complexity of indicators and choice of denominators based on population estimates. Respondents showed knowledge of the reporting procedures, but

For peer review only

For peer review only - http://bmjopen.bmj.com/site/about/guidelines.xhtml

also demonstrated limited skills, lack of supportive supervision, and reporting to please the next level. We saw limited examples of the use of data by the staff who were responsible for data reporting.

Conclusion We identified important organizational, technical, behavioural, and process factors that need further attention to improve the quality and use of routine health information system data in Ethiopia.

Strengths and limitations of this study

- We assessed data quality and explored perceptions around data quality and use across a range of health indicators
- Over 100 staff from different levels of Ethiopia's health system were interviewed and we attained thematic saturation.
- The qualitative findings suggested similar data quality problems as the quantitative results
- We conducted a member check test, confirming that our results were credible
- Our results from the quantitative data have limited generalizability, because we took a small sample size which was purposive rather than representative.

Background (377)

High-quality, real-time data on the burden of disease and performance of the health sector are critical for decision-making and resource allocation [1]. A routine Health Information System (RHIS) aggregates information across the health system [2–4]. Despite improvements, efforts to increase coverage, quality, equity, and accountability of health services are often hampered by the lack of reliable data [5–7].

For peer review only - http://bmjopen.bmj.com/site/about/guidelines.xhtml

The Ethiopian Ministry of Health named the *Information Revolution* as one of four agendas in its first Health Sector Transformation Plan [8], aiming to advance information collection, analysis, presentation, and dissemination. RHIS data are generated at the point of service delivery at primary level (health posts, health centres, primary hospitals), secondary level (general hospitals) and tertiary-level health care (specialised hospitals). The web-based open-source computer software District Health Information System was introduced in 2015 [9,10]. Data are forwarded and aggregated at district, regional and national administrative levels. However, the quality and use of RHIS data continues to be a challenge in Ethiopia [11–14] and elsewhere [15–17].

Factors affecting data quality can be classified as technical, behavioural and organizational according to the Performance of Routine Information System Management (PRISM) framework. Technical factors relate to the ease of data collection, collation, analysis and reporting while behavioural factors include individuals' knowledge, attitude and skills related to RHIS processes. Organizational factors focus on availing human capital, infrastructure and a functional control system [18]. These factors directly affect RHIS performance but also interact with each other, requiring an integrated approach to produce favourable outcomes [19]. Understanding how these factors function at national level using the PRISM conceptual framework is an appropriate way to identify and implement appropriate interventions.

The overall aim of this study was to analyse RHIS data quality and use at district and regional levels, and explore perceptions of factors affecting data quality through a mixed-methods approach. This paper brings together findings from the Operational Research and Coaching for Analysts (ORCA) work at district and regional level to contribute to understanding and

For peer review only - http://bmjopen.bmj.com/site/about/guidelines.xhtml

strengthening the RHIS across the whole health system. The specific objectives were to analyse the timeliness, completeness, and accuracy of reporting of RHIS data generated at primary health care level, and to explore reasons for problems in data quality and use along the flow of data.

Methods (712)

Study setting and design

The Ethiopian Ministry of Health (MOH) initiated the ORCA project in collaboration with the Ethiopian Public Health Institute (EPHI), the Ethiopian Pharmaceutical Supply Agency (EPSA) and London School of Hygiene & Tropical Medicine (LSHTM). ORCA was designed to guide participants through a research cycle that diagnosed and investigated the current state of data quality and use within the Ethiopian health system, taking into consideration key strategic health metrics. A group of 36 analysts from the MOH, EPHI, and EPSA participated alongside their normal work duties from June 2018 to June 2020. The ORCA participants chose to work in six thematic groups: Maternal Health, Neonatal Survival, Immunization, Child Nutrition, Malaria, and Tuberculosis

This was a mixed-methods study performed by the ORCA participants including quantitative analysis of district-level data, complemented by qualitative interviews with key informants at different levels. Fieldwork was conducted by each ORCA thematic group. Data were collected in eight districts in four regions in Ethiopia (Afar, Oromia, Southern Nations, Nationalities and People's region, and Tigray), selected in consultation with the regional health offices, from August to December 2019.

Sampling and recruitment

For peer review only - http://bmjopen.bmj.com/site/about/guidelines.xhtml

Health centres and health posts providing services for more than one year were included in the quantitative data collection. In each district, aggregated data were also assessed at district health offices. For the qualitative assessment, key informants were recruited purposively along the flow of data from health posts, health centres, district health offices, zonal health offices, regional health bureaus and the MOH. Informants had served for at least one year in their respective post, and could provide in-depth information about RHIS data. The informants' professional designations were health extension worker, head of health facility, RHIS focal person, head of district health office, and program expert at district, zonal, regional or federal level.

Data collection and processing

Each ORCA thematic group prepared a desk review checklist for relevant indicators, drawing on standard data quality assessment tools [20] (supplementary file 1-4). The checklists were pre-tested in similar settings. Data were collected at health facilities from primary source documents and entered into Microsoft Excel for analysis.

A qualitative topic guide was prepared in English by each thematic group and translated into local languages (Amharic, Oromiffaa, Tigrigna or Afar). Interview guides were pre-tested and refined, and further adapted during fieldwork to improve comprehensibility. Data collectors were ORCA team members trained in qualitative and mixed-methods research. Interviews lasted 30 to 60 minutes, recorded, and field notes were taken by group members. After data collection, group members reflected on their work and identified points for exploration during subsequent interviews. Recordings were transcribed verbatim. Ten percent of the transcripts were cross-checked with the audio for completeness and accuracy.

Quantitative information

For peer review only - http://bmjopen.bmj.com/site/about/guidelines.xhtml

All definitions were based on the WHO toolkit [20]. Details of the toolkit were discussed in our previous similar work [11]; *Availability of source document and report* was presented as a percentage, i.e. facilities with records, divided by the total facility months investigated; *Completeness of reporting* indicated the percentage of monthly reports received by the next level; *Timeliness of reporting* covered the proportion sent on time; and *Accuracy of reporting* indicated the ratio of numbers recounted and classified as exact match, within the data quality range (0.9-1.1), over-reporting (<0.9), or under-reporting (>1.1). Results were categorized by type of indicators and presented as percentage of health facility months.

Qualitative data analysis

Each group conducted thematic content analysis. After reading the verbatim transcripts, all group members coded the same interview and agreed on a coding framework. The group members divided interviews among themselves for coding, and met regularly to add codes to capture emerging ideas. Groups categorized codes into broader thematic areas. Each group prepared a report on qualitative results that were shared across groups. The joint results from all six thematic groups were synthesised using the PRISM framework [18] (supplementary file 5). Regular discussions were held to reflect on similarities and differences across the data sets, check for outliers and contradictory findings, and agree on distribution of key themes within the simplified structure of the framework. Finally, the result was shared with seven respondents at MOH to check for credibility.

Patient and public involvement

Patients or the public were not involved in the design, or conduct, or reporting, or dissemination plans of our research.

For peer review only - http://bmjopen.bmj.com/site/about/guidelines.xhtml

Results (w2255)

In total, 62 facilities and eight district health offices were visited for analysis of RHIS data and 117 key informants were interviewed (Table 1). Of all interviews, 35/117 (30%) were with health extension workers at health posts.

Table 1: Desk reviews and qualitative interviews conducted by ORCA thematic groups and other background information, Ethiopia, 2019/20

**Characteristics**	**Desk review (n=70)**	**Qualitative interviews (n=117)**
**Gender**		
Male		75 (64%)
Female		42 (36%)
**Health facilities/ offices visited**		
Health Post	45 (64%)	35 (30%)
Health Centre	16 (23%)	33 (28%)
District Hospital	1 (1%)	2 (2%)
District Health Offices	8 (11%)	21 (18%)
Zonal health office	0 (0%)	1 (1%)
Regional health office	0 (0%)	17 (15%)
Federal ministry of Health	0 (0%)	8 (7%)
**Region**		
Tigray	5 (7%)	15 (13%)
Afar	19 (27%)	37 (32%)
Oromia	17 (24%)	25 (21%)
SNNPR	29 (41%)	32 (27%)
National	0 (0%)	8 (7%)
**Thematic group**		
Maternal health [1]	12 (17%)	18 (15%)
Neonatal Survival[2]	17 (24%)	14 (12%)
Immunization[3]	9 (13%)	12 (10%)
Child nutrition[4]	9 (13%)	25 (21%)

[1] 1st antenatal care, 4th antenatal care, postnatal care & skilled delivery
[2] early institutional death (0-6 days), early community death (0-6 days), live birth in Kebele
[3] Pentavalent vaccine third dose, Measles, fully vaccinated
[4] Vitamin A supplementation, Deworming, Severe acute malnutrition, Growth monitoring promotion

For peer review only - http://bmjopen.bmj.com/site/about/guidelines.xhtml

For peer review only

Malaria[5]	6 (9%)	17 (15%)
Tuberculosis[6]	17 (24%)	31 (27%)

Availability of source documents, completeness, timeliness and accuracy of reporting

The availability of source document ranged from 55% to 100%. Only documents for skilled birth attendance reached 100% in observed health facilities (fig 1).

The majority of indicators had gaps in reporting. Maternal health and postnatal indicators had the most gaps in reporting. Completeness of reporting for nutrition was also low, at slightly over 50% for the facility-months reviewed. Completeness was much higher for immunization. Timeliness was over 90% for maternal health indicators, whereas just over half of reports for nutrition indicators were submitted on time (fig 2).

Maternal and immunization indicators had lower proportions of reports within the range for acceptable quality, whereas nutrition indicators were mainly reported within the quality range. Varying levels of over-reporting were observed in all service coverage indicators, but not for severe acute malnutrition (fig 2 and 3).

Respondents' views on data processes and quality

Interview respondents reported that data generation and flow mostly occurred as intended. At health facilities, data were usually recorded by hand using standard on paper forms, while district health offices were more likely to use computers.

[5] Suspected malaria, Positive malaria, All malaria
[6] New and relapse tuberculosis, & Treated tuberculosis

For peer review only - http://bmjopen.bmj.com/site/about/guidelines.xhtml

“There is already an established database up to Ministry of Health. Here in the District, it is totally electronic and we do not send data to the next level with a hard copy. Hard copy is only sent from lower level up to District level.” (Focal person)

Data were compiled mainly for reporting to the next level, with the exception of health centres, where performance monitoring teams used data to monitor health service delivery. Little was done to triangulate different sources of data in the system. For instance, logistics data on drug consumption were merely used to validate the service delivery report.

“EPSA only knows consumption data and doesn’t have patient data. It only compares what is supplied and what is consumed. Therefore, it is difficult to compare the discrepancy” (Administrative staff)

Data quality check

Respondents described a formal approach to data quality checks, i.e., standard tools and procedures used to check RHIS data. This process addresses data quality attributes such as reporting timeliness and accuracy.

“Recorded data, report, register, and tally are crosschecked. If the three are equal, we said the data are quality…. Based on this the quality of data will be ranked. … The report and register will be checked for the specified period for each month.” (Administrative staff)

Data and reports were verified before being sent to the next level either through phone call or in person review. This approach was reported to be more common than use of standard tools for checking data quality. Respondents said challenges come from lack of transportation, or competing demands on time.

For peer review only - http://bmjopen.bmj.com/site/about/guidelines.xhtml

"As soon as the report is finalized, the health centre immediately reports to the district without any verification by the performance management team and the district health office then immediately send it to zone health office without a review. This is due to other competing priorities." (Focal person)

Sometimes reports were amended without consulting the source:

"We will call and ask them to clarify. Most of the time, their phone will not work. Now for instance if they reported PCV 1 as zero or left it blank, I will take the figure of penta 1 because it is the same. I will take all antigens reported as first dose and third dose and fill the missing part." (Focal person)

Perceived quality of RHIS data

Most respondents agreed that the RHIS data lacked consistency and were reported late. Lack of consistency was attributed to incorrect recording, modification or manipulation of data to compensate for the lack of data or resulting from poor understanding of the RHIS process. (Table 2).

Table 2: Perceived data quality as reported by the respondents, Ethiopia, 2019/20

Perceived data quality issue	Illustrative quote
Data not recorded on time	*"Staff fill the registration over night when they have information that the supervisors from district health office will come." (Focal person)*
Wrong recording	*"The patients are taking drugs but are not reported. This creates under reporting. On the other hand, sometimes there is a practice of reporting patients of other diseases" (Health care provider)*
	"The health extension workers may include and report to us information which is not found in their tally sheet or register. That is what we evaluated." (Focal person)
Double counting	*"Yes! There is double reporting in ANC. They are confusing. I mean...ehh... if they did not understand well each other, who didn't go, who comes there*

For peer review only - http://bmjopen.bmj.com/site/about/guidelines.xhtml

	(health centre & Hospital), who is referred, they might report twice. A mother who just got a first ANC service there (health post) and comes for second service (health centre/Hospital) is also reported as first ANC again..." (Administrative staff).
Data manipulation	*"For instance, nutrition indicators are mostly reported as zero from the health post but sometimes we (the health centre) just put numbers that we think is appropriate by evaluating the health posts previous performance. And sometimes we get reports that are left blank and we just assume that as being zero and we fill the space with "0"." (Data manager)*
	"So far, we did not come across [any] neonatal death report. However, I could not say there is no neonatal death at all...The weakness here is the death is not correctly reported" (Data Manager).
Delayed reporting	*"The report doesn't come on time, for example the report is closed on 20th and from health posts it will be sent to us from 20th to 22th, we, in turn, we aggregate the health posts report including our health facility and we send the report to district until 26th of every month". (data manager)*
	"I would say the data has quality although there is a gap in timeliness. For example, one health post in our catchment area is relatively difficult for transportation. Due to that their report gets delayed for three or four days," (Focal person)

Respondents mentioned several reasons for inadequate data quality, presented below as technical, organizational or behavioural factors.

Technical factors

Respondents expressed concern about the number and complexity of forms. Parallel reporting posed additional burdens on the system and contributed to poor data quality, occurring because some indicators that are relevant to several programs were not captured centrally in the RHIS.

> *"Many partners need reports from us. Their data needs are different… The parallel report is still a problem and ignorance is there, in the higher level" (Administrative staff).*

For peer review only - http://bmjopen.bmj.com/site/about/guidelines.xhtml

Understanding indicators varied between respondents. Maternal health indicators such as first and fourth antenatal care visits were considered challenging, with additional complexity due to wrongly including information on gestational age:

"ANC1 is a visit by a woman for the first time. A pregnant woman within 16-24 week of gestational age is ANC1." (Health care provider)

"….starting from the first visit, if a pregnant mother comes 28 week for the second, and 32 week for third, eeh….. 36 for third time consecutively and comes again from 36 to 40th week, I take her last visit as ANC4." (Health care provider)

Understanding RHIS indicators was also limited by language issues as not all forms and job aids were translated into local languages. This posed a challenge especially at the health post.

"The problem is [the] integrated card and even [the family] folder is difficult to understand since it is in English" (Health care provider)

"The Amharic version [of EPI card] was printed and distributed. How could the people do the work? Those down there [at health posts] do not understand Amharic. (Administrative staff).

Another cross-cutting technical issue was inappropriate denominators used for calculating health service coverage. Targets for different services were based on population estimates using the last available census from 2007. Thus, set targets can differ from actual numbers of individuals requiring the service (e.g., pregnant women or children eligible for vaccines) in a district or catchment.

"We are mostly being challenged by this [denominator issue]. For example, there is one kebele which was given a target of 46 for ANC service based on the population

For peer review only - http://bmjopen.bmj.com/site/about/guidelines.xhtml

conversion factor, but there are only 18 pregnant mothers found in the kebele." (Administrative staff)

Not all health facilities had access to computers, but where health centres had computers and internet access and in most districts, reports were sent online. This was considered progress despite significant variations in use of technology.

"Out of the three health centres, one of them submits its report online. It is 22 km away from here, they have electricity but there was no connection, now the zonal health department provided them 3G CDMA [Code Division Multiple Access] and they are using that. The other two submits offline using a flash disk." (Focal person)

Organizational factors

The RHIS utilizes nationally developed standard forms and registers. Selected service registration books come from the ministry, while remaining forms are sent from regional, zonal and district health offices. Shortage of supplies such as registration books, tally sheet and other forms were repeatedly mentioned.

"For example, now there is no tally sheet for postnatal, and even a registration book…it is not available in the district either. We are using attaching papers as register; we can show you …." (Health care provider)

The district office diverted resources allocated to other activities or duplicated forms to address supply gaps. It was not uncommon for health care workers to use their own money.

"….budget is not allocated separately for activities related to health information, this is a problem in our district and it is also a problem in our zone, there is no direct budget allocated for this, we use from other funds that we get from aid." (Administrative staff)

For peer review only - http://bmjopen.bmj.com/site/about/guidelines.xhtml

Limited electricity, computers and transportation often affected health posts. At health centres, frequent interruption of power coupled with lack of backup affected timely reporting, and availability of forms for registration and reporting. Table 3 lists resource and infrastructure challenges reported by respondents.

Table 3: Resource and infrastructure related challenges as reported by respondents, in Ethiopia, 2019/20

Resource constraint	Illustrative quote
Lack of transportation	*''Transportation is our biggest challenge. In the summer season, sometimes we can't send the report. It is difficult to cross the rivers. We try to cross by walking. Once when I was crossing the river, I lost my report papers by the flood'' (Health care provider).*
Lack or interruption of electricity	*"Especially [when] a report gets delayed; there is no backup, this power is not how you see it, sometimes when it interrupts it's not fixed soon; because of this, when power is off, everything disrupts, even we can't print; we can't send the report." (Administrative staff)*
No computer	*"It was not possible to send report using CDs (compact discs) as there were no computers in some places." (Focal person).*
	"There are a lot of Health centres that have no computer, and even those who have computers, some of them have no electricity." (Focal person).
Printer	*"Having printer is a problem, we [HEWs] can't get printed reporting forms when we need them, and it is not always available "(Health care provider).*
Poor access to internet	*"…Even in the areas where the online system is launched there is an internet problem. So generally, theoretically we are shifted to digitalization, [but] practically there is no enabling condition to digitalization." (Focal person)*
	"Since there is no regular telecommunication cable line we use offline; unfortunately, we have taken the computer to the district for installing the offline application and…, we believe its electronic based on the District health information system 2(DHIS2)." (Administrative staff)

Except in a few health facilities, health workers were responsible for RHIS activities in addition to their clinical work. Human resource shortages were more prominent at health post level where one or two health extension workers provide more than 16 health service packages and produce reports for each. This workload was said to contribute to poor data quality.

For peer review only - http://bmjopen.bmj.com/site/about/guidelines.xhtml

"….. because what comes from the districts puts pressure on us [health extension workers]. What comes from the (kebele) cabinet brings pressure on us [health extension workers]. There are times we even do agricultural activities, which doesn't concern us so it is very difficult. And when it is time to work on report, there are a lot of forms to fill and it is difficult for us." (Health care provider)

There was a clear demand for training although a few respondents mentioned that training hadn't posed problems. Where training was lacking, staff turnover was mentioned as the main cause. Moreover, recent changes to RHIS tools called for more training.

"Even we have no a clear understanding on the data element in the DHIS-2, the data elements are so many, it is not user-friendly. There is confusion among us which data element to use and the District level supervisor seems clueless on this issue as we have witnessed during the recent supervision" (Focal person).

District health offices supervise and support health centres in the district, and each health centre does the same for health posts in its catchment area. There is also a performance monitoring team at the health centre that should provide regular feedback to health centres and health posts. However, supervision was said to be infrequent and not always supportive.

"They came once or twice per year. In the last three months, no one came to our health post from health centre or district [district] or zone." (Health care provider)

Supervision was said to rarely focus on data quality. Furthermore, supervisory staff were considered inexperienced in providing technical support on data quality to lower level staff.

"The support focuses on technical coaching on the [health] service, but not on the data quality" (Health care provider)

For peer review only - http://bmjopen.bmj.com/site/about/guidelines.xhtml

The local performance monitoring team serves as a check-and balance system; it monitors the service delivery output and provides the necessary support to improve performance as well as data quality. However, several respondents reported that the team met infrequently and was sub-optimal.

Respondents, including administrative staff, believed there ought to ways to holding people accountable when data quality is compromised. It was felt over-reporting of health service coverage rewards health facilities, which are seen to achieve targets without anyone confirmation of reporting accuracy.

> *"If there is any reward planned from the higher level, it will go directly to those who reported higher coverage. When additional budget is assigned, the district with higher coverage is given priority. Other districts see this and inflate their coverage to get the same advantage and never report the actual figures." (Focal person)*

There was also fear of reporting low service coverage or unwanted results such as neonatal death, leading to data manipulation to please higher-level administrative staff.

> *"I want to report the actual figures, by the way I am happy when you told me to interview me without the presence of my boss, because it is hard to explain in his presence. For instance, there is an intention to over report delivery service and decrease or report zero for still births and the like." (Focal person)*

Behavioural factors

Gaps in knowledge and skill related to the RHIS process was expressed by administrative staff and some health care providers, including difficulties understanding the registration and

For peer review only - http://bmjopen.bmj.com/site/about/guidelines.xhtml

other forms, performance management, and basics of data entry and analysis. In addition, lack of knowledge and skill on checking data quality was reported.

> *"We do not have information and skill on how to work on the quality of data and we have limited knowledge on how to work on performance management, comparison and so on." (Focal person).*

Health workers repeatedly mentioned lack of interest in RHIS resulting from low personal motivation and work overload.

> *"Sometimes we get fed up, because the format asks for too many things and we don't understand, we say: -What? We don't fill it and we submit without filling the information" (Health care provider)*

Perceived use of data

A culture of data use was not well developed and the utility of generating data routinely not well understood.

> *"The purpose of the analysed health data is for decision making, this is the fact, but still there is a gap in using the data. It should be good if the stakeholders of the health facility use the analysed data". (Administrative staff)*

> *"Drugs are distributed to health posts monthly depending on the consumption status and we (Logistic focal) don't give them unless they report number of cases. Otherwise drugs will expire there..." (Focal person)*

Data use for programming was appreciated more at higher levels of the health system. It was reported that data were used for monitoring performance and identifying gaps during annual

For peer review only - http://bmjopen.bmj.com/site/about/guidelines.xhtml

planning or to manage drug supply. There were also initiatives as reported by administrative staff to improve data use.

"I believe that conducting data verification regularly at lower level and provide close support to the Health centre and Health post staff will help to improve data quality and use problem" (Focal person)

"We (nutrition expert) use HMIS data; we found over reporting and lack of reporting sometimes, conducted performance reviews; our data source [was] HMIS, besides, we use the nutrition data base as alternative source of information. …. Data utilization [is] better at woreda health office where nutrition experts are available." (Focal person)

Discussion (W969)

We assessed quality of RHIS data in Ethiopia across multiple health indicators and explored reasons affecting quality, from data generation through to reporting and use. We observed variations in quality between indicators. Whereas there was timely reporting of some indicators but with less accuracy, others were reported accurately, but not on time or completely, adding to concerns about RHIS data quality and utility. Determinants of data quality ranged from simple logistical issues, such as supply of registry books, to complex technical issues, such as the size of a target population used as the denominator to calculate coverage. Organizational factors related to training and supervision stretched into more complex behavioural issues of motivation and fear of reporting unfavourable events.

One strength of this study is that we interviewed over 100 informants representing a mix of staff in the health system and achieved thematic saturation, suggesting our findings have

For peer review only - http://bmjopen.bmj.com/site/about/guidelines.xhtml

relevance throughout the Ethiopian health system. We also tested the credibility of our result using a member check approach and confirmed the results. A potential limitation of this study was the small quantitative assessment sample; however, this part of the study was designed to prepare the background for the in-depth qualitative assessment rather than to yield statistically representative results. Our qualitative findings reflected similar data quality problems. The data quality assessment tool we used may not rule out mistakes or wrong reporting in the RHIS processes. It could be argued that PRISM framework used to guide our analysis may not clearly delineate some of the factors to either behavioural or organizational factor.

Both quantitative and qualitative results confirmed limited availability of source documents. Availability varied by indicator, and only one indicator had source documents for the whole observation period. Respondents described registration book and tally sheet shortages.

Completeness, timeliness, and accuracy of reporting were found to be inadequate for selected key indicators. Endriyas et al. showed a similar pattern of variability of accuracy among indictors in Ethiopia, with maternal indicators exhibiting better quality [7]. This may result from a national focus on maternal and child health services. Endriyas et al. and other studies have also described over-reporting of service coverage and under-reporting of disease similar to our findings [21–23].

Complexity of registration forms and language barriers detrimentally affect accurate data recording [23,24]. While inadequate knowledge of RHIS is a cross-cutting issue, it proved more problematic at lower levels of the health system, where data are generated. Other studies report that not understanding indicators [25] and poor competency in recording [26] affect data quality.

For peer review only - http://bmjopen.bmj.com/site/about/guidelines.xhtml

Human resource shortages appeared to affect all levels of the RHIS process, most prominently at health facilities, where health workers are responsible for data collection on top of their clinical service. This creates workload and reduces motivation for RHIS. Similar human resource challenges have been found elsewhere [7,12,23,26]. Furthermore, access to technology that might ease this workload remains low. Disruption and shortages of data collection forms and registration books also contributed to delayed or inaccurate recording. Others have found that simplified data collection forms or digital tools can reduce the RHIS burden [27] and improve data quality [28,29].

The delay in data transmission emerged as a common problem at health facility level. As mentioned above, access to technology such as computers and internet would improve timely data transmission, although this would not address the problem of parallel reporting requirements that also add to workload and reporting delays, as cited by Gebreslassie et al. [30].

Data processing and analysis occurred primarily at higher levels. Gaps in knowledge and skill were reported to challenge these processes in other settings [26,31]. Use of outdated population data for denominators has already been raised as a concern in previous analyses of Ethiopian RHIS [11]. Similarly, inconsistency of denominators used to estimate coverage was reported by Bosch-Capblanch et al [21].

Although data quality checking and feedback systems using standard tools exist, these are rarely implemented. Other studies have noted this determinant of poor data quality [7,23,30,32], and have shown that regular data quality assurance with appropriate feedback can motivate positive changes in data quality and use [16,33]. What was unique in this study was the establishment of performance monitoring teams to oversee activities in the health

For peer review only - http://bmjopen.bmj.com/site/about/guidelines.xhtml

system including data quality, but lack of budget and gap in skills negatively affected the functionality of this mechanism.

Although staff fear reporting unfavourable data, we nonetheless found hopes for a system that holds health workers and health facilities accountable for generating inaccurate data, even while long-term challenges persist. Respondents may have recognised elements of "blame culture" in the Ethiopian RHIS, described by others as emerging where hierarchical management structures reward compliance over efforts to expose poor quality and function [34]. The result is that staff eschew negative attention, which does not predispose them to raise awareness of systemic weaknesses or help develop genuine accountability.

In terms of data use, this was uncommon at sites of data generation although administrative staff did employ local data for planning and monitoring local performance. Similar findings were reported elsewhere [14,22]. Many studies have recognized the effect data use and data quality have on one another [4,7,16,35].

In summary, many factors negatively affecting data quality persist within Ethiopia's RHIS. Some of these factors could be tackled with existing resources, such as ensuring availability of registration forms and tally sheets in local languages. On-the-job training for health care workers at the lower level can boost their knowledge and skills, but also their motivation. Strengthening the existing data quality and feedback system is essential. Human resources for RHIS, infrastructure and budget are cross-cutting factors that affect the whole RHIS process and require longer-term planning and multi-sectoral engagement, as does introducing a work culture that values proactive challenges to existing weaknesses. More qualitative work on data use could help understand barriers that could be tackled.

Figure 1 Availability of source documents and reports for the facility-months observed

For peer review only - http://bmjopen.bmj.com/site/about/guidelines.xhtml

Figure 2 Completeness, timeliness and accuracy of reporting for selected indicators in the routine health information system

Figure 3 Accuracy of reporting for selected indicators in the routine health information system

Ethics approval

The ORCA thematic groups' proposals were reviewed and approved by the Ethiopian Public Health Institute Institutional Review Board (EPHI-IRB-188-2019, EPHI-IRB-196-2019, EPHI-IRB-190-2019, EPHI-IRB-202-2019, EPHI-IRB-189-2019, and EPHI-IRB-2014-2019). Permission was also obtained to conduct the fieldwork from each regional health office, district health office and health facility visited prior to data collection. Written informed consent was obtained from all participants and measures taken to ensure anonymity. Translators were not chosen among supervisory staff or others on whom the respondent could be dependent. Staff categories were expressed in general terms, such as "administrative" to ensure anonymity.

Abbreviations

EPHI Ethiopia Public Health Institute

EPSA Ethiopian Pharmaceutical Supply Agency

LSHTM London School of Hygiene and Tropical Medicine

MOH Ministry of Health

ORCA Operational Research and Coaching for Analysts

PRISM Performance of Routine Information System Management

RHIS Routine Health Information System

VF Verification Factor

Acknowledgment

For peer review only

For peer review only - http://bmjopen.bmj.com/site/about/guidelines.xhtml

The authors thank the study participants for taking part in this research process and shared their experience. The authors are grateful for the logistic support received from LSHTM Ethiopia team, and Ethiopian public health institute.

Funding statement

The ORCA project was funded by the Bill and Melinda Gates Foundation with a grant to the London School of Hygiene & Tropical Medicine, grant number: INV-010320.

Competing interest statement

No potential conflict of interest reported by the authors.

Data availability statement

The quantitative data are available upon request to the corresponding author.

Author contributions

All members of the ORCA team participated in designing and conducting the study. The following authors drafted the manuscript collaboratively: Abyot Adane, Tewabe M. Adege, Mesoud M. Ahmed, Habtamu A. Anteneh, Emiamrew S. Ayalew, Della Berhanu, Netsanet Berhanu, Misrak Getnet, Tesfahun Bishaw, Joanna Busza, Eshetu Cherinet, Mamo Dereje, Tsega H. Desta, Abera Dibabe, Heven S. Firew, Freweini Gebrehiwot, Etenesh Gebreyohannes, Zenebech Gella, Addis Girma, Zuriash Halefom, Sorsa F. Jama, Annika Janson, Binyam Kemal, Abiy Kiflom, Yidnekachew D. Mazengiya, Kalkidan Mekete, Magdelawit Mengesha, Meresha W. Nega, Israel A. Otoro, Joanna Schellenberg, Tefera Taddele, Gulilat Tefera, Admasu Teketel, Miraf Tesfaye, Tsion Tsegaye, Kidist Woldesenbet, Yakob Wondarad, Zemzem M. Yusuf, Kidist Zealiyas, Mebratom H. Zeweli, Lars Åke Persson, and Seblewengel Lemma. All authors contributed in the later steps of the writing process

For peer review only - http://bmjopen.bmj.com/site/about/guidelines.xhtml

and approved of the final manuscript and agreed to be accountable for all aspects of the work.

References

1 World Health Organization, editor. *Monitoring the building blocks of health systems: a handbook of indicators and their measurement strategies*. Geneva: : World Health Organization 2010.

2 Kumar M, Gotz D, Nutley T, *et al.* Research gaps in routine health information system design barriers to data quality and use in low- and middle-income countries: a literature review. *Int J Health Plann Manage* 2018;**33**.

3 Wagenaar BH, Sherr K, Fernandes Q, *et al.* Using routine health information systems for well-designed health evaluations in low- and middle-income countries. *Health Policy Plan* 2016;**31**:129–35. doi:10.1093/heapol/czv029

4 Wagenaar BH, Hirschhorn LR, Henley C, *et al.* Data-driven quality improvement in low- and middle-income country health systems: lessons from seven years of implementation experience across Mozambique, Rwanda, and Zambia. *BMC Health Serv Res* 2017;**17**:830. doi:10.1186/s12913-017-2661-x

5 Hagaman AK, Singh K, Abate M, *et al.* The impacts of quality improvement on maternal and newborn health: preliminary findings from a health system integrated intervention in four Ethiopian regions. *BMC Health Serv Res* 2020;**20**:522. doi:10.1186/s12913-020-05391-3

6 Ouedraogo M, Kurji J, Abebe L, *et al.* A quality assessment of Health Management Information System (HMIS) data for maternal and child health in Jimma Zone, Ethiopia. *PLoS One* 2019;**14**:e0213600. doi:10.1371/journal.pone.0213600

7 Endriyas M, Alano A, Mekonnen E, *et al.* Understanding performance data: health management information system data accuracy in Southern Nations Nationalities and People's Region, Ethiopia. *BMC Health Serv Res* 2019;**19**:175. doi:10.1186/s12913-019-3991-7

8 Ministry of Health, Ethiopia. Health Sector Transformation Plan (HSTP) 2015/16 - 2019/20. 2015.https://www.globalfinancingfacility.org/sites/gff_new/files/Ethiopia-health-system-transformation-plan.pdf (accessed 9 Oct 2019).

9 Dehnavieh R, Haghdoost A, Khosravi A, *et al.* The District Health Information System (DHIS2): A literature review and meta-synthesis of its strengths and operational challenges based on the experiences of 11 countries. *Health Inf Manag* 2019;**48**:62–7. doi:10.1177/1833358318777713

10 Thangasamy P, Gebremichael M, Kebede M, *et al.* A pilot study on District Health Information Software 2: challenges and lessons learned in a developing country: an experience from Ethiopia. *International Research Journal of Engineering and Technology* 2016;**03**:7.

For peer review only - http://bmjopen.bmj.com/site/about/guidelines.xhtml

11 Adane A, Adege TM, Ahmed MM, *et al.* Routine health management information system data in Ethiopia: consistency, trends, and challenges. *Global Health Action* 2021;**14**:1868961. doi:10.1080/16549716.2020.1868961

12 Teklegiorgis K, Tadesse K, Mirutse G, *et al.* Level of data quality from Health Management Information Systems in a resources limited setting and its associated factors, eastern Ethiopia. *S Afr J Inf Manag* 2016;**18**:8. doi:10.4102/sajim.v18i1.612

13 Dagnew E, Woreta SA, Shiferaw AM. Routine health information utilization and associated factors among health care professionals working at public health institution in North Gondar, Northwest Ethiopia. *BMC Health Serv Res* 2018;**18**:685. doi:10.1186/s12913-018-3498-7

14 Shiferaw AM, Zegeye DT, Assefa S, *et al.* Routine health information system utilization and factors associated thereof among health workers at government health institutions in East Gojjam Zone, Northwest Ethiopia. *BMC Med Inform Decis Mak* 2017;**17**:116. doi:10.1186/s12911-017-0509-2

15 Bhattacharya AA, Umar N, Audu A, *et al.* Quality of routine facility data for monitoring priority maternal and newborn indicators in DHIS2: A case study from Gombe State, Nigeria. *PLoS One* 2019;**14**:e0211265. doi:10.1371/journal.pone.0211265

16 Gimbel S, Mwanza M, Nisingizwe MP, *et al.* Improving data quality across 3 sub-Saharan African countries using the Consolidated Framework for Implementation Research (CFIR): results from the African Health Initiative. (Implementation science as an essential driver for sustainable health systems strengthening interventions: Lessons learned across the five-country African Health Initiative.). *BMC Health Serv Res* Published Online First: 2017. doi:10.1186/s12913-017-2660-y

17 Maokola W, Willey BA, Shirima K, *et al.* Enhancing the routine health information system in rural southern Tanzania: successes, challenges and lessons learned. *Trop Med Int Health* 2011;**16**:721–30. doi:10.1111/j.1365-3156.2011.02751.x

18 Aqil A, Lippeveld T, Hozumi D. PRISM framework: a paradigm shift for designing, strengthening and evaluating routine health information systems. *Health Policy Plan* 2009;**24**:217–28. doi:10.1093/heapol/czp010

19 Lemma S, Janson A, Persson L-Å, *et al.* Improving quality and use of routine health information system data in low- and middle-income countries: A scoping review. *PLoS One* 2020;**15**:e0239683. doi:10.1371/journal.pone.0239683

20 World Health Organization. Data Quality Review (DQR) Toolkit. 2017.www.who.int

21 Bosch-Capblanch X, Ronveaux O, Doyle V, *et al.* Accuracy and quality of immunization information systems in forty-one low income countries. *Trop Med Int Health* 2009;**14**:2–10. doi:10.1111/j.1365-3156.2008.02181.x

22 Ohiri K, Ukoha NK, Nwangwu CW, *et al.* An Assessment of Data Availability, Quality, and Use in Malaria Program Decision Making in Nigeria. *Health Syst Reform* 2016;**2**:319–30. doi:10.1080/23288604.2016.1234864

For peer review only - http://bmjopen.bmj.com/site/about/guidelines.xhtml

23 Roomaney A, Pillay-Van Wyk V, Awotiwon OF, *et al.* Availability and quality of routine morbidity data: Review of studies in South Africa. *J Am Med Inform Assoc* Published Online First: April 2017. doi:10.1093/jamia/ocw075

24 Regeru RN, Taegtmeyer M, Kumar M, *et al.* Exploring data quality in community health information systems in Kenya: A mixed method study. *BMC Proceedings* Published Online First: September 2017. doi:10.1186/s12919-017-0074-9

25 Assefa Ayalew G, Molla Teferi B, Mengistu Mitiku A, *et al.* Enhancing health facility-based data quality and use for decision making at primary health care units to improve health service delivery of maternal newborn child and adolescent health, Tigray Ethiopia 2018. *Archives of Community Medicine and Public Health* 2020;**6**:031–5. doi:10.17352/2455-5479.000068

26 Nicol E, Dudley L, Bradshaw D. Assessing the quality of routine data for the prevention of mother-to-child transmission of HIV: An analytical observational study in two health districts with high HIV prevalence in South Africa. *Int J Med Inform* 2016;**95**:60–70. doi:10.1016/j.ijmedinf.2016.09.006

27 Mutale W, Chintu N, Amoroso C, *et al.* Improving health information systems for decision making across five sub-Saharan African countries: Implementation strategies from the African Health Initiative. *BMC Health Serv Res* 2013;**1**:S9. doi:10.1186/1472-6963-13-S2-S9

28 Lazzerini M, Senanayake H, Mohamed R, *et al.* Implementation of an individual patient prospective database of hospital births in Sri Lanka and its use for improving quality of care. *BMJ OPEN* 2019;**9**. doi:10.1136/bmjopen-2018-023706

29 Ndira SP, Rosenberger KD, Wetter T. Assessment of Data Quality of and Staff Satisfaction with an Electronic Health Record System in a Developing Country (Uganda): A Qualitative and Quantitative Comparative Study. *Methods Inf Med* 2008;**47**:489–98. doi:10.3414/ME0511

30 Gebreslassie AA, Below MT, Ashebir MM, *et al.* Enhancing health facility-based data quality and use for decision making at primary health care units to improve health service delivery of maternal newborn child and adolescent health, Tigray Ethiopia 2018. *Arch Community Med Public Health* 2020;**6**:031–5.

31 Nicol E, Hanmer LA. Routine Health Information Systems in South Africa--Opportunities for Improvement. *Stud Health Technol Inform* 2015;**1**:993.

32 Gumede-Moyo S, Todd J, Bond V, *et al.* A qualitative inquiry into implementing an electronic health record system (SmartCare) for prevention of mother-to-child transmission data in Zambia: a retrospective study. *BMJ Open* 2019;**9**:e030428. doi:10.1136/bmjopen-2019-030428

33 Yourkavitch J, Zalisk K, Prosnitz D, *et al.* How do we know? An assessment of integrated community case management data quality in four districts of Malawi. *Health Policy Plan* 2016;**31**:1162–71. doi:10.1093/heapol/czw047

For peer review only

For peer review only - http://bmjopen.bmj.com/site/about/guidelines.xhtml

34 Khatri N, Brown GD, Hicks LL. From a blame culture to a just culture in health care. [Review] [70 refs]. *Health Care Management Review* 2009;**34**:312–22. doi:10.1097/HMR.0b013e3181a3b709

35 Braa J, Heywood A, Sahay S. Improving quality and use of data through data-use workshops: Zanzibar, United Republic of Tanzania. (Special issue: e-health.). *Bull World Health Organ* 2012;**90**:379–84. doi:10.2471/BLT.11.99580

For peer review only

For peer review only - http://bmjopen.bmj.com/site/about/guidelines.xhtml

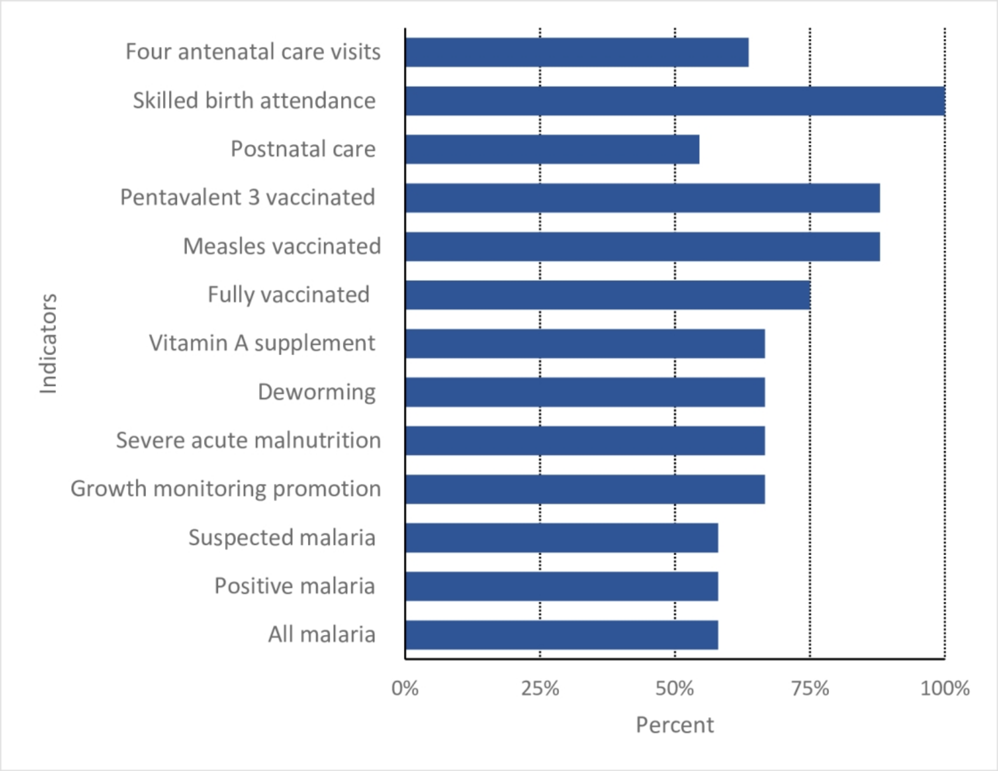

Figure 1 Availability of source documents and reports for the facility-months observed

140x108mm (300 x 300 DPI)

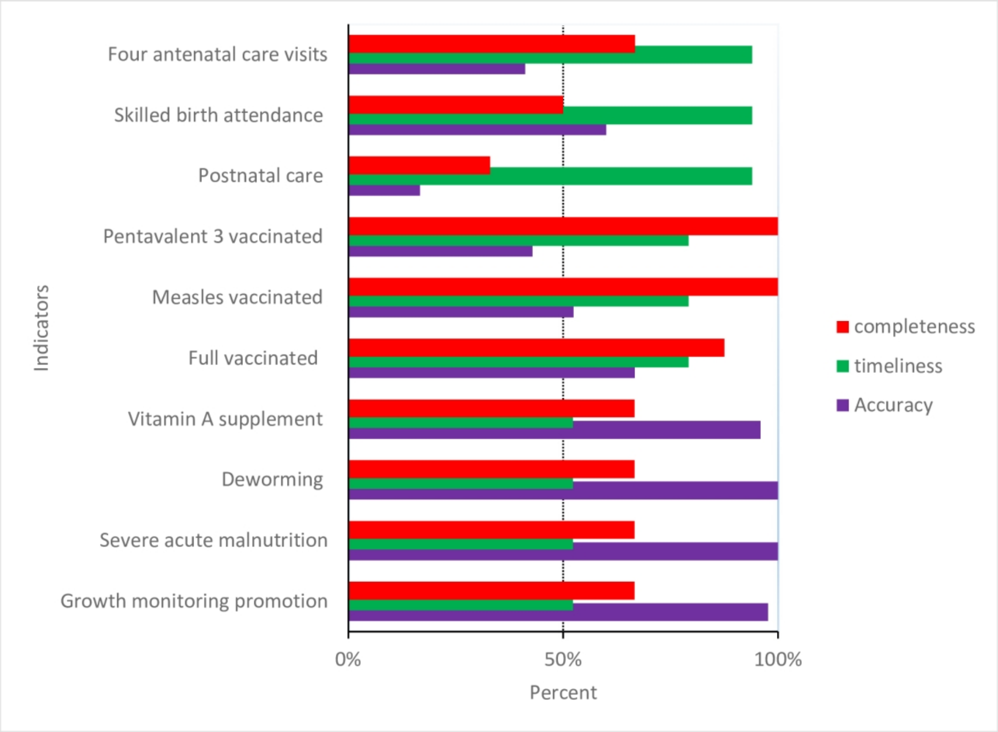

Figure 2 Completeness, timeliness and accuracy of reporting for selected indicators in the routine health information system

162x119mm (300 x 300 DPI)

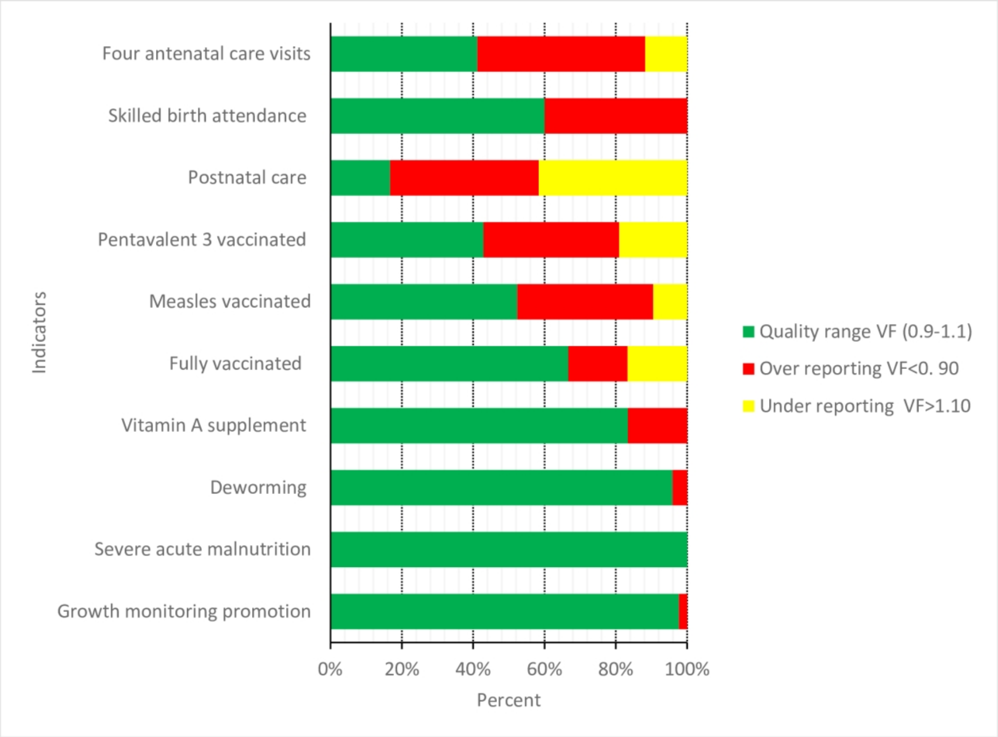

Figure 3 Accuracy of reporting for selected indicators in the routine health information system

171x126mm (300 x 300 DPI)

Immunization team: Data collection tool

I. Quantitative data quality assessment checklist to be used at Woreda health office, Hospital, Health Center and Health Post levels

Data collection Site/level; woreda/ hospital/ health centre/health post ____________ Date of data collection______________________			
S.N	Description	Response (Yes/No). Note every 'yes' answer will score '1' and '0' for answer 'No'. If not applicable, NA	Comments/clarifications
**A. Demographic/planning**			
1.	Is there a target number of children that the woreda or health facility strives to vaccinate a calendar year or reporting period?	1. Yes<br>2. No<br>3. NA	Obtain the target value and previous review period's annual total. Even if the target is unrealistically high/low, as long as they have set a target they score 1
2.	***<u>Ask this question at woreda health office only</u>***<br>Is the denominator value (for infant immunization) found at the woreda level the same as the one found at the national level?	1. Yes<br>2. No<br>3. NA<br>Denominator set at national ________	Refer in the record in the log book.
3.	Is the proportion of infants for pentavalent3, measles and fully vaccination type known for the woreda and service delivery points	1. Yes<br>2. No<br>3. NA	Strategy; fixed, outreach, mobile. A proportion (%) by each antigen should be available and known
4.	Is there an up-to-date plan for:<br>• The woreda and<br>• The health delivery point (current review period)	1. Yes<br>2. No<br>3. NA	Plan should include the planning process to increase routine vaccinated coverage. It may be integrated with other health services<br>*Please review reference document*
5.	Is there a woreda and health facilities map of catchment areas (including outreach sites) showing health facility providing immunization strategy	1. Yes<br>2. No<br>3. NA	The map should include denominator, target, strategy type
**B. Monitoring and evaluation**<br>Dear participant, now we are going to see the monitoring and evaluation component of immunization data. Thank you for your time**.**			

For peer review only - http://bmjopen.bmj.com/site/about/guidelines.xhtml

6.	Is there an up-to-date chart/table of the current review period`s vaccinated coverage displayed any where	1. Yes<br>2. No<br>3. NA	The chart should be displayed in a visible site
7.	Is the completeness of the immunization reports monitored at each reporting level? (woreda and health facilities)	1. Yes<br>2. No<br>3. NA	Refer document for evidence
8.	Does the woreda and health facility monitor reporting timelines for health facility immunization reporting	1. Yes<br>2. No<br>3. NA	Refer document for evidence
9.	Is there an up to date data monitoring of the current review period`s immunization dropout rates?	1. Yes<br>2. No<br>3. NA	Refer document for evidence<br>Can be on the same chart or table as coverage. but score 1 if the health worker can tell you the dropout rate of his health facility
10.	**Ask this question for woreda and health centre only**<br>Is there a routine feedback format for the next lower level	1. Yes<br>2. No<br>3. NA	Refer document for evidence<br>Feedback mean written summary or analysis of immunization data (woreda to health centres, health centers to health posts).<br>Routine means regular feedbacks on monthly bases (not ad hoc). Format means written if distributed or if in from of a meeting is minutes of meeting
11.	Are there regular meetings with health unit workers to discuss immunization performance	1. Yes<br>2. No<br>3. NA	Refer document for evidence<br>Such meetings should occur outside supervisions and involve workers from several health units<br>If yes, how often is this meeting happening???
12.	Are there designated staffs responsible for reviewing the quality of data (i.e., accuracy, completeness and timeliness) received from sub-reporting levels (e.g., service points)?.	1. Yes<br>2. No<br>3. NA	Designated staffs can be PMT or HMIS focal assigned to do this task

For peer review only - http://bmjopen.bmj.com/site/about/guidelines.xhtml

13.	Does all designated staff have received training on the data management processes and tools?	1. Yes<br>2. No<br>3. NA	
14.	**Ask this question for woreda and health centre only**<br>Are supervision activities are conducted weekly, monthly or quarterly?	1. Yes<br>2. No<br>3. NA	Refer document for evidence.<br>A written schedule of supervision that includes visiting every health unit with as specified period of time. Supervision must include immunization, record should include list of health facilities, date visited and by whom. Check core indicator
15.	Is there a mechanism of monitoring vaccine stock outs at of health facility level?	1. Yes<br>2. No<br>3. NA	The manager should able to say (based on written information) weather one health unit has encountered a vaccine stock out. If no vaccine stock out reported, ensure that the monitoring is possible and done. Stock out means interruption in vaccine supply (for any vaccine)
**C. Recording practices**<br>Dear volunteer, now we are going to switch the discussions to immunization data recording practices.			
16.	Are vaccines receipt and issues recorded in vaccine ledger book	1. Yes<br>2. No<br>3. NA	If no Ledger book, or inappropriate record keeping of vaccine receipts and issues, score 'No'. If it is not sufficient to have bin card, but an appropriate carder system will suffice provided it is correctly maintained, stored and archived
17.	Is the current ledger book up to date for all vaccines reviewed (including dry supplies)	1. Yes<br>2. No<br>3. NA	Refer document for evidence
18.	Were immunization forms (tally sheets, reporting formats, vaccine requisition formats) sufficiently available in the visited health units during the review period	1. Yes<br>2. No<br>3. NA	Check the availability of formats. If one of the format is missing, score 'zero'

For peer review only - http://bmjopen.bmj.com/site/about/guidelines.xhtml

19.	***<u>Ask this question at service delivery level only</u>***<br>Are there tally sheets for infant vaccinations on the desk (or easily available)?	1. Yes<br>2. No<br>3. NA	Check the availability of the tally sheet. If tally sheets completed by month and not each immunization session, ensure that tally sheet has month/review period clearly marked. Check the numbers given during the last immunization session
20.	Do tally sheets have entries for the last immunization day?	1. Yes<br>2. No<br>3. NA	<u>Review the document</u>
21.	Did the individual reporting and recording form (tally sheets) from the respective health units use the same form/format for the current review period	1. Yes<br>2. No<br>3. NA	If there was an official change in the report format, a four month flexibility should be allowed (mix of old and new forms for a maximum of 4 months)
22.	***<u>Ask this question at service delivery level only</u>***<br>Are registers (or pre-printed forms) used for recording individual information about child immunization	1. Yes<br>2. No<br>3. NA	Check the child registers. These may be child health cards, if cards are kept in health facility
23.	***<u>Ask this question at service delivery level only</u>***<br>Can a child`s vaccination history be easily and rapidly retrieved in the registers	1. Yes<br>2. No<br>3. NA	Review the document. A new dose should not be entered as a complete new entry but entered in the location where previous doses have been entered. Score 0 if register is used as a new entry for any immunization
24.	***<u>Ask this question at service delivery level only</u>***<br>Was the correct individual record completed for every vaccination observed	1. Yes<br>2. No<br>3. NA	Observe immunization of five (among infants) and check the child card. If no observation score, NA. If you can observe five immunizations, then skip child health card exercise.
25.	***<u>Ask this question at service delivery level only</u>***<br>Was the correct date to return given for every vaccination observed	1. Yes<br>2. No<br>3. NA	Observe immunization of five (among infants) and check the child card. If no observation score, NA.

For peer review only - http://bmjopen.bmj.com/site/about/guidelines.xhtml

26.	Does the woreda office and health facility stamp or write the date of report from health facility is received at woreda level on the report	1. Yes<br>2. No<br>3. NA	Needs to be answered as such by the woreda medical officer or person and health facility manager in charge of immunization services (i.e., is the monitoring of timeliness systematic and organized). This must be the first date, the report was seen (received) at woreda or health facility level, not the date the report was processed
**D. Storing/Reporting Practices**<br>Dear respondent, we are now going to discuss on immunization data storing and reporting process			
27.	Have all available health facility reports from the period previous to the last one been processed	1. Yes<br>2. No<br>3. NA	Processing means, entering data in to whatever system they use (electronic or paper). Ideally there should be a written instructions about the process and procedures
28.	Are all the health facility reports available for the entire review period	1. Yes<br>2. No<br>3. NA	Monthly or quarterly depending on country. Record in the logbook all the dates that are missing.
29.	Is there a procedure/ system of dealing with late reporting	1. Yes<br>2. No<br>3. NA	Information coming late should be sent to the national level. The explanation provided should be in line with national guidelines. If no national guideline, the system should be functional.
30.	Does each health facility have its own file or sub file?	1. Yes<br>2. No<br>3. NA	Review the document<br>Storage should facilitate retrieval and monitoring (and be well organized). If the storage is filled by date only (not by health centre) score yes if any report search is easy

For peer review only - http://bmjopen.bmj.com/site/about/guidelines.xhtml

31.	Does each health facility papers files are filed by date?	1. Yes<br>2. No<br>3. NA	Review the document
32.	Is there a written back up procedure for every reporting to the next level?	1. Yes<br>2. No<br>3. NA	If not computerized score 'na'
33.	Can the official immunization tabulation for the review period be reproduced from an archive electronic file?	1. Yes<br>2. No<br>3. NA	If not computerized score 'na' official immunization tabulation means final summary of review period data. Archive electronic file means stored file from hard disk/diskettes
34.	Is the data of printing/production on every tabulation/chart or, if the data is archived, the date the archive file was created?	1. Yes<br>2. No<br>3. NA	If not computerized score 'na'

II. Data verifications at the woreda health office and service delivery points

A. Immunization service data comparison between reports and records

Date ____________________ Time _________________________ Health institution code ___________

Description	Immunization data reports and records in the review period								
	Pentavalent 3			Measles			Fully vaccinated		
Recount results from the periodic reports sent from service sites to the Woreda and compare to the value reported by the Woreda.	Month 1	Month 2	Month 3	Month 1	Month 2	Month 3	Month 1	Month 2	Month 3
Reported by the health centre/hospital (By EPI unit)									
Recounted health center/hospital (tally sheet)									
Recounted health center/hospital (registration book)									
Ratio of reported over recorded									

For peer review only - http://bmjopen.bmj.com/site/about/guidelines.xhtml

Aggregated PHCU reported to woreda (including health posts)									
Reported by health post to health centre									
Recounted health post (tally sheet)									
Recounted health post (registration book)									
Ratio reported over recorded									

B. Immunization logistics data reports and records

Date ______________________ Time _____________________ Health institution code ___________________

Antigen	Logistics data in the review period											
	Month 1				Month 2				Month 3			
	Beginning balance	Quantity received	Loss/ adj	End-ing balance	Beginning balance	Quantity received	Loss/ adj	Ending balance	Beginning balance	Quantity received	Loss/ adj	End-ing balance
Pentavalent												
PCV												
Rota												
BCG												
IPV												
BOPV												
Measles												

C. Immunization data reports timeliness and completeness check list*

Date _________________________________ Time _____________________________ Health institution code __________

Health facilities	Reporting period			Agreed reporting time line	Total completeness	Total timeliness
	Month 1/date of the report received	Month 2/date of the report received	Month 3/date of the report received			
Health centre 1						
Health centre 2						

For peer review only - http://bmjopen.bmj.com/site/about/guidelines.xhtml

Health centre 3						
Health centre 4						
Health post 1						
Health post 2						
Health post 3						
Health post 4						
Health post 5						
Hospital						
Total received this month (NO)						
Total received this month (%)						
Cumulative completeness (%)						
Total on time this month (NO)						
Cumulative timeliness (%)						

*Adapted from the immunization data quality self-assessment (DQS) tool (WHO, 2005)

Key

- Insert the date the health facility reports were received at the health institution. If a report is received after the deadline, enter the date in red.
- Total completeness or timeliness: refers to the reporting completeness of the health facility. Cumulative completeness: reports received up to that month divided by reports expected up to that month.

  Cumulative timeliness: reports received on time up to that month divided by reports expected up to that month.

For peer review only

For peer review only - http://bmjopen.bmj.com/site/about/guidelines.xhtml

Data collection tool for malaria indicators
Structured questionnaire for health facility

**CONFIRMED AND SUSPECTED MALARIA CASES**				
1	Does this facility diagnose and treat malaria?	1. Yes ☐	0. No ☐	
**SOURCE DOCUMENTS AND REPORTS**				
2	Does this facility report malaria data to a reporting system?	1. Yes ☐	0. No ☐	
3	To which of the following reporting systems does the facility report malaria data?	1. Health Management Information System (HMIS)<br>2. Public health Emergency Management (PHEM)<br>3. Malaria program<br>4. Nongovernmental organizations or institutions<br>5. Other reporting system (Specify)		
**4**	What is the source document used by this facility for monthly/weekly reporting of malaria?	1. Laboratory register<br>2. OPD register<br>3. Inpatient register<br>4. Emergency register<br>5. Other (specify)		
**REPORT TIMELINESS**				
**5**	Is there a deadline for submission of the malaria report by the health facilities?	1. Yes<br>2. No		
**6**	If yes, what is the deadline (date of month)?<br>(Write the end date of the deadline)	Reporting deadline:____________________		
**7**	Does the health facility record the dates of submission of monthly/weekly malaria reports to the Woreda/Zone/Region (see logbook/computer)?	1. Yes<br>2. No		
**IF AVAILABLE, REVIEW THE RECORDS AND CHECK THE DATES OF SUBMISSION FOR THE THREE REVIEW MONTHS**				
**8**	Month 1	Reported date __________		
	Month 2	Reported date__________		
	Month 3	Reported date ___________		
**9**	If any discrepancy is observed between Reporting deadline and each reporting date, what are the possible reason?	1. Shortage of man power<br>2. Interruption of electricity/computer<br>3. Shortage of reporting formats<br>4. Competing priority (Campaign)<br>5. Reporting date aligned with holiday<br>6. Other (Specify)		If there is no discrepancy skip to Q10
**10**	What method of reporting system does the facility use	1. Paper based system<br>2. Electronic system		

		3. Both	
**DATA USE FOR DECISION MAKING**			
**11**	Does the health facility have analysed malaria data (e.g., summary tables, charts, maps)?	0. No<br>1. Yes, observed paper-based<br>2. Yes, observed electronic<br>3. Both	
**12**	Does the health facility uses analysed malaria data for decision making	1. Yes<br>2. No	If no Skip to Q14
**13**	If Yes, for what purpose	1. Performance management (Planning and reporting)<br>2. Supply requesting and reporting<br>3. Priority setting<br>4. Monitoring targets<br>5. Advocacy<br>6. Other (Specify)	Skip to Q16
**14**	If no, what are the possible bases for decision making in your health facility	1. Personal preference for decision making<br>2. Superior directives<br>3. What was done in last year<br>4. Funding directives from higher level.<br>5. Political considerations<br>Other (Specify)	
**15**	If No, what are the possible reasons for not using malaria data for decision making in your health facility	1. Poor data quality<br>2. Unavailability of data<br>3. Negative Perception<br>4. Other (Specify)	

**REVIEW THE SOURCE DOCUMENTS AND MONTHLY REPORT FOR SUSPECTED MALARIA CASE**				
**1**	Please confirm the availability of **source documents** for malaria for month 1 to month 3. If available, please **Recount** the number of **Suspected malaria cases** recorded in the **source document** month 1 to month 3.	**(A) Source documents available**	**(B) Recount the number of Suspected malaria cases in the source documents (if none, please enter 0)**	**(C)Record monthly Suspected malaria cases from monthly report**

	Months	Yes, available and complete*	Yes, available but partly** complete	Yes, available but no data recorded	No		
01	**Month 1**	1	2	3	0		
02	**Month 2**	1	2	3	0		
03	**Month 3**	1	2	3	0		
**REVIEW THE SOURCE DOCUMENTS AND MONTHLY REPORT FOR CONFIRMED MALARIA CASE**							
**2**	Please confirm the availability of **source documents** for malaria for month 1 to month 3. If available, please **Recount** the number of conf. malaria cases recorded in the **source document** month 1 to month 3.	**(A) Source documents available**				**(B) Recount the number of conf. malaria cases in the source documents (if none, please enter 0)**	**(C)Record monthly confirmed malaria cases from monthly report**
	**Months**	**Yes, available and complete***	**Yes, available but partly** complete**	**Yes, available but no data recorded**	**No**		
	**Month 1**	1	2	3	0		
	**Month 2**	1	2	3	0		
	**Month 3**	1			0		
**REVIEW THE SOURCE DOCUMENTS AND MONTHLY REPORT FOR TOTAL MALARIA CASE**							
**1**	Please confirm the availability of **source documents** for malaria for month 1 to month 3. If available, please **Recount** the number of **total malaria**	**(A) Source documents available**				**(B) Recount the number of total malaria cases in the source documents (if none, please enter 0)**	**(C)Record monthly total malaria cases from monthly report**

For peer review only

	**cases** recorded in the **source document** month 1 to month 3.						
	**Months**	**Yes, available and complete***	**Yes, available but partly** complete**	**Yes, available but no data recorded**	**No**		
	**Month 1**	1	2	3	0		
	**Month 2**	1	2	3	0		
	**Month 3**	1	2		0		
**Take the last 15 entries recorded in the Lab register for each reporting period and check if all the data elements relevant to the selected indicator are filled in.<br>*COMPLETE means that the source document contains the data relevant to the selected indicator.<br>**PARTLY: the register is available but some information is missing.**							
2	If the source document (Lab register) is not available, what are the possible reasons?	1. Storage or archiving problems<br>2. Absence of designated staff<br>3. Stock out of source document<br>4. Other (specify): ___					
3	If the source documents (Lab register/) are partially complete or has no data, what are the possible reasons for the missing data?	1. Staffing issue(s)(shortage, absence)<br>2. Not understanding the data element<br>3. Presence of other vertical reporting requirement<br>4. Data burden (too much data elements to be recorded)<br>5. The recording tool is not designed as user friendly<br>6. Other (specify): ___					
**DATA COMPLETENESS**							

For peer review only

5	If the monthly report for malaria is not available, what are the possible reasons?	1. Storage or archiving problems<br>2. Absence of designated staff<br>3. Stock out of source document<br>4. Other (specify): ___________________________
6	If the monthly report of malaria is partially complete or has no data, what are the possible reasons for the missing data?	1. Staffing issue(s)(shortage, absence)<br>2. Not understanding the data element<br>3. Presence of other vertical reporting requirement<br>4. Data burden (too much data elements to be recorded)<br>5. The recording tool is not designed as user friendly<br>6. There is no client to be reported<br>7. Other (specify): _________________________________
**DISCREPANCIES**		
**7**	If there was a discrepancy observed between the **source document** and the **monthly report**, what are the reasons for the discrepancy?	1. Data entry errors<br>2. Arithmetic errors<br>3. Information from all source documents not compiled correctly<br>4. Data burden (too much data elements to be reported)<br>5. Illegible writing on the source document (not readable)<br>6. Lack of emphasis for data accuracy<br>7. Other (specify) _________________________
**8**	If there was a discrepancy observed between the **source document** and the **monthly report**, what are the reasons for the discrepancy?	1. Data entry errors<br>2. Arithmetic errors<br>3. Information from all source documents not compiled correctly<br>4. Data burden (too much data elements to be reported)<br>5. Illegible writing on the source document (not readable)<br>6. Lack of emphasis for data accuracy<br>7. Other (specify) _________________________

For peer review only

Data collection tool for Maternal Health Indicators

Checklists for collecting data on quality at woreda and health facility level, adapted from USAID RDQA Measure Evaluation

This questionnaire will be used to collect data from the health facilities and woreda health offices in order to assess the data quality and data management system.

Facility Identification

Interviewer Name: ___________________________________

Full reference of report (name, year, publisher etc.)			
Type of Document			
Study Design			
Study area/s			
Study period			
Study population			
Sample size			
Indicators	Definition (Numerator /Denominator)	Reported Coverage	
		National	Benishangul Gumz Region
Antenatal care visits at least one (ANC1)			
Antenatal care 4th visit (ANC4)			
Skilled delivery attendance			
Postnatal Care			
Comments			

Number	Question	Result	
SECTION 1: COVER PAGE			
Date	DAY______________<br>Day	MONTH____________<br>Month	YEAR________<br>Year
FACILITY IDENTIFICATION			
Q001	Region Name		
Q002	Zone/sub-city name		
Q003	Facility ID		
Q004	Official name of facility		

Number	Question	Result	
Q005	Woreda Name		
Q006	Town Name		
Q007	Kebele Name		
Q008	Type of facility	HEALTH CENTRE<br>HEALTH POST	1<br>2
Q009	Urban/Rural	URBAN<br>RURAL	1<br>2

1. Service Delivery Sites (health facilities)

Demographic data			
	Information	Response Yes=1,No=2	Remark
1	Does the facility have the catchment population the calendar year?		If *'yes'* how much …
2	Does the health center know the total number of eligible mothers for maternal service to be provided in the calendar year?		
3	Is the denominator value (for ANC, delivery and PNC) found in the health center level the same as the one found in the national level		

A - Documentation Review:						
Review availability and completeness of all indicator source documents for the selected reporting period *(if all necessary documents/facility reports are available 1=Yes, if all the necessary documents/facility reports are not available, 2= No otherwise the answer will be 3=Yes partly*			ANC1	ANC4	SBA	EPNC
SDS_1A	Review available data sources for the reporting period being verified. Are all necessary data sources (registers tally and reports) available for review?					
	Tikmit 2010/Octo 2017	Tally				
		Register				
		Report from the lower level				
	Hidar 2010/Nov 2017	Tally				
		Register				
		Report from the lower level				
	Tahisas 2010/ Dec 2017	Tally				
		Register				
		Report from the lower level				
SDS_2A	Are all available data sources complete? (Register and/or tally sheet for 2nd quarter of 2010 E.C)					

	Tikmit 2010/Octo 2017	Tally				
		Register				
		Report from the lower level				
	Hidar 2010/Nov 2017	Tally				
		Register				
		Report from the lower level				
	Tahisas 2010/ Dec 2017	Tally				
		Register				
		Report from the lower level				
SDS_3A	Review the dates on the data sources. Do all dates fall within the 2nd quarter of 2010E.C? (*Yes=1, No=2*)					
	Tikmit 2010/October 2017					
	Hidar 2010/Nov 2017					
	Tahisas 2010/ Dec 2017					
SDS_4A	If the source document (service delivery register) is not available, what are the possible reasons?					
	1. Storage or archiving problems					
	2. Stock out of source document					
	3. Absence of designated staff					
	4. Other (specify)					
SDS_5A	If the source document (service delivery register) is not completely filled in, what are the possible reasons for the missing data?					
	1. Staffing issue(s) (shortage, absence)					
	2. Not understanding the data element					
	3. Presence of other vertical reporting requirement					
	4. Data burden (too much data elements to be recorded)					
	5. The recording tool is not designed as user friendly					
	6. other (specify):					

B. REPORT TIMELINESS				
SDS_1B	Does the health facility record the dates of submission of monthly HMIS reports to the Health center/Woreda (see logbook/computer)?	1. Yes ☐	0. No	
SDS_2B	If '*Yes*' for SDS_1`B, review the records and check the dates of submission for the three review months			
		Tikmit 2010/Octo	Hidar 2010/Nov	Tahisas 2010/ Dec 2017

For peer review only

	Were the HMIS monthly reports submitted on time? *(Current practices is submission from 21th to 26th of the month)*	1. Yes ☐<br>0. No ☐	1. Yes ☐<br>0. No ☐	1. Yes ☐<br>0. No ☐	
C- Recounting reported Results (accuracy)					
Recount results from source documents, compare the verified numbers to the site reported numbers and explain discrepancies(if any)		ANC 1	ANC4	SBA	EPNC
SDS_1C	Recount the number of people, cases or events during the reporting period by reviewing the data source (register HC)/tally HP) (A).				
	Tikmit 2010/October 2017				
	Hidar 2010/Nov 2017				
	Tahisas 2010/ Dec 2017				
SDS_2C	Enter number of people, cases or events reported by the site during reporting period from site summary report(B)				
	Tikmit 2010/October 2017				
	Hidar 2010/Nov 2017				
	Tahisas 2010/ Dec 2017				
SDS_4C	Calculate the ratio of recounted to reported (A/B)				
	Tikmit 2010/October 2017				
	Hidar 2010/Nov 2017				
	Tahisas 2010/ Dec 2017				
SDS_5C	What are the reasons for discrepancy (if any) observed ((i.e. data entry errors, missing data sources, others)?				
	1. Storage or archiving problems				
	2. Absence of designated staff				
	3. Stock out of source document				
	4. other(specify)				

For peer review only

D. Systems Assessment		
I - M&E Structure, Functions and Capabilities (1.Yes completely 2. Partly 3. No 4.NA		
SDS_1D	Is there a designated staff responsible for reviewing aggregated numbers prior to submission to the next level? (e.g. to health center or wereda)	
SDS_2D	Have all relevant staff has received training on the data management processes and tools.	
The M&E unit has provided written guidelines to each sub-reporting level on (*Yes=1, No=2*) *NB: Ask and observe the available guidelines*		
SDS-3D	What they are supposed to report on?	
SDS_4D	How (e.g. in what specific format) reports are to be submitted?	
SDS_5D	To whom the reports should be submitted?	
SDS_6D	When the reports are due?	
III - Data-collection and Reporting Forms and Tools (Yes=1, No=2)		
SDS_7D	Have there been clear instruction/orientation given to relevant staffs on how to complete the data collection and reporting forms/tools.	
SDS_8D	Is the supply of standard reporting forms/tools consistent in the facility?	
SDS_9D	Do the M&E Unit monitor on the consistent utilization of standard reporting forms/tools at all reporting levels.	
SDS_10D	The standard forms/tools are consistently used by the Service Delivery Site.	
SDS_11D	Are all *source documents* and *reporting forms* relevant for measuring the indicator (s) are available (hard copy print outs) for auditing purposes. *(Including dated print-outs in case of computerized system)*	
IV- Data Management Processes *(Yes=1, No=2) This section is only for Health Centers*		
SDS_12D	If applicable, there are quality controls in place for when data from paper-based forms are entered into a computer (e.g. double entry, post-data entry verification, etc). *(if not computerized skip to SDS_15D)*	
SDS_13D	If applicable, there is a written back-up procedure for when data entry or data processing is computerized.	
SDS_14D	*….if 'yes' for SDS_12D* the latest date of back-up is appropriate given the frequency of update of the computerized system (e.g., back-ups are weekly or monthly).	
SDS_15D	The recording and reporting system avoids double counting people *within* and *across* Service Delivery Points (e.g., a person receiving the same service twice in a reporting period, a person registered as receiving the same service in two different locations, etc).	

V - Links with National Reporting System *(Yes=1, No=2)*		
SDS_16D	The relevant national forms/tools are used for data-collection and reporting.	
SDS_17D	Data are reported through a single channel of the national information systems.	
VI – Use of Data for decision making *(Yes=1, No=2)*		
SDS_18D	The service delivery site develops charts, graphs, maps, etc …(If yes, ask to see them)	
SDS_19D	Staff at the health facility has access to guideline/technical assistance on data use (e.g. peer review meetings or during supervisory visits)	
SDS_20D	The analyzed data/results are presented/ disseminated to stakeholders in a timely manner so that the information can be used for informed decisions. (observe examples)	
SDS_21D	There are programmatic decision taken by the facility based on the analyzed data (see/observe examples)	

2. Woreda level assessment Questions

Demographic data			
	Information	Response Yes=1,No=2	Remark
1	Does the woreda health office have the catchment population the calendar year?		If *'yes'* how much …
2	Does the woreda health office know the total number of eligible mothers for maternal service to be provided in the calendar year?		
3	Is the denominator value (for ANC, delivery and PNC) found in the woreda the same as the one found in the national level? (*If not the same explain the difference)*		

A. Recounting reported results (accuracy)					
Recount results from the periodic reports sent from service sites to the Woreda and compare to the value reported by the District. Explain discrepancies (if any)		ANC1	ANC4	SBA	Early PNC
WLA_1A	Re-aggregate the numbers from the reports received from all Service Delivery Sites. What is the re-aggregated number? [A]				
WLA_1A_a	Tikmit 2010 E.C / Oct. 2017				

WLA_1A_b	Hidar 2010 E.C / Oct. 2017				
WLA_1A_c	Tahisas 2010 E.C / Oct. 2017				
WLA_2A	What aggregated result was contained in the summary report prepared by the Woreda (and submitted to the next reporting level)? [B]				
WLA_2A_a	Tikmit 2010 E.C / Oct. 2017				
WLA_2A_b	Hidar 2010 E.C / Oct. 2017				
WLA_2A_c	Tahisas 2010 E.C / Oct. 2017				
WLA_3A	Calculate the ratio of recounted to reported results. [A/B]				
WLA_3A_a	Tikmit 2010 E.C / Oct. 2017				
WLA_3A_b	Hidar 2010 E.C / Oct. 2017				
WLA_3A_c	Tahisas 2010 E.C / Oct. 2017				
WLA_4A	What are the reasons for the discrepancy (if any) observed<br>1. data entry errors,<br>2. arithmetic errors,<br>3. missing data source,<br>4. (Other please specify)?				

B. Report performance

	Report completeness	
WLA_5A	How many facilities were expected to report [A]	
	Tikmit 2010 E.C / Oct. 2017	
	Hidar 2010 E.C / Oct. 2017	
	Tahisas 2010 E.C / Oct. 2017	
WLA_6A	How many facilities actually reported [B]	
	Tikmit 2010 E.C / Oct. 2017	
	Hidar 2010 E.C / Oct. 2017	
	Tahisas 2010 E.C / Oct. 2017	
WLA_7A	Calculate % Available Reports [B/A] sum	
	Tikmit 2010 E.C / Oct. 2017	
	Hidar 2010 E.C / Oct. 2017	
	Tahisas 2010 E.C / Oct. 2017	
WLA_8A	Check the dates on the reports received. How many reports were received on time? (i.e., received by the due date). [C]	

For peer review only

WLA_9A	Calculate % On time Reports [C/B]				
WLA_10A	How many reports were complete? (i.e., complete means that the report contained all the required indicator data, the date of reception of the report by the District, and the authentication by the responsible staff). [D]				
WLA_11A	Calculate % Complete Reports [D/B]				
WLA_12A	If any monthly HMIS reports were not complete, what are the possible reasons for the missing data? *(Multiple responses is possible)* *1.* Staffing issue(s) (e.g. staff shortage, absence of designated staff, etc) 2. Not understanding the data element(s) 3.Presence of other vertical reporting requirements 4.Data burden (too much data elements to be recorded) 5.The design of the reporting form is not user friendly 6.Other (specify)				
	Report timeliness				
WLA_13A	Does the Woreda office record receipt dates of monthly HMIS reports (observe logbook/electronic system)?	1. Yes ☐	0. No ☐	If No, skip to WLA_15A	
WLA_14A	If WLA_13A is 'yes', check the receipt dates for the three review months. How many reports were received on or before the 26th of the month	Health facility type	Tikmit 2010/Oct.2017	Hidar 2010/Nov. 2017	Tahisas 2010 / Dec.2017
		Health centre			
		Health post			
WLA_15A	Does the Woreda office keep a record of its submission of monthly aggregated HMIS reports to Zonal or regional offices (e.g. emails, stamps, receipts, log book, etc.)?	1. Yes ☐	0. No ☐		
WLA_16A	If WLA_16A is yes, check the submission dates (date from---) of the aggregate HMIS reports for the three review months.	Tikimt 2010/Oct 2017<br>1. Yes ☐0. No ☐	Hidar 2010/Nov.20171. Yes ☐0. No ☐	Tahisas 2010 / Dec.2017<br>1. Yes.<br>0. No	
	Part 2. Systems Assessment (Woreda)				
I - M&E Structure, Functions and Capabilities<br>*(1. Yes completely 2. Yes partly 3. No 4. NA)*					
SAW_1A	Are there designated staffs responsible for reviewing the quality of data (i.e., accuracy, completeness and timeliness) received from sub-reporting levels (e.g., service points)?				

SAW_2A	Are there designated staff responsible for reviewing aggregated numbers prior to submission to the next level (e.g., to the central M&E Unit).	
SAW_3A	Does all relevant staff have received training on the data management processes and tools?	
II DATA QUALITY ASSESSMENT MECHANISMS		
SAW_4	Has the Woreda conducted data quality assessments at all health facilities in the review three months? (Please observe)	0. No RDQA conducted<br>1. Yes, RDQA has been conducted in all health posts, Health centers, and hospitals<br>2. RDQA has been conducted, but only in some facilities
SAW_5	Does the Woreda use data quality assessment tools (e.g., RDQA/data verification, in-built electronic data quality validation rules/system)? (Please observe)	0. No ☐<br>1. Yes, observed ☐<br>2. Yes, not observed
SAW_6	Does the Woreda maintain a record of health facility data quality assessments conducted in the past three months? (Please observe)	0. No ☐<br>1. Yes, observed ☐<br>2. Yes, not observed
SAW_7	Does the Woreda maintain records of feedback to health facilities on data quality assessment findings? (Please observe)	0. No ☐<br>1. Yes, observed ☐<br>2. Yes, not observed
III- Indicator Definitions and Reporting Guidelines		
	The M&E Unit has provided written guidelines to each sub-reporting level on:- *(Yes=1, N0=2)*	
WID_1	What they are supposed to report on.	
WID_2	How (e.g., in what specific format) reports are to be submitted.	
WID_3	To whom the reports should be submitted.	
WID_4	To When the reports are due.	
IV - Data-collection and Reporting Forms / Tools *(Yes=1, No=2)*		
WDC_1	Clear instructions have been provided by the M&E Unit on how to complete the data collection and reporting forms/tools.	
WDC_2	The M&E Unit has identified standard reporting forms/tools to be used by all reporting levels	
WDC_3	The standard forms/tools are consistently used by the Service Delivery Site.	
WDC_4	All source documents and reporting forms relevant for measuring the indicator(s) are available (printed out backups) for auditing purposes (including dated print-outs in case of computerized system).	
V- Data Management Processes *(Yes=1, No=2)*		

WDM_1	Feedback is systematically provided to all service points on the quality of their reporting (i.e., accuracy, completeness and timeliness).	
WDM_2	Are there quality controls in place for when data from paper-based forms are entered into a computer (e.g., double entry, post-data entry verification, etc)?	
WDM_3	Is there a written back-up procedure for when data entry or data processing is computerized?	
WDM_4	If WDM_3 'yes', the latest date of back-up is appropriate given the frequency of update of the computerized system (e.g., back-ups are weekly or monthly).	
WDM_5	There is a written procedure to address late, incomplete, inaccurate and missing reports; including following-up with health facility on data quality issues.	
VI - Links with National Reporting System *(Yes=1, No=2)*		
WDM_6	The data are reported through a single channel of the national reporting system.	
WDM_7	The relevant national forms/tools are used for data-collection and reporting.	
VII – Use of Data for decision making *(Yes=1, No=2)*		
WDU_1D	The wereda develops charts, graphs, maps, etc …*(If yes, ask to see them)*	
WDU_2D	Staff at the wereda has access to guideline/technical assistance on data use (e.g. peer review meetings or during supervisory visits)	
WDU_3D	The analyzed data/results are presented/ disseminated to stakeholders in a timely manner so that the information can be used for informed decisions. (observe examples)	
SDS_31D	There are programmatic decision taken by the wereda based on the analyzed data *(see/observe examples of document)*	

w only

Data quality assessment tool for nuitrition indicators

Data quality assessment tools for *health center*

**1. Health center Checklist**		
**Person Interviewed (title)**		
**Health center Name**		
(Interviewer: Please verify if the following equipment is available in the Health center*)*		
**1. Equipment**		
1.1. Computer (give a number)	1. yes	0. no
1.2. Data Back-up Unit (e.g. CD, flash disc)	1. yes	0. no
1.3. Printers	1. yes	0. no
1.4. UPS	1. yes	0. no
1.5. Generators	1. yes	0. no
1.6. Regular telephone	1. yes	0. no
1.7. Access to the internet	1. yes	0. no
1.8. Calculator	1. yes	0. no
**2. Utilities**		
2.1. Is there an electricity supply?	1. yes	0. no
2.2. How often is the electricity supply interrupted?<br>0. Never/occasionally 1. Once a month 2. Twice a month 3. Weekly 4. Daily		
2.3. Is the room, where the computer hardware is kept, air-conditioned?	1. yes	0. no
**3. Availability of registers, forms**		
Type of record, report or register	Have you run out of this form in the past 6 month? If so, why?	
3.1 Integrated Management of Neonatal and Childhood Illnesses (IMNCI) register	1. yes<br>**Reasons:**	0. no
3.2. Comprehensive and Integrated Nutrition Service for <5 years children tally sheet	1. yes<br>**Reasons:**	0. no
3.3 Outpatient Therapeutic Program (OTP) card	1. yes<br>**Reasons:**	0. no
3.4 Stabilization Centre (SC) register	1. yes<br>**Reasons:**	0. no
3.5 Stabilization Centre (SC) tally sheet	1. yes<br>**Reasons:**	0. no
**4. Trained staff**		
Are there staff members who received any training in the recording, processing, or reporting of health information during the last two years? If yes;		
**4.1. HMIS**		
4.1.1. Health officer	1. yes	0. no
4.1.2. Nurse	1. yes	0. no
4.1.3. Health information technician (HIT)	1. yes	0. no
4.1.4. Other (specify)		
**4.2. CHIS**		
4.2.1. Health officer	1. yes	0. no
4.2.2. Nurse	1. yes	0. no
4.2.3. Health information technician (HIT)	1. yes	0. no
4.2.4. Other (specify)		
**4.3. DHIS2**		

For peer review only

For peer review only - http://bmjopen.bmj.com/site/about/guidelines.xhtml

4.3.1. Health officer	1. yes	0. no
4.3.2. Nurse	1. yes	0. no
4.3.3. Health information technician (HIT)	1. yes	0. no
4.3.4. Other (specify)		

Technical factors- Health center

18	Does a tally sheet exist?	1. Yes, Observed<br>0. No
19	Do data processing procedures exist?	1. Yes, Observed<br>0. No
20	Does the facility produce the following?	
20.1	Calculate nutrition indicators of the facility	1. Yes, Observed<br>0. No
20.2	Comparisons with Woreda targets	1. Yes, Observed<br>0. No
20.3	Comparisons with National targets	1. Yes, Observed<br>0. No
20.4	Comparisons among types of nutrition services coverage	1. Yes, Observed<br>0. No
20.5	Comparisons of nutrition services data over time (monitoring over time)	1. Yes, Observed<br>0. No
21	Does a procedure manual for nutrition services data collection (with definitions) exist?	1.Yes, Observed<br>0. No

**Data Completeness- at Health center level for the last six months**							
22	What is the number of Health posts in the catchment area that are supposed to report nutrition indicators?						
23	What is the number of Health posts in the catchment area that are actually reporting nutrition indicators						
24	How many nutrition data indicator is the health center supposed to receive on the HMIS monthly report?						
25	How many nutrition data indicator is the health center receive on the HMIS monthly report?						
26	How many nutrition indicators does the health center need to report on in the HMIS monthly report?						
27	Does the health center keep copies of HMIS monthly reports sent by health posts?	1. Yes<br>0. No					
28	Does the health center keep copies of HMIS monthly reports sent to the woreda health office?	1. Yes<br>0. No					
29	Count the number of monthly reports submitted by the health posts for the last 6 months	Month 1	Month 2	Month 3	Month 4	Month 5	Month 6
30		Month 1	Month 2	Month 3	Month 4	Month 5	Month 6

For peer review only - http://bmjopen.bmj.com/site/about/guidelines.xhtml

	Count the number of monthly reports submitted to the woreda health office by the health center for the last 6 months						
31	Does the health center fill the monthly report form completely	1.Yes, Observed<br>0. No					
32	Count the number of nutrition indicators that are supposed to be filled in by this facility but left blank without indicating "0" for the last 6 months reports						

**Data timeliness- at Health center level for the last six months**							
33	When are you expecting to receive the HMIS report from health posts?						
34	Does the health center record receipt dates of the HMIS monthly report?	1.Yes	0.No				
	If yes, check the dates of receipts for the last six months						
			HP 1	HP 2	HP 3	HP 4	HP 5
34.1	Month (specify)	1.Before deadline<br>0. After deadline					
34.2	Month (specify)	1.Before deadline<br>0. After deadline					
34.3	Month (specify)	1.Before deadline<br>0. After deadline					
34.4	Month (specify)	1.Before deadline<br>0. After deadline					
34.5	Month (specify)	1.Before deadline<br>0. After deadline					
34.6	Month (specify)	1.Before deadline<br>0. After deadline					
35	When are you expected to submit HMIS report to woreda health office?						
36	Does the health center have a record of submitting data on time to woreda level?	1.Yes<br>0.No					
	If yes, check the dates of submission for the last six months						
36.1	Month (specify)	1.Before deadline<br>0. After deadline					
36.2	Month (specify)	1.Before deadline<br>0. After deadline					
36.3	Month (specify)	1.Before deadline<br>0. After deadline					
36.4	Month (specify)	1.Before deadline<br>0. After deadline					
36.5	Month (specify)	1.Before deadline<br>0. After deadline					
36.6	Month (specify)	1.Before deadline<br>0. After deadline					

For peer review only - http://bmjopen.bmj.com/site/about/guidelines.xhtml

For peer review only

Data Accuracy Check- health center level for the last six months
Cluster report from all the health posts

37	Manually count the number of following data items from the HMIS monthly reports for the last 6 months. Compare the figures with the reports from the computer or paper database.								
		Indicator							
	Denominator								
	Month (specify)	Number of <5 children received VAS		Number of <5 children With SAM		Total number of children received GMP (< 2 years)		Number of children with two doses of Deworming (2-5 years)	
		HMIS report received from health post aggregated	HMIS report send to woreda health office	HMIS report received from health post aggregated	HMIS report send to woreda health office	HMIS report received from health post aggregated	HMIS report send to woreda health office	HMIS report received from health post aggregated	HMIS report send to woreda health office
37.1									
37.2									
37.3									
37.4									
37.5									
37.6									

Data Accuracy Check- health center level for the last six months Name of health post ……………………………….

38	Manually count the number of following data items from the HMIS monthly reports for the last 6 months. Compare the figures with the reports from the computer or paper database.								
		Indicator							
	Denominator								
	Month (specify)	Number of <5 children received VAS		Number of <5 children With SAM		Total number of children received GMP (< 2 years)		Number of children with two doses of Deworming (2-5 years)	
		HMIS report received from health post	HMIS report send to woreda health office	HMIS report received from health post	HMIS report send to woreda health office	HMIS report received from health post	HMIS report send to woreda health office	HMIS report received from health post	HMIS report send to woreda health office
38.1									
38.2									

For peer review only - http://bmjopen.bmj.com/site/about/guidelines.xhtml

38.3									
38.4									
38.5									
38.6									

For peer review only

For peer review only - http://bmjopen.bmj.com/site/about/guidelines.xhtml

Table 1 Coding framework

PRISM themes	Themes from ORCA	Codes
**Technical factors**	Denominator	• Wrong denominator<br>• Outdated denominator
	Complexity of forms and procedures for data recording and reporting	• Lack of guideline for recording<br>• Complexity of Indicator definitions<br>• Multiple forms<br>• Parallel reporting
	Language issues;	• Language barrier
**Behavioral Factors**	Knowledge and skills to manage HMIS	• understanding of health data<br>• Experience on data collection and reporting<br>• Lack of program knowledge<br>• Poor capacity on recording<br>• Low training<br>• Lack of skill operating computers
	Indicator definitions	• Knowledge on Health data and indicator definition
	motivation	• motivation
	Perception towards data quality and the level of emphasis given	• Perception towards data quality and the level of emphasis given
	Available interventions to improve Data quality and use,	• Available interventions to improve Data quality and use,
	Data use	• Data use
**Organizational Factors**	Communication between levels;	• Communication platform between the hierarchy<br>• Communication between departments<br>• HMIS Communication and feedback<br>• communication among HMIS, logistic and nutrition focal
	Monitoring,	• Routine data quality assessment<br>• Performance monitoring team
	supervision and evaluation	• feedback and learning<br>• HMIS data quality assurance methods<br>• Report evaluation practice
	Accountability	• Incentive mechanism
	Training: inadequate training,	• Capacity buildings<br>• Trained man power<br>• training for a wrong person
	Staff turnover,	• staff Turnover
	Staff retention	• attrition<br>• Staff rotation<br>• lack of HR resource
	Workload	• Workload

For peer review only

**Availability of resources**	Supply and availability of equipment for HMIS activity	• Shortage of HMIS tools
	Availability of source documents;	• HMIS budget, logistics and infrastructure<br>• transportation logistics
	Availability of computers and electricity	• availability of printing materials<br>• availability of computer<br>• availability of electricity
	Human resource for HMIS (shortage in number),	• Shortage of human resource for HMIS
	finance	• Finance
**RHIS Processes**	Data flow	• Data flow/Difference
	Data recording and reporting practice	• Wrong reporting/over reporting/under reporting
	Triangulation of data within routine health information system	• Different Reporting period<br>• Separate Logistics and TB Service reporting
	Completeness,	• double reporting during referral
	timeliness,	• Delay in reporting
	accuracy of reporting	• Source of data<br>• Reporting format difference (HC and HP)
		• Under report for private sector

For peer review only
